# A Rapid CRISPR/Cas-based Mutagenesis Assay in Zebrafish for Identification of Genes Involved in Thyroid Morphogenesis and Function

**DOI:** 10.1038/s41598-018-24036-4

**Published:** 2018-04-04

**Authors:** A. Trubiroha, P. Gillotay, N. Giusti, D. Gacquer, F. Libert, A. Lefort, B. Haerlingen, X. De Deken, R. Opitz, S. Costagliola

**Affiliations:** 1 Institute of Interdisciplinary Research in Molecular Human Biology (IRIBHM), Université Libre de Bruxelles, Route de Lennik 808, 1070 Brussels, Belgium; 2 Institute of Experimental Pediatric Endocrinology, Charité Universitätsmedizin Berlin, Augustenburger Platz 1, 13353 Berlin, Germany; 3 Present Address: German Federal Institute for Risk Assessment (BfR), Department Chemicals and Product Safety, Max-Dohrn-Strasse 8-10, 10589 Berlin, Germany

## Abstract

The foregut endoderm gives rise to several organs including liver, pancreas, lung and thyroid with important roles in human physiology. Understanding which genes and signalling pathways regulate their development is crucial for understanding developmental disorders as well as diseases in adulthood. We exploited unique advantages of the zebrafish model to develop a rapid and scalable CRISPR/Cas-based mutagenesis strategy aiming at the identification of genes involved in morphogenesis and function of the thyroid. Core elements of the mutagenesis assay comprise bi-allelic gene invalidation in somatic mutants, a non-invasive monitoring of thyroid development in live transgenic fish, complementary analyses of thyroid function in fixed specimens and quantitative analyses of mutagenesis efficiency by Illumina sequencing of individual fish. We successfully validated our mutagenesis-phenotyping strategy in experiments targeting genes with known functions in early thyroid morphogenesis (*pax2a*, *nkx2.4b*) and thyroid functional differentiation (*duox*, *duoxa*, *tshr*). We also demonstrate that *duox* and *duoxa* crispants phenocopy thyroid phenotypes previously observed in human patients with bi-allelic *DUOX2* and *DUOXA2* mutations. The proposed combination of efficient mutagenesis protocols, rapid non-invasive phenotyping and sensitive genotyping holds great potential to systematically characterize the function of larger candidate gene panels during thyroid development and is applicable to other organs and tissues.

## Introduction

During vertebrate embryonic development, the definitive endoderm transforms into a primitive gut tube from which various organ primordia will bud at predefined locations along the rostro-caudal axis^1^. The mammalian foregut endoderm, for example, gives rise to several organs with important physiological functions including liver, pancreas, stomach, lungs and thyroid. Identification of signalling pathways and intrinsic factors involved in the specification, morphogenesis and functional maturation of foregut-derived organs is crucial for understanding of the molecular mechanisms leading to developmental disorders in organogenesis and adult organ function^2^. This problem can be well exemplified with the case of developmental disorders in thyroid morphogenesis, commonly referred to as thyroid dysgenesis (TD), which can present as either thyroid ectopy, thyroid hypoplasia, athyreosis or hemiagenesis^3^. Although TD is the most prevalent cause of congenital hypothyroidism in human newborns, the pathogenic mechanisms underlying TD remained obscure in most cases^3,4^. One reason for this is our still limited understanding of the molecular signalling cascades orchestrating normal thyroid morphogenesis^5,6^.

At the molecular level, one important resource for the understanding of organogenesis and developmental disorders is knowledge about organ- and cell type-specific gene expression programs during critical developmental periods. In this respect, the introduction of next generation sequencing (NGS) techniques has allowed for unprecedented high-throughput transcriptome analyses of developing embryos, organs and tissues in diverse species^7–11^. In addition, the application of NGS in clinical research and diagnostics provided a plethora of genome-wide data on genetic variants and spurred the identification of new candidates for disease-causing genes^12,13^. However, up to date, the actual biological function during organ and tissue formation is often far from being clear for many of the newly identified genes.

Thus, one has to appreciate that information-rich analyses based on NGS techniques created new bottlenecks for developmental biologists including the need to identify rapid and scalable approaches to characterize the biological function for larger panels of candidate genes with hitherto unknown function during organ development. The most direct approach to study gene function is still the generation of loss-of-function models for a given gene followed by an analysis of developmental and functional phenotypes of the organ or tissue of interest. The recent development of the clustered regularly interspaced short palindromic repeats (CRISPR)-Cas system as a highly efficient genomic editing technology revolutionized the way new genetic models can be realized including the rapid production of mutant or knock-out animal models^14^. In combination with a suitable animal model, the CRISPR/Cas technique provides currently the most promising platform to facilitate rapid and scalable approaches towards a functional characterization of novel genes in a developmental context.

The zebrafish is one such powerful animal model to decipher cellular and molecular mechanisms of vertebrate organogenesis as it combines the genetic tractability of *Drosophila* with physiological and anatomical characteristics of higher vertebrates^15–17^. Several salient features make this model system particularly attractive for studies on gene function during early development. Foremost, zebrafish embryos and larvae develop externally and thus permit the monitoring of developmental processes and their pathological deviations in real time^18,19^. Further advantages include high fecundity, a rapid embryonic and larval development, and optical transparency of embryos and larvae. Importantly, a powerful molecular and genetic toolbox permits efficient transgenesis, real-time imaging of tissue and cell behaviours and forward and reverse genetic approaches to generate mutant models^19–22^.

In this study, we harnessed the advantages of the zebrafish model to develop a reverse genetics strategy to functionally characterize genes involved in thyroid development and function. Compared to other endoderm-derived organs such as liver and pancreas, our understanding of the molecular mechanisms underlying thyroid morphogenesis is much more limited. Although recent transcriptomic studies have disclosed gene expression patterns in foregut-derived organ buds including the thyroid^8,23^, little progress has been made in assigning specific functions to most of the newly identified genes during thyroid morphogenesis. This holds true for processes including thyroid precursor cell specification, thyroid bud formation, detachment and relocalization, early thyroid growth and functional maturation^6^. Most of our current knowledge about the development of functional thyroid tissue from endodermal precursors stems from studies in murine models but recent studies in zebrafish revealed that many aspects of thyroid morphogenesis are well conserved from fish to men^6,24^.

Stimulated by the high efficiency of the CRISPR/Cas9 system to generate biallelic gene disruption in F0 zebrafish embryos^25–27^, we combined optimized mutagenesis conditions with a novel phenotyping strategy and an information-rich genotyping approach into a reverse genetic screening protocol to identify genes involved in thyroid development and function. For this purpose, we generated a new zebrafish reporter line that permitted a rapid and sensitive detection of thyroid phenotypes related to thyroid dysgenesis as well as dyshormonogenesis in live fish. When applying this 6-day screening protocol to target loci encoding for genes with specific functions in thyroid development and function, we achieved a robust recovery of loss-of-function phenotypes in somatic mutants including cases of athyreosis, thyroid hypoplasia and goitrous thyroid enlargement.

## Results

### Monitoring of Thyroid Development in Live *Tg*(*tg:nlsEGFP*) Fish

**Figure 1:**
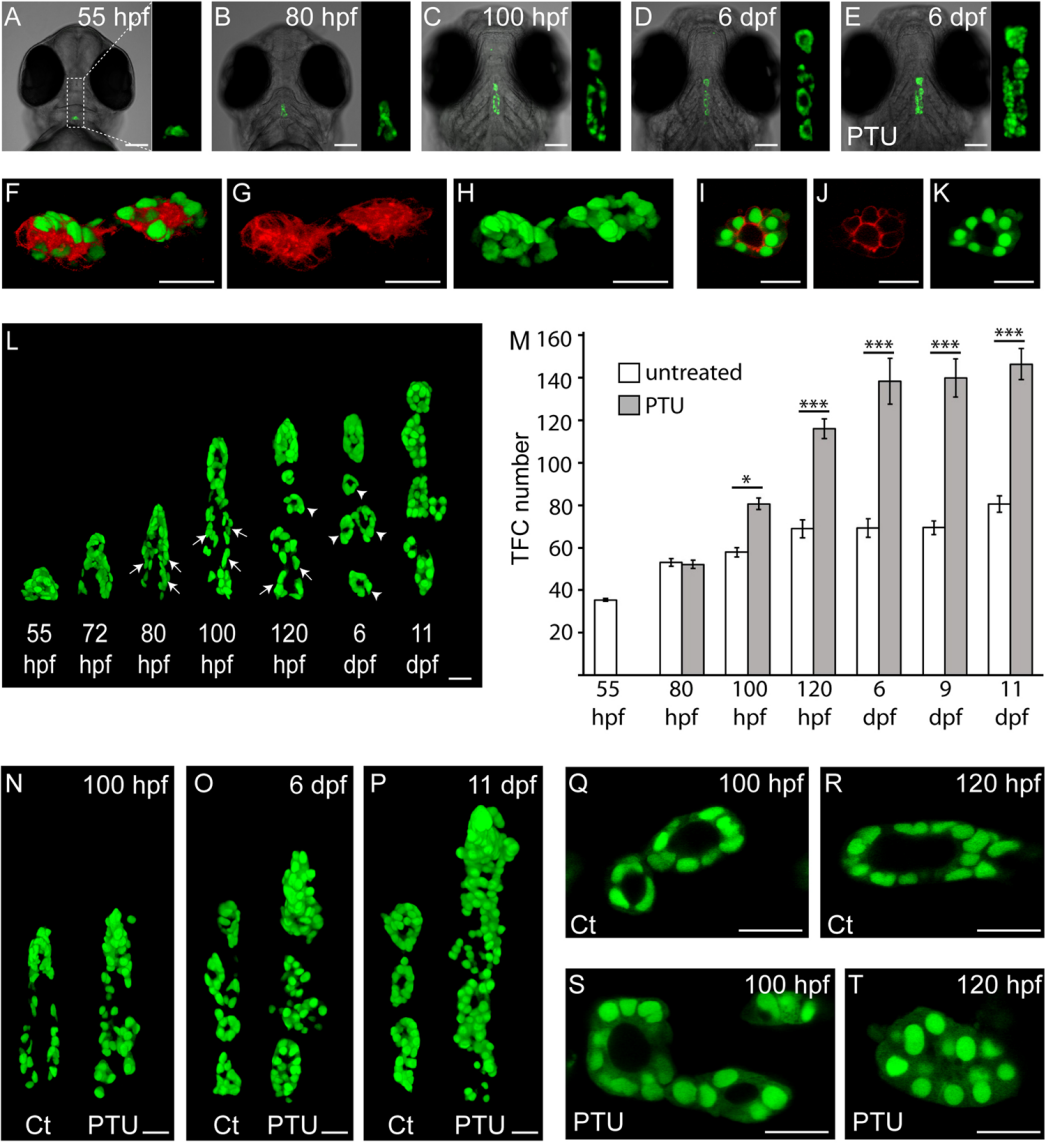
Live imaging of transgenic Tg(tg:nlsEGFP) zebrafish permits real-time analysis of thyroid morphogenesis. (**A**–**E**) Epifluorescence microscopy of live *Tg(tg:nlsEGFP)* zebrafish (ventral view, anterior is to the top) is sufficiently sensitive to monitor stage-dependent changes in thyroid morphology during normal thyroid development (see **A**–**D**) and to detect the goitrous thyroid enlargement caused by PTU treatment (**E**). For each embryo shown, three-fold magnified views of the thyroid region are displayed (GFP channel). (**F**–**K**) Confocal analyses of double transgenic *Tg(tg:mCherry;tg:nlsEGFP)* larvae (100 hpf) confirms thyroid-specific co-expression of membrane-tethered mCherry (**F,G,I,J**) and nuclear EGFP reporters (**F,H,I,K**). 3D reconstruction of confocal images (**F–H**) and individual confocal sections (**I–K**) are shown. Ventral view, anterior is to the left. (**L**) Live imaging of a developmental series of *Tg(tg:nlsEGFP)* fish by confocal microscopy highlights the progressive expansion of thyroid tissue along the anterio-posterior axis (ventral view, anterior is to the top), the increase in thyroid cell number and the re-organization of cord-like cell clusters (arrows) into definitive follicular structures (arrowheads) during normal development. (**M**) Quantification of thyroid follicular cell (TFC) number in untreated control and PTU-treated zebrafish. Results are shown as means ± S.E.M (*N* = 6–10). Asterisks denote significant differences between treatment means at a given developmental stage (*P < 0.05, ***P < 0.001, Student’s *t*-test). (**N–P**) Comparison of thyroid morphologies between untreated control (Ct) and PTU-treated fish at different time point during the course of PTU treatment. Note the increased size and hyperplasia of thyroids from PTU-treated fish at 6 and 11 dpf. 3D reconstruction of confocal images obtained in live fish are shown (ventral view, anterior is to the top). (**Q–T**) Confocal sections highlight the stage-dependent increases in follicle size and luminal diameter in untreated control (Ct) fish (see **Q,R**). In PTU-treated fish, live imaging (ventral view, anterior is to the left) permitted monitoring of goitrous responses including early onset of thyroid cell hypertrophy (**S**), collapse of follicular lumina (**T**) and thyroid cell hyperplasia (**T**). Scale bars: 100 µm (**A–E**) and 20 µm (**F–L, N–T**).

Transgenic zebrafish expressing fluorescent reporters in specific cell types, tissues or organs are powerful tools for phenotypic analyses of mutagenized embryos^28,29^. This is particularly true for small organs, such as the zebrafish thyroid, which are hardly detectable by brightfield microscopy of live embryos. To exploit the advantageous properties of transgenic zebrafish, we generated a novel reporter line expressing a nuclear-located EGFP (nlsEGFP) variant under the control of the zebrafish *thyroglobulin* (*tg*) promoter^30^. The *Tg(tg:nlsEGFP*)^*ulb4*^ line showed very promising properties for sensitive analyses of thyroid morphogenesis in live embryos by using various microscopic modalities (Fig. 1). As reported previously for the *Tg(tg:mCherry*)^*ulb1*^ line, embryos of the *Tg(tg:nlsEGFP)* line showed an early onset of reporter expression around 36 hpf and EGFP expression was restricted to thyroid follicular cells (TFC) at all developmental stages examined (Fig. 1A–D and data not shown). Confocal microscopic analyses of double transgenic fish obtained from crosses of *Tg(tg:nlsEGFP)* and *Tg(tg:mCherry)* fish confirmed identical spatiotemporal expression patterns of both reporters yet distinct subcellular expression domains (Fig. 1F–K and data not shown).

We used *Tg(tg:nlsEGFP)* embryos and larvae for confocal live imaging to characterize changes in thyroid morphology during thyroid development from 55 hpf to 11 dpf and established a three-dimensional atlas of normal thyroid development (Fig. 1L). These analyses identified distinct phases of late thyroid organogenesis. During an early growth phase between 55 and 100 hpf, the initially compact thyroid primordium rapidly expands along the pharyngeal midline (see Fig. 1A–D and L). The zebrafish thyroid displays a unique morphology at these stages (see Fig. 1L). Rostrally, most thyroid cells are already organized in follicular structures which increase in size over time (Fig. 1Q,R). More caudally, however, the thyroid consists of bilateral, seemingly irregular strands of thyroid cells and confocal microscopy revealed that thyroid cells are arranged as cell cords in the caudal region (Fig. 1L). Concurrent with the elongation of the bilateral cellular cords along the anterior-posterior axis, a segmentation of cords into small follicular structures occurs between 80 and 100 hpf (Fig. 1L). These small follicular structures often comprise only 3–4 thyroid cells arranged around a luminal cavity. For most larvae, a complete re-organization of cell cords into distinct follicles was observed by 6 dpf (Fig. 1L). Between 6 and 11 dpf, thyroid follicles continue to increase in size while the total number of thyroid follicles (typically 4–6 follicles) remains relatively stable.

Taking advantage of the nuclear expression of EGFP, we next determined developmental changes in TFC number based on images acquired during confocal live imaging (Fig. 1M). These analyses showed that thyroid expansion between 55 and 120 hpf is associated with a doubling in TFC number from approximately 35 cells to about 70 cells. Between 120 hpf and 9 dpf, we observed, if any, only a minimal increase in TFC number while a moderate increase in TFC number was detectable between 9 and 11 dpf (Fig. 1M).

### Effects of PTU and T4 Treatment on Thyroid Development

Thyroid-stimulating hormone (TSH), secreted by pituitary thyrotrophic cells, is the primary physiological regulator of vertebrate thyroid function and growth^31^. In order to characterize the effects of TSH hyper- and hypostimulation on zebrafish thyroid development, we treated *Tg(tg:nlsEGFP)* embryos and larvae from 52 hpf with either 30 mg/L phenylthiourea (PTU) or 5.0 µg/L thyroxine (T4) and monitored thyroid morphogenesis and thyroid growth in treated *versus* untreated fish (controls) by confocal live imaging.

Treatment of zebrafish larvae with PTU inhibits thyroid hormone (TH) synthesis, causes increased pituitary *tshb* expression and results in enhanced TSH stimulation of thyroid tissue^32,33^. Here, we demonstrate that PTU treatment induces a goitrous thyroid enlargement in zebrafish larvae that was readily detectable by live imaging of *Tg(tg:nlsEGFP)* larvae at 6 dpf (Fig. 1E). Notably, the effects of PTU treatment on thyroid morphology were detectable using microscopic equipment such as epifluorescence microscopes and fluorescent stereomicroscopes that is amenable to higher throughput screening approaches.

We next used confocal live imaging to analyze the temporal evolution of the PTU-induced thyroid enlargement (Fig. 1N–P). Prior to the reported onset of endogenous TH synthesis at around 80 hpf^32,33^, thyroids of PTU-treated larvae did not differ from controls in terms of overall size and shape, TFC number and follicular morphology (Fig. 1M and data not shown). However, as early as 100 hpf, PTU-treated larvae showed a statistically significant increase in TFC number compared to control larvae (Fig. 1M,N) and the follicular epithelium became hypertrophic (see Fig. 1Q,S). By 120 hpf, thyroids of PTU-treated larvae had a greatly increased TFC number compared to controls (Fig. 1M) and displayed TFC hypertrophy/hyperplasia together with an almost complete collapse of luminal compartments (see Fig. 1R,T). Between 6 and 11 dpf, thyroids of PTU-treated larvae were grossly enlarged (Fig. 1O,P) and contained twice as much TFC compared to control thyroids (Fig. 1M).

Along with the aforementioned PTU-induced changes in thyroid morphology, the intensity of the total reporter signal in the thyroid region was greatly increased and quantitative measurements of reporter signal intensities in PTU-treated and untreated larvae provided for an alternative means to sensitively detect thyroidal responses to PTU treatment (Supplementary Fig. 1). Notably, the 2.4-fold increase in reporter signal intensities detected in PTU-treated larvae at 6 dpf correlated closely with the approximately 2-fold increase in TFC number, indicating that the increase in TFC number accounts for much of the observed increase in reporter signal intensity.

**Figure 2:**
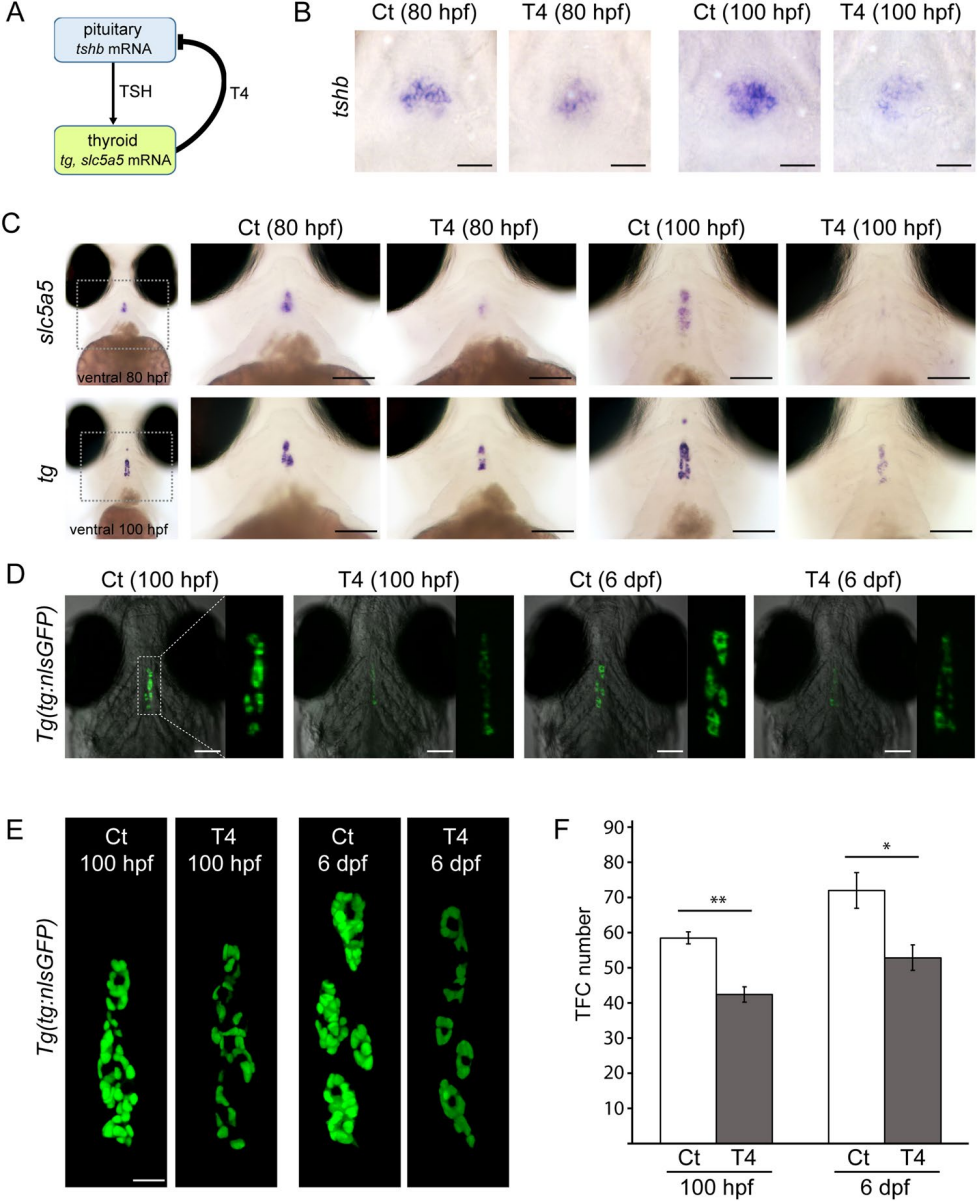
Live imaging of transgenic *Tg(tg:nlsEGFP)* fish reveals diminished reporter expression and thyroid hypoplasia in thyroxine-treated fish. (**A**) Model for thyroid hormone negative feedback loop along pituitary-thyroid axis with tissue-specific gene expression markers examined in this study. T4, thyroxine; TSH, thyroid-stimulating hormone. (**B,C**) Treatment of zebrafish embryos with 5.0 µg/L T4 affects pituitary and thyroid gene expression as demonstrated by WISH. Pituitary expression of *tshb* mRNA (see ventral views in B) is reduced in T4-treated fish compared to untreated controls (Ct). At the thyroid level, expression of the TSH-dependent gene *slc5a5* is strongly decreased following T4 treatment (ventral views in C, upper panels). T4-treated fish also showed decreased *tg* mRNA expression and a reduced number of *tg* mRNA expressing cells (ventral views in C, lower panels), effects which were more marked at 100 hpf. (**D**) Epifluorescence microscopy of live *Tg(tg:nlsEGFP)* fish revealed a strongly reduced intensity of the fluorescent reporter signal in T4-treated fish relative to untreated controls (Ct). For each embryo shown (ventral view), three-fold magnified views of the thyroid region are displayed (GFP channel). (**E**) Comparative analysis of thyroid morphology in control (Ct) and T4-treated larvae by confocal live imaging of *g(tg:nlsEGFP)* fish. 3D reconstructions of confocal images (ventral views) are shown. At 6 dpf, thyroids of T4-treated fish appeared atrophic (smaller follicles and reduced reporter expression). (**F**) Quantification of thyroid follicular cell (TFC) number in untreated controls (Ct) and T4-treated fish. Results are shown as means ± S.E.M (*N* = X − Y). Asterisks denote significant differences between treatment means at the indicated developmental stages (*P < 0.05, **P < 0.01, Student’s t-test). Anterior is to the top in all images. Scale bars: 20 µm (**B**,**E**) and 100 µm (**C**,**D**).

Treatment with T4 reportedly suppresses pituitary *tshb* mRNA expression in zebrafish larvae^34,35^ offering a model to characterize possible consequences of diminished TSH stimulation on the developing thyroid tissue. In this study, we treated zebrafish with a concentration of 5.0 µg/L T4 from 52 hpf and used whole-mount *in situ* hybridization (WISH) to assess T4 treatment effects on feedback signalling along the pituitary-thyroid axis in this experimental model (Fig. 2). Consistent with the classical negative feedback model (Fig. 2A), we observed a suppression of pituitary *tshb* mRNA expression in T4-treated embryos (Fig. 2B) and a concurrent strong reduction in thyroidal mRNA expression levels for the TSH-dependent gene *slc5a5* (Fig. 2C). Effects of T4 treatment on *tshb* and *slc5a5* expression were more pronounced at 100 hpf compared to 80 hpf. Analysis of T4 effects on *tg* expression revealed reduced mRNA expression levels in thyroid cells as well as a moderate decrease in the number of *tg* mRNA-expressing cells at 100 hpf.

Live imaging of *Tg(tg:nlsEGFP)* embryos and larvae raised in the presence or absence of T4 closely recapitulated observations made for endogenous *tg* mRNA expression (Fig. 2D–F). Similar to the treatment studies with PTU, T4-treated *Tg(tg:nlsEGFP)* larvae displayed first marked differences in thyroid reporter expression at 100 hpf. At this stage, the reporter signal was very dim in T4-treated larvae (Fig. 2D) and confocal analyses revealed a significantly reduced number of TFC in thyroids of T4-treated larvae (Fig. 2F). Although controls and T4-treated fish showed a similar developmental timing in follicle formation, our confocal analyses of 6 dpf larvae revealed that follicles of T4-treated larvae were smaller and contained less TFC compared to untreated controls (Fig. 2E). The atrophic/hypoplastic thyroid phenotype resulting from T4 treatment was readily detectable under a fluorescent stereomicroscope as a grossly visible reduction of fluorescent reporter expression in T4 treated larvae compared to untreated larvae (Fig. 2D). Quantitative analysis of reporter signal intensities independently confirmed that total reporter signals in the thyroid region are reduced by approximately 80% in T4 treated larvae at 6 dpf (Supplementary Fig. 1). Given that the TFC number was reduced by approximately 26% in T4-treated larvae at 6 dpf (Fig. 2F), these analyses indicate that a great part of the decrease in total reporter signals was likely due to the reduced levels of cellular reporter expression. Collectively, our analyses of normal thyroid development and the phenotypic alterations induced by PTU and T4 demonstrate that monitoring of live *Tg(tg:nlsEGFP)* embryos provides a sensitive and rapid means of thyroid phenotyping.

### Protocol for Thyroid Phenotyping and CRISPR/Cas9 Somatic Mutagenesis

Based on the aforementioned observations, we devised a 6-day phenotyping protocol that relies on repeated non-invasive live analysis of thyroid development in control and crispant *Tg(tg:nlsEGFP)* fish over a period of 6 days in combination with functional assays performed in fixed 6 dpf larvae. For the monitoring of live *Tg(tg:nlsEGFP)* embryos and larvae, we considered 55 hpf, 80 hpf and 6 dpf as relevant developmental stages to detect possible effects on early thyroid morphogenesis (55 hpf), growth expansion (80 hpf) and folliculogenesis (6 dpf). For the present study, we further selected whole-mount immunofluorescence (WIF) of colloidal T4 as an integrative endpoint to characterize thyroidal function in fish sampled at 6 dpf^33,36^. In addition, all thyroid phenotyping was done for fish that are not affected in their gross development (with the exception of *pax2a* crispants, see below). Notably, meaningful thyroid phenotyping according to the criteria outlined above relies on a fairly normal development and growth of the experimental fish and particularly on the absence of gross dysmorphogenic alterations of the pharyngeal region (see Supplementary Fig. 10A).

The mutagenesis protocol used in this study was based on previous reports that injection of active Cas9/sgRNA ribonucleoprotein complex can achieve sufficiently high levels of bi-allelic invalidation of target genes to recover developmental phenotypes directly in F0 fish^25–27^. Injection experiments with the various sgRNAs used in this study showed that microinjection of 300 pg Cas9 protein together with up to 200–250 pg sgRNA resulted in mortality rates ranging between 8.1 and 20% of injected embryos. Across all injection experiments, a mean mortality rate of 12.5% was observed in injected embryos. For comparison, mortality rates in non-injected control embryo groups ranged between 4.3 and 9.2% with a mean mortality rate of 5.1% across all experiments. In this study, cumulative embryo mortality was determined from 24 hpf onwards and, therefore, comprised non-fertilized eggs in mortality counts. Given that our mutagenesis protocol included injection of at least 150 embryos per sgRNA, we considered injection-associated toxicities tolerable as long as more than 70% of the injected embryos (at least 100 embryos) were available for phenotypic assessment. All sgRNAs used in this study fulfilled these criteria. Moreover, the far majority of fish injected with different sgRNAs displayed normal morphological development and growth up to 6 dpf (Supplementary Fig. 10A).

### Thyroid Dysgenesis Phenotypes in *pax2a* and *nkx2.4b* Crispants

The zebrafish *pax2a* (ZDB-GENE-990415-8) and *nkx2.4b* (ZDB-GENE-000830-1) genes encode for transcription factors which act as key regulators of early thyroid morphogenesis^37,38^. Similar to their mammalian counterparts *Pax8* and *Nkx2.1*^5^, zebrafish models with impaired function of *pax2a* (*no isthmus* mutant, *noi*) and *nkx2.4b* (transient gene knock-down by morpholino injection) display defects in early thyroid morphogenesis resulting in athyreosis^37,38^.

**Figure 3:**
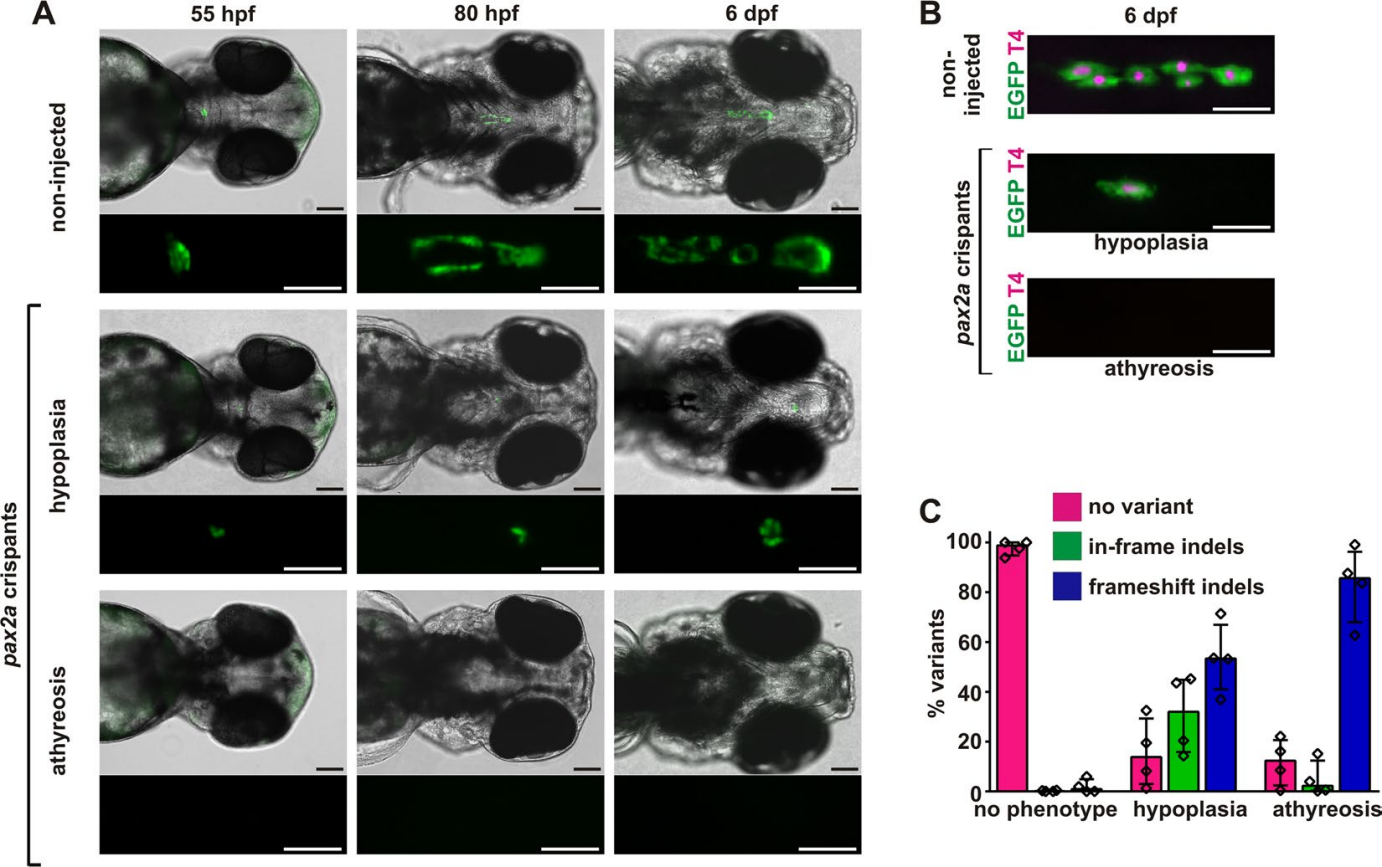
Recovery of thyroid dysgenesis phenotypes in zebrafish pax2a crispants. (**A**) Epifluorescence live imaging of Tg(tg:nlsEGFP) zebrafish. Ventral views of the head region (anterior to the right, scale bar: 100 µm) and magnified views of the thyroid region (GFP channel only, scale bar: 50 µm) are shown for non-injected controls and *pax2a* crispants at 55 hpf, 80 hpf, and 6 dpf. Phenotypic *pax2a* crispants displayed athyreosis or severe hypoplasia. (**B**) Whole-mount immunofluorescence staining of *Tg*(*tg:nlsEGFP*) zebrafish (6 dpf) for EGFP (thyroid cells) and thyroxine (colloidal T4). Epifluorescence imaging of the thyroid region in 6 dpf larvae (ventral views, anterior to the right, scale bar: 100 µm) revealed variable but reduced T4 content in *pax2a* crispants with thyroid hypoplasia and confirmed the absence of detectable T4 synthesis in *pax2a* crispants lacking EGFP-tagged thyroid cells (athyreosis group). (**C**) Distribution of allelic variants as determined by Illumina HiSeq analysis of individual *pax2a* crispants confirmed high mutagenesis efficiency in larvae affected by thyroid dysgenesis. The percentage of WT alleles (no variant call), in-frame indels, or frameshift indels is shown for *N* = 4 larvae per phenotypic category (median values with interquartile range).

In this study, we targeted the paired box domain region in exon 2 of *pax2a* and the homeodomain region in exon 2 of *nkx2.4b* with specific sgRNAs and assessed whether we can recover thyroid phenotypes in somatic mutants. When analysed at 55 hpf, many *Tg(tg:nlsEGFP)* embryos injected with sgRNAs targeting either *pax2a* or *nkx2.4b* displayed complete absence of the thyroid (athyreosis) or severe thyroid hypoplasia (Fig. 3 and Supplementary Fig. 2). Several *pax2a* crispants also displayed gross morphological abnormalities, including edema of the pericardium and gut epithelium and defective brain development (Supplementary Fig. 10A), similar to what has previously been described for strong *noi* alleles^39^. Because cardiac edema causes severe perturbation of pharyngeal development, we removed these *pax2a* crispants from the final analyses of thyroid phenotyping. In the *pax2a* experiments, approximately 50% of injected embryos presented with a grossly visible thyroid dysgenesis phenotype (Table 1). Notably, 30% of *pax2a* crispants showed athyreosis (absence of detectable EGFP reporter expression), thus phenocopying the thyroid phenotype described in *noi* mutants^38^. To further compare thyroid phenotypes between *noi* mutants and *pax2a* crispants, we analysed thyroid marker expression in *pax2a* crispants at 28 hpf (*nkx2.4b*) and 55 hpf (*tg*) by WISH. Consistent with previous studies of *noi* mutants^37,38^, we found that the early thyroid anlage was formed in all *pax2a* crispants (Supplementary Fig. A,B) but expression of thyroid markers becomes severely impaired or is absent in about half of the *pax2a* crispants analysed at 55 hpf (Supplementary Fig. 3C).

**Table 1:** Thyroid phenotypes detected in F0 crispant larvae.

**dysgenesis phenotypes**				
target: *nkx2.4b* exon 1				
	non-injected	crispants		
phenotype	—	none	hypoplasia	athyreosis
N (%)	66	91 (53.2)	55 (32.2)	25 (14.6)
target: *nkx2.4b* exon 2				
	non-injected	crispants		
phenotype	—	none	hypoplasia	athyreosis
N (%)	83	207 (51.2)	116 (28.7)	81 (20.0)
target: *pax2a* exon 2				
	non-injected	crispants		
phenotype	—	none	hypoplasia	athyreosis
N (%)	105	118 (50.9)	43 (18.5)	71 (30.6)
**dyshormonogenesis phenotypes**				
target: *tshr* exon 4				
	non-injected	crispants		
phenotype	—	none	hypoplasia, decreased T4	
N (%)	145	191 (45.5)	229 (54.5)	
target: *tshr* exon 10				
	non-injected	crispants		
phenotype	—	none	hypoplasia, decreased T4	
N (%)	62	104 (60.1)	69 (39.9)	
target: *duox* exon 23				
	non-injected	crispants		
phenotype	—	none	decreased T4	hyperplasia/decreased T4
N (%)	47	29 (16.6)	15 (8.6)	131 (74.9)
target: *duoxa* exon 2				
	non-injected	crispants		
phenotype	—	none	decreased T4	hyperplasia/decreased T4
N (%)	138	74 (49.7)	26 (17.5)	49 (32.9)

Similar to *pax2a*, injection of a sgRNA targetting exon 2 of *nkx2.4b* resulted in approximately 50% of embryos with a grossly visible thyroid dysgenesis phenotype although the proportion of fish with complete athyreosis (20%) was slightly lower (Table 1, Supplementary Fig. 2). We obtained similar results with an alternative sgRNA targeting exon 1 of *nkx2.4b* (Table 1, Supplementary Fig. 2). The thyroid dysgenesis phenotypes detected at 55 hpf in *pax2a* and *nkx2.4b* crispants persisted until 6 dpf (Fig. 3 and Supplementary Fig. 2) ruling out that observations made at 55 hpf were merely due to a developmental delay. Transgenic larvae with complete absence of thyroid reporter expression never showed any detectable signal in the colloidal T4 immunofluorescence assay corroborating the absence of functional thyroid tissue. This was true for all *pax2a* and *nkx2.4b* crispants lacking thyroid reporter expression in this study. In contrast, a variable though greatly reduced T4 immunofluorescence signal was detectable in some *pax2a* and *nkx2.4b* crispants with thyroid hypoplasia indicating the formation of functional thyroid follicles (Fig. 3B and Supplementary Fig. 2). Collectively, these data demonstrate that thyroid dysgenesis phenotypes can be recovered in somatic mutants and are readily detectable by analysis of live reporter embryos at 55 hpf.

Upon completion of thyroid phenotyping (including T4 immunofluorescence), we extracted genomic DNA from whole larvae and assessed the mutagenesis efficiency for at least 4 individual crispant larvae from each phenotypic category (athyreosis, thyroid hypoplasia, no grossly visible thyroid phenotype) using Illumina HiSeq technology. Total numbers of HiSeq-derived sequences, mapped reads and allele frequencies for individual larvae are provided as Supplementary Information 2. Sequencing of *pax2a* and *nkx2.4b* crispants displaying athyreosis confirmed a high mutagenesis efficiency of the sgRNAs used in this study (Fig. 3C, Supplementary Fig. 6 and Supplementary information 2 - NGS Data). Given that frameshift indels have a high likelihood to create loss-of-function alleles, it was of particular interest that the 44,900–89,088 mapped sequence reads obtained from individual athyroid *pax2a* crispants contained particularly high frequencies of frameshift indels ranging from 64.6% (57,593/89,088 reads) up to 98.5% (84,490/85,790 reads). When comparing indel allele frequencies between individual larvae displaying athyreosis and hypoplasia, we observed a reduced frequency of frameshift alleles in *pax2a* crispants displaying less severe thyroid phenotypes (hypoplasia) whereas no such tendency was apparent from the sequencing of *nkx2.4b* crispants (Fig. 3C and Supplementary Fig. 6). Sequencing of *pax2a* crispants displaying no discernible thyroid phenotype detected predominantly wild-type (WT) alleles. The latter observations strengthen the notion that our phenotyping strategy provides high sensitivity to recover phenotypes linked to thyroid dysgenesis. In the case of *pax2a* crispants we additionally confirmed effective frameshift mutagenesis at the protein level by demonstrating strongly reduced pax2a immunoreactivity using an antibody directed against an C-terminal epitope (Supplementary Fig. 3D).

### *duox* and *duoxa* Crispants Develop Dyshormonogenic Goiter

We next addressed the question whether our mutagenesis assay can sensitively recover phenotypes arising from disturbed TH synthesis. For this purpose, we targeted zebrafish orthologues of mammalian *DUOX1/2* and *DUOXA1/2* genes. In the mammalian thyroid, heterodimeric DUOX/DUOXA complexes generate the H_2_O_2_ required for TH synthesis and genetic alterations of the *DUOX/DUOXA* system can lead to congenital hypothyroidism^40^. In contrast to mammals, the zebrafish genome contains only a single *duox* gene (ZDB-GENE-091117-14) and a single *duoxa* gene (ZDB-GENE-111007-1, currently mis-annotated as *duox2*). To date, functional zebrafish models were limited to morpholino-mediated knockdown of *duox* gene function and the assessment of developmental processes unrelated to thyroid function^41^.

**Figure 4:**
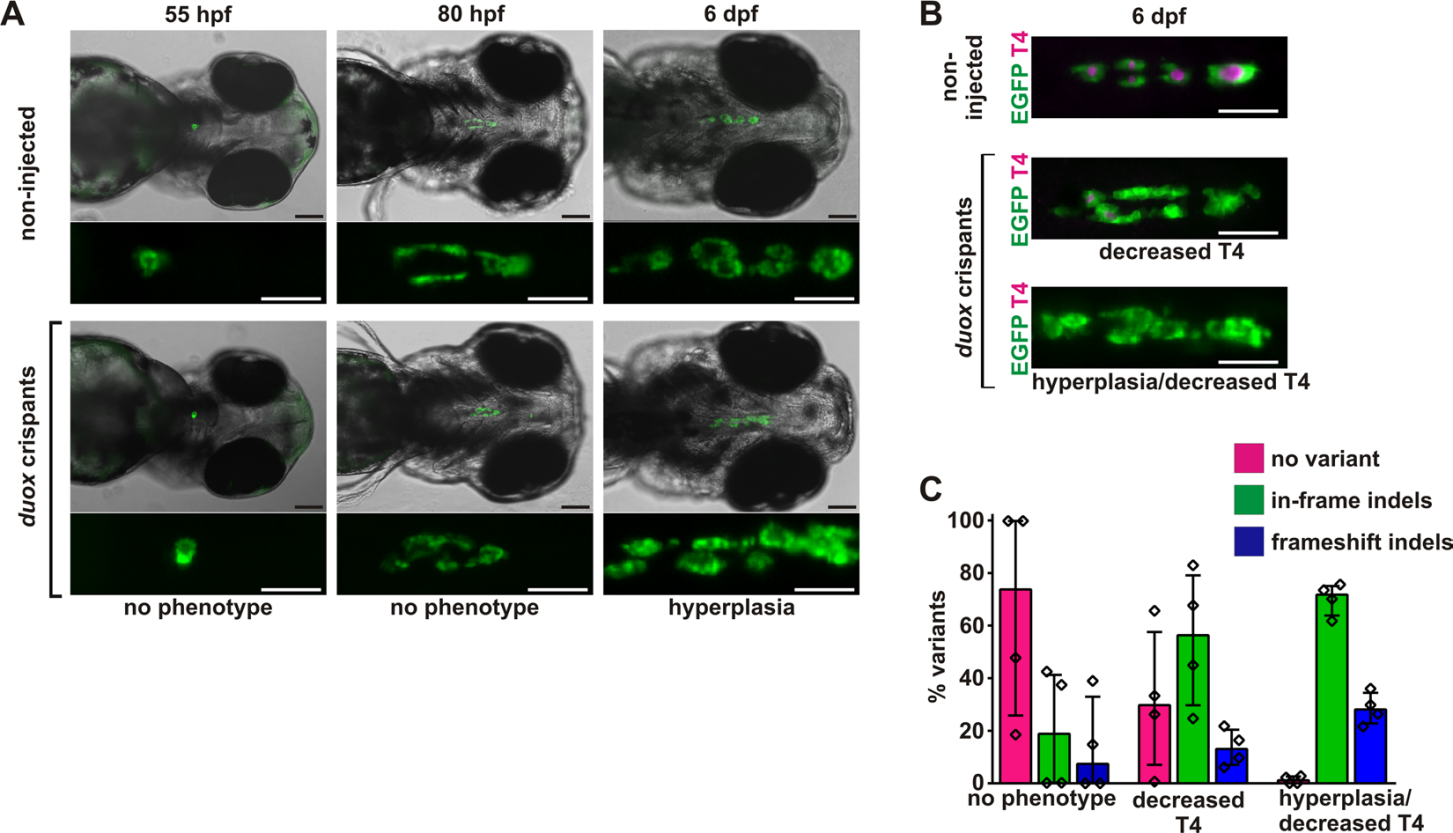
Recovery of dyshormonogenesis phenotypes in zebrafish duox crispants. (**A**) Epifluorescence live imaging of *Tg*(*tg:nlsEGFP*) zebrafish. Ventral views of the head region (anterior to the right, scale bar: 100 µm) and magnified views of the thyroid region (GFP channel only, scale bar: 50 µm) are shown for non-injected controls and *duox* crispants at 55 hpf, 80 hpf, and 6 dpf. No thyroid phenotypes were detectable at 55 hpf and 80 hpf but by 6 dpf, the majority of *duox* crispants displayed a goitrous thyroid enlargement with severe hyperplasia. (**B**) Whole-mount immunofluorescence staining of *Tg*(*tg:nlsEGFP*) zebrafish (6 dpf) for EGFP (thyroid cells) and thyroxine (colloidal T4). Epifluorescence imaging of the thyroid region in 6 dpf larvae (ventral views, anterior to the right, scale bar: 50 µm) revealed reduced T4 content in *duox* crispants as the most prevalent phenotypic alterations. Most of these hypothyroid larvae displayed concurrent thyroid hyperplasia. (**C**) Distribution of allelic variants as determined by Illumina HiSeq analysis of individual *duox* crispants confirmed high mutagenesis efficiency in larvae affected by thyroid dyshormonogenesis. The percentage of WT alleles (no variant call), in-frame indels, or frameshift indels is shown for *N* = 4 larvae per phenotypic category (median values with interquartile range).

In this study, we targeted a sequence in exon 23 of the *duox* gene. For zebrafish *duoxa*, we targeted a region close to the N-terminus of the protein to maximize the likelihood that frameshifts create a non-functional allele. When assessed at 55 and 80 hpf, neither *duox* nor *duoxa* crispants presented gross thyroid phenotypes (Fig. 4 and Supplementary Fig. 4). This finding is consistent with the reported role of both genes in thyroid function but not morphogenesis^40^. In contrast, analyses of live fish at 6 dpf revealed a high proportion of *duox* and *duoxa* crispants displaying a thyroid phenotype resembling the goitrous thyroid enlargement induced by PTU treatment (Fig. 4 and Supplementary Fig. 4). While the prevalence of the hyperplastic goiter phenotype was higher in *duox* crispants (detected in 75% of injected embryos) than in *duoxa* crispants (see Table 1), severity and morphological characteristics of the thyroid alterations were similar in both mutant models. Consistent with a dyshormonogenetic goiter classification, T4 immunofluorescence analyses revealed if any only a very faint T4 staining in crispants with goitrous thyroids (Fig. 4B and Supplementary Fig. 4). The T4 assay also identified a minor subpopulation of *duox* and *duoxa* crispants displaying a thyroid phenotype characterized by reduced colloidal T4 content but fairly normal thyroid morphology (absence of gross thyroid enlargement). Thus, the T4 immunofluorescence assay contributed to the overall sensitivity of the mutagenesis assay by recovering additional milder phenotypic variants. In conjunction with the thyroid phenotypes observed following chemical blockade of TH synthesis, the results from *duox* and *duoxa* crispant analyses corroborate the capacity of our mutagenesis-phenotyping strategy to sensitively detect genetic alterations that impair thyroidal TH synthesis.

Illumina sequencing of individual *duox* and *duoxa* crispants displaying dyshormonogenetic goiter confirmed a high mutagenesis efficiency of the sgRNAs used in this study (Fig. 4C and Supplementary Fig. 6). Total numbers of HiSeq-derived sequences, mapped reads and allele frequencies for *duox* and *duoxa* crispants are provided as Supplementary Information 2. Interestingly, for *duox* crispants, we observed predominantly in-frame indels in both severe and milder phenotypic groups (Fig. 4C and Supplementary Information 2- NGS Data). For crispants displaying a severe thyroid phenotype, the frequency of in-frame indels among mapped sequences ranged between 50.3% (49,009/97,458 mapped reads) and 71.2% (48,900/68,708 mapped reads). Although we cannot predict the functional consequences of the various in-frame indels for duox function, our data suggest that the targeted region in exon 23 is of functional importance and might not tolerate sequence alterations without compromising protein function. When comparing mutant allele frequencies recovered from individual larvae displaying hyperplastic thyroids with reduced T4 synthesis or normally sized thyroids with reduced T4 synthesis, we observed a reduced frequency of mutant alleles in *duox* crispants displaying less severe thyroid phenotypes whereas no such correlation was apparent from the sequencing of *duoxa* crispants (see Supplementary NGS Data).

### Hypofunctional Thyroid Phenotypes and TSH Resistance in *tshr* Crispants

Regulation of thyroid function by TSH is mediated via binding of TSH to its cognate membrane receptor (TSHR) and subsequent activation of several intracellular second messenger systems^42^. Murine *Tshr* knock-out models display hypothyroidism and develop thyroid hypoplasia in the postnatal period^43^. Morpholino knock-down of zebrafish *tshr* does not affect early thyroid morphogenesis but *tshr* morphants show a hypofunctional thyroid phenotype^33^. Against this background information, we targeted regions in exon 4 and exon 10 of the zebrafish *tshr* gene (ZDB-GENE-110524-4), respectively, and assessed the capacity of our mutagenesis-phenotyping strategy to recover mutant phenotypes.

**Figure 5:**
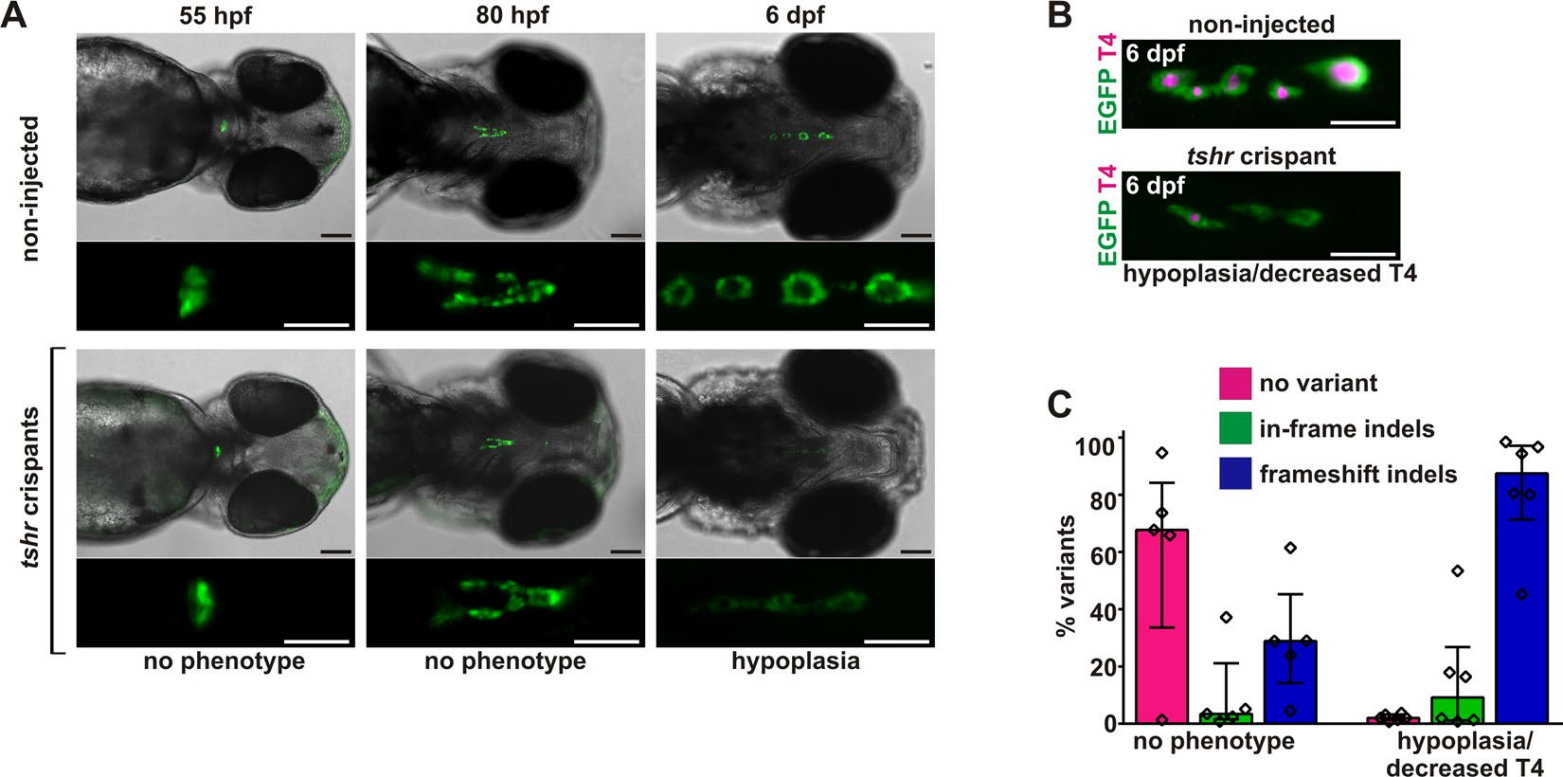
Recovery of hypoplastic/atrophic thyroid phenotypes in zebrafish tshr crispants. (**A**) Epifluorescence live imaging of *Tg*(*tg:nlsEGFP*) zebrafish. Ventral views of the head region (anterior to the right, scale bar: 100 µm) and magnified views of the thyroid region (GFP channel only, scale bar: 50 µm) are shown for non-injected controls and *tshr* crispants (target: exon 4) at 55 hpf, 80 hpf, and 6 dpf. No thyroid phenotypes were detectable at 55 hpf and 80 hpf but by 6 dpf, *tshr* crispants presented a hypoplastic/atrophic thyroid phenotype. (**B**) Whole-mount immunofluorescence staining of *Tg*(*tg:nlsEGFP*) zebrafish (6 dpf) for EGFP (thyroid cells) and thyroxine (colloidal T4). Epifluorescence imaging of the thyroid region in 6 dpf larvae (ventral views, anterior to the right, scale bar: 50 µm) revealed that thyroid hypoplasia in *tshr* crispants was accompanied by a reduction in thyroidal T4 content. (**C**) Distribution of allelic variants as determined by Illumina HiSeq analysis of individual *tshr* crispants revealed high mutagenesis efficiency in hypothyroid larvae presenting a hypoplastic thyroid. The percentage of WT alleles (no variant call), in-frame indels, or frameshift indels is shown for *N* = 4–6 larvae per phenotypic category (median values with interquartile range).

Live monitoring of *tshr* crispants at 55 and 80 hpf did not reveal gross deviations from normal thyroid development (Fig. 5 and Supplementary Fig. 5). By 6 dpf, however, a high proportion of *tshr* crispants displayed strongly reduced EGFP reporter expression and thyroids of *tshr* crispants had an overall atrophic appearance reminiscent of the thyroid atrophy/hypoplasia observed in T4-treated larvae (compare Figs 2D and 5A). *tshr* crispants presenting with thyroid atrophy/hypoplasia also showed reduced T4 immunofluorescence indicating a hypofunctional state (Fig. 5B and Supplementary Fig. 5).

Targeting exon 4 and exon 10 of the *tshr* gene resulted in essentially the same thyroid phenotype but its penetrance (Table 1) as well as mutagenesis efficiencies (Fig. 5 and Supplementary Fig. 6) were higher in experiments targeting exon 4 of the *tshr* gene. Total numbers of HiSeq-derived sequences, mapped reads and allele frequencies for *tshr* crispants are provided as Supplementary Information 2. Illumina sequencing of *tshr* crispants showed that frameshift indels account for the majority of sequence variants identified in phenotypic larvae (Fig. 5C, Supplementary Fig. 6 and Supplementary Information 2-NGS Data).

For *tshr* crispants displaying a hypoplastic/hypofunctional thyroid phenotype, the frequency of frameshift indels among mapped sequences ranged between 45.2% (20,996/46,432 mapped reads) and 98.5% (50,626/51,405 mapped reads). All of the observed frameshift indels detected in the vicinity of the gRNA target site in exon 4 introduce premature stop codons and are expected to result in truncated, non-functional tshr variants lacking all 7 transmembranes domains. Collectively, the hypoplastic/hypofunctional phenotypes recovered in *tshr* crispants are very consistent with observations previously reported for murine and zebrafish models with impaired TSHR signalling^33,43^.

**Figure 6:**
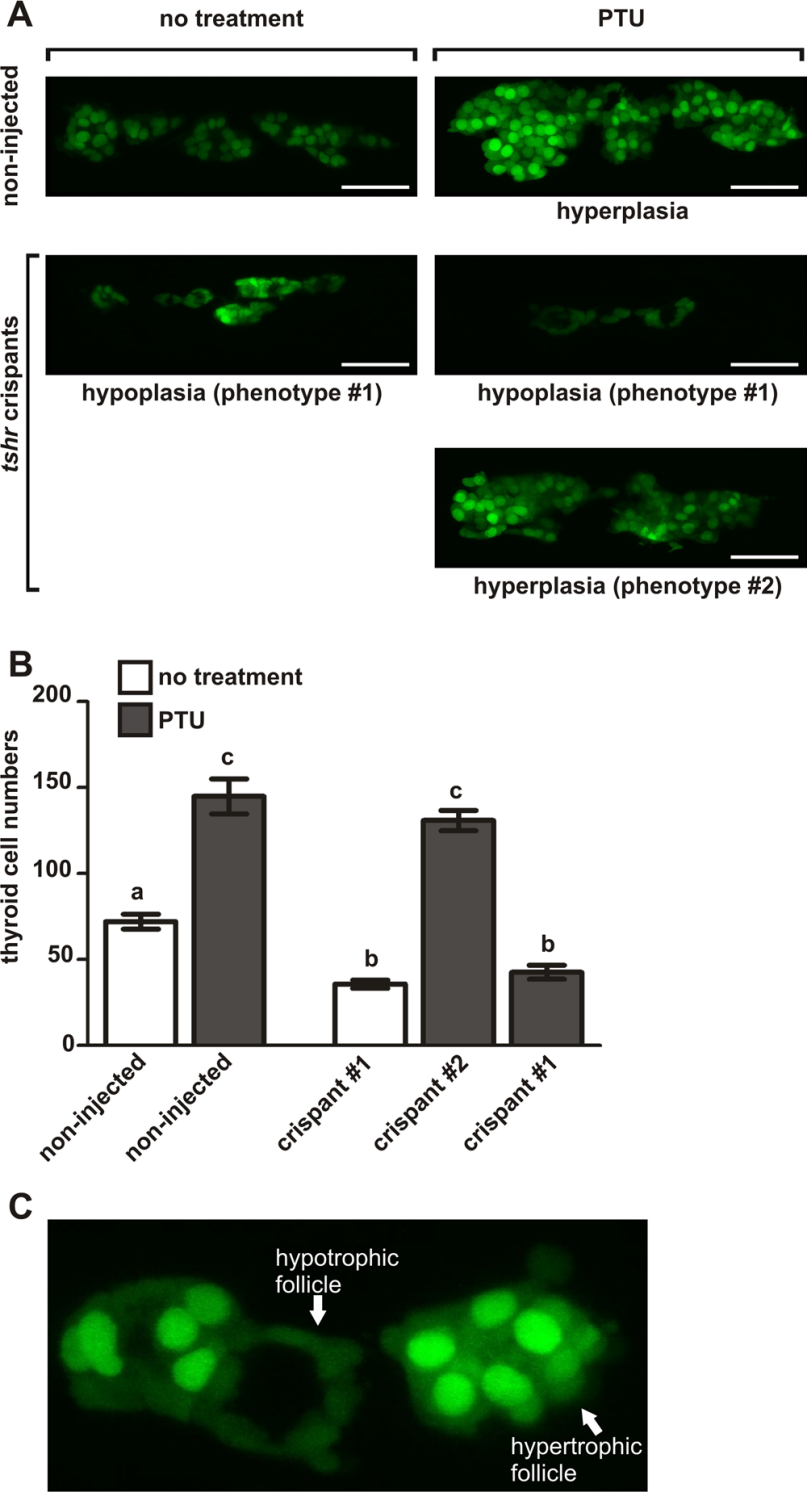
tshr crispants are resistant to PTU-induced thyroid hyperplasia. (**A**) Confocal live imaging of *Tg*(*tg:nlsEGFP*) zebrafish at 6 dpf. Three-dimensional reconstructions of confocal images of the thyroid region (ventral views, anterior to the left, scale bars: 40 µm) are shown for *tshr* crispants and non-injected siblings raised in the presence or absence of 30 mg/L phenylthiourea (PTU). Non-injected control larvae developed severe thyroid hyperplasia in response to PTU treatment. In contrast, only about 50% of the PTU-treated *tshr* crispants displayed a similar thyroid enlargement (phenotype #2). The remaining *tshr* crispants showed an almost complete resistance to PTU-induced thyroid enlargement and instead presented with a hypoplastic/atrophic thyroid phenotype (phenotype #1) similar to that seen in *tshr* crispants raised in the absence of PTU. (**B**) Quantification of thyroid follicular cell number in the different phenotypic groups at 6 dpf. Means ± S.E.M. are shown (N = 12–16). Different letters indicate significant differences between groups (*P* < 0.05, Tukey’s multiple comparison test). (**C**) Confocal live imaging identified mosaicism in cellular responses to PTU treatment in several *tshr* crispants. Note the atrophic characteristics of the follicle in the middle (low reporter expression, flat epithelium) compared to the marked hypertrophy/hyperplasia of the two neighbouring follicles.

Given the high mutagenic efficiency of the gRNA targeting exon 4 of the *tshr* gene and the high penetrance of the associated thyroid hypoplasia phenotype, we next addressed the question of whether somatic mutants provide a valuable model to perform additional functional experiments. For this purpose, we treated *tshr* crispants and non-injected siblings with PTU (30 mg/L) from 52 hpf throughout 6 dpf and assessed all larvae at 6 dpf for the presence of a goitrous thyroid enlargement (Fig. 6). Non-injected larvae showed a very homogenous response to PTU treatment in that thyroids from all fish analysed displayed a distinct thyroid enlargement and strongly increased reporter expression compared to non-injected larvae raised in the absence of PTU (Fig. 6 A). In contrast, PTU treatment failed to evoke a goitrous thyroid response in 77 out of 150 *tshr* crispants (51.3%) analysed in this experiment. The proportion of *tshr* crispants not responding to PTU-induced TSH overstimulation compares well with the proportion of *tshr* crispants displaying thyroid hypoplasia (54.5%) in the initial mutagenesis assay (Table 1). Confocal live imaging of thyroids and quantification of TFC number in larvae from the different phenotypic groups confirmed that hypoplastic *tshr* crispants are almost completely resistant to the trophic effects of TSH stimulation (Fig. 6 A,B). Overall, these observations strengthen the evidence that the hypoplastic/hypofunctional thyroid phenotype in *tshr* crispants is due to severely impaired TSHR signalling. Interestingly, confocal microscopy aided in the identification of several *tshr* crispants with apparent mosaic responses to PTU treatment (see Fig. 6 C) as evident from the heterogenous follicular morphologies (hyperplastic *versus* atrophic). Despite the presence of such mosaicism at the tissue level, the robust tissue responses observed in somatic mutants in our experiments support the notion that somatic mutants provide a valuable experimental system to address first hypotheses regarding the putative role of target genes in classical thyroidal signalling pathways.

### Germline Inheritance of Mutant Alleles and Thyroid Phenotypes in Germline Mutants

**Figure 7:**
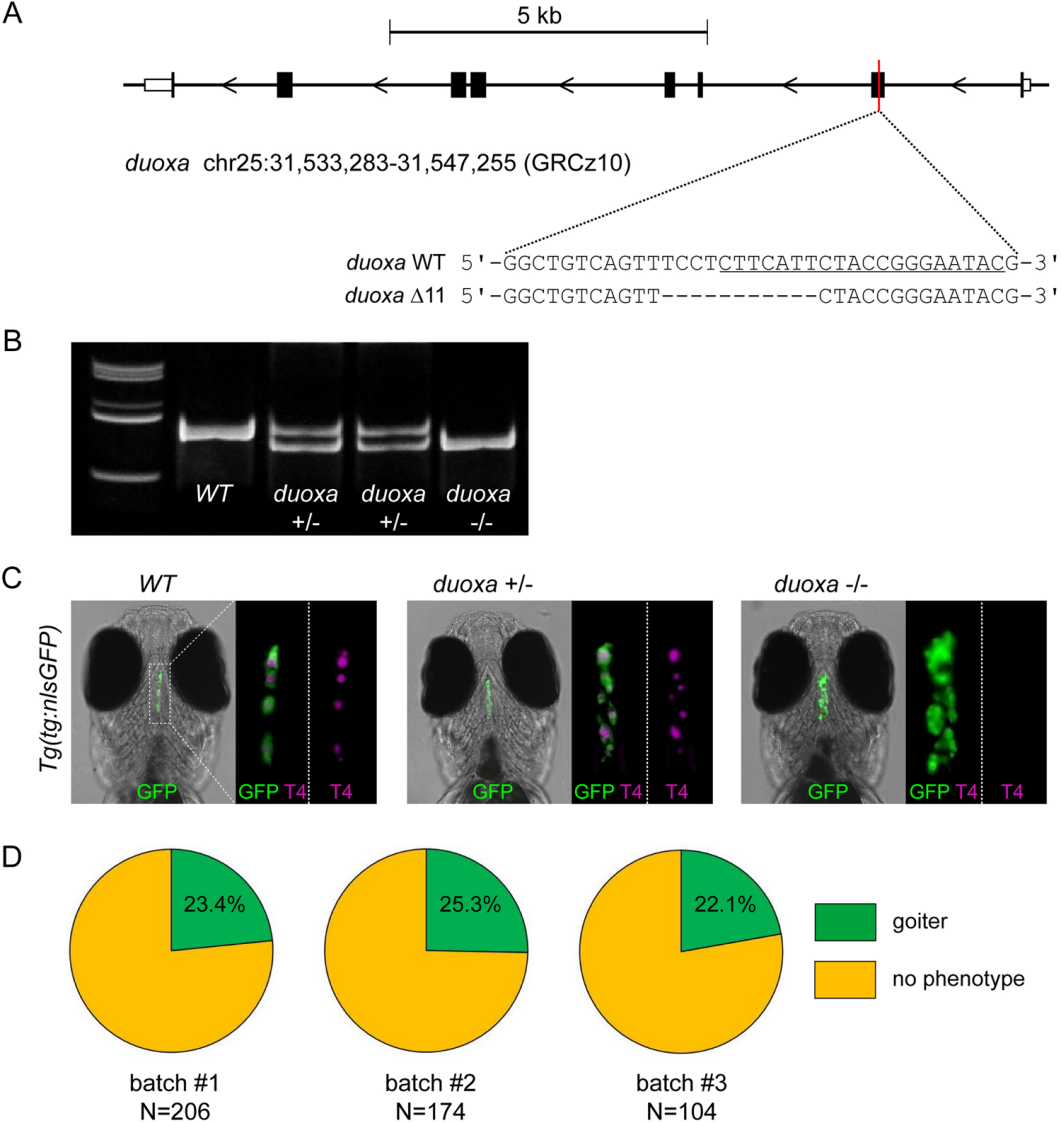
duoxa germline mutants develop goitrous thyroid phenotypes. (**A**) Zebrafish *duoxa* genomic locus on chromosome 25 with sequences for the wild-type (WT) allele and a mutant allele (*duoxa* Δ11) containing a 11 bp deletion in exon 2. The sgRNA target site is underlined in the WT sequence. (**B**) PCR analysis of genomic DNA allows for sensitive detection of WT and *duoxa* Δ11 mutant alleles in individual fish (F3 generation). Polyacrylamide gel electrophoresis of PCR amplicons of WT, heterozygous and homozygous *duoxa* Δ11 carriers. Full-length gel is shown in Supplementary Fig. 7 from which lanes 1, 2, 3, 4 and 6 are shown in the cropped gel image. (**C**) Thyroid phenotyping of *duoxa* Δ11 mutant fish maintained on a *Tg*(*tg:nlsEGFP*) background. Immunofluorescence staining (GFP and T4) of 6 dpf larvae (ventral view, anterior to the top, scale bars: 20 µm) showed goitrous thyroid enlargement and absence of detectable T4 staining in all homozygous *duoxa* Δ11 fish (*N* = 30). Larvae with a normal-looking thyroid morphology (*N* = 30) were genotyped as either WT or heterozygous carriers of the *duoxa* Δ11 allele. For each larvae shown, 3.5-fold magnified views of the thyroid region are displayed (merge of GFP/T4 and T4 only). (**D**) Proportion of larvae with goitrous thyroid phenotype as detected in the progeny of three independent inbreeding experiments with heterozygous *duoxa* Δ11 fish.

To assess germline transmission of mutant alleles and associated thyroid phenotypes, we raised F0 fish from *duoxa* sgRNA injection experiments to adulthood, inbred F0 founders (without prior testing of germline transmission of mutations) and analysed the F1 progeny for thyroid phenotypes. F0 inbreeding experiments consistently confirmed the presence of goitrous thyroid phenotypes in F1 larvae of many crosses, yet with a variable and non-Mendelian distribution. To generate *duoxa* germline mutants with defined genetic lesions, we next outcrossed F0 founders to WT fish, raised their F1 progeny to adulthood and screened adult F1 fish for genome modifications at the *duoxa* locus by Sanger sequencing. We identified several *duoxa* mutant alleles in F1 fish, including deletions and insertions at the target site, and selected a mutant allele harboring a 11 bp deletion (*duoxa* Δ11) in exon 2 site for further analyses (Fig. 7A). The frame-shift *duoxa* Δ11 allele is predicted to encode a truncated duoxa protein of only 55 amino acids (instead of 329 amino acids for the WT protein) including 18 incorrect amino acids at the C-terminus. The deletion allowed for rapid genotyping by PCR (Fig. 7B). Matings of heterozygous *duoxa* Δ11 carriers generated progeny with 22.1 to 25.3% larvae displaying goitrous thyroid enlargement whereas all remaining larvae had normal-looking thyroid morphology (Fig. 7C,D). In total, we assessed 484 fish for thyroid phenotypes. From a total of 115 fish displaying goitrous thyroids, we genotyped 50 fish and found all of them (N = 50/50) to be homozygous carriers of the *duoxa* Δ11 allele. In contrast, genotyping of 85 out of a total of 369 fish displaying normal thyroid development revealed that these fish were either WT (N = 36/85) or heterozygous carriers of the *duoxa* Δ11 allele (N = 49/85). Together, these data corroborate Mendelian inheritance of the dyshormonogenic goiter phenotype and confirm that the thyroid phenotype is genetically linked to the *duoxa* Δ11 allele. Homozygous *duoxa* Δ11 larvae uniformly presented goitrous thyroids with undetectable colloidal T4 by WIF (Fig. 7C) indicating that the *duoxa* Δ11 allele is supposedly a null or severe hypomorphic allele. Gross morphological development of homozygous mutants was similar to WT or heterozygous carriers of the *duoxa* Δ11 allele (Supplementary Fig. 10B).

### Identification of appropriate controls for somatic mutagenesis assays

The analysis of phenotypic traits directly in somatic mutants is a rapidly evolving approach in order to cost- and time-efficiently screen larger panels of candidate genes for their putative functional role in a given biological process^25–27^. Although there is, to date, no clear consensus on the minimally required control experiments validating individual injection experiments, negative and positive injection controls can provide valuable quality control measures. While injection of Cas9 protein together with the *duoxa* sgRNA used in this study would constitute a valuable positive control group presenting goitrous thyroids, we performed additional experiments to identify an appropriate negative control group for our thyroid mutagenesis assay. Specifically, we aimed at the identification of a sgRNA with high mutational activity that does not cause phenotypic alterations in the thyroid.

From previous experiments, we identified *adamtsl2* (ZDB-GENE-070705-558) as a gene with high expression in the developing zebrafish thyroid (Supplementary Fig. 8A) yet no discernible thyroid phenotype when targeted by TALEN-mediated mutagenesis. Based on this information, we designed two specific sgRNAs to target sequences in exon 1 (sgRNA-ex1) and exon 13 (sgRNA-ex13) of the *adamtsl2* gene (Supplementary Fig. 8B) and screened *adamtsl2* crispants according to our thyroid phenotyping protocol.

Live imaging of *adamtsl2* crispants generated with sgRNA-ex1 or sgRNA-ex13 did not reveal deviations from normal thyroid morphogenesis (Supplementary Fig. 8C) and analyses of colloidal T4 content showed no evidence of compromised thyroid function in *adamtsl2* crispants (Supplementary Fig. 8C). These negative findings have been reproduced by injection trials with different batches of embryos (N = 3 for sgRNA-ex1, N = 7 for sgRNA-ex13). Using confocal microscopy, we also examined if injection of sgRNA-ex13 altered TFC numbers in *Tg(tg:nlsEGFP)* larvae but observed no significant differences between mean TFC numbers of control larvae (62.8 ± 4.5, *N* = 9) and *adamtsl2* crispants (60.3 ± 5.7, *N* = 9).

Polyacrylamide gel electrophoresis for PCR-amplified genomic regions spanning the target sites in exon 1 and exon 13 confirmed a high mutagenic activity of both sgRNAs (Supplementary Fig. 8D,E). The target site for sgRNA-ex1 contains a BclI restriction enzyme recognition sequence (Supplementary Fig. 8B) and RFLP analysis of PCR amplicons showed reduced amounts of BclI-digested PCR products for *adamtsl2* crispants compared to the efficient cleavage of the BclI site observed in non-injected controls (Supplementary Fig. 8D).

Notably, although injection of sgRNA-ex1 was associated with a higher level of toxicity (mortality rates between 13 and 29%) compared to sgRNA-ex13 (mortality rates between 4% and 13%), this elevated toxicity did not cause detectable alterations in thyroid morphogenesis/function. Given that *adamtsl2* crispants also did not display discernible gross developmental defects, our studies show that injection of sgRNA-ex1 or sgRNA-ex13 into a group of embryos fulfils the criteria of a valuable negative control group for individual injection experiments.

## Discussion

In this study, we developed a reverse genetic screening strategy to identify genes involved in thyroid morphogenesis and thyroid function. Our approach capitalizes on the high mutagenic efficiency and sequence-specificity of the CRISPR/Cas system, the possibility to monitor the evolution of organ abnormalities in transgenic zebrafish and the sensitive analysis of mutagenesis efficiency by Illumina HiSeq sequencing.

Injection of active Cas9/sgRNA ribonucleoprotein complex into zebrafish one-cell stage embryos reportedly permits bi-allelic invalidation of target genes directly in injected F0 fish and allows to recapitulate loss-of-function phenotypes^25–27^. By targeting genes with known function in early thyroid morphogenesis (*pax2a*, *nkx2.4b*)^37,38^, functional thyroid maturation (*tshr*)^31,43^ and TH synthesis (*duox*, *duoxa*)^40^, we demonstrate in this study that this capacity of the CRISPR/Cas mutagenesis system can be exploited to robustly recover distinct thyroid phenotypes in somatic mutants. Moreover, the rapid development of zebrafish allowed us to limit our phenotypic analyses to the first few days of development permitting a cost- and time-efficient screening strategy.

In its current version, the mutagenesis assay is designed to comprehensively cover phenotypic alterations that might arise from defects in early thyroid morphogenesis (thyroid cell specification, thyroid anlage formation, thyroid budding), early TSH-independent growth of the thyroid primordium, TSH-dependent functional maturation and TH synthesis. To sensitively detect thyroid phenotypes in a rapid and efficient manner, we developed a new transgenic thyroid reporter line permitting a non-invasive monitoring of thyroid development from 55 hpf to 6 dpf using simple fluorescence microscopy equipment. Particularly advantageous properties of the *Tg(tg:nlsEGFP)* line include an overall strong signal intensity facilitating the use of stereomicroscopes for phenotypic analyses and a nuclear-located reporter signal permitting rapid analyses of thyroid cell numbers and changes in cell morphology by confocal live imaging.

Consistent with the functional roles of the genes targeted in this study, monitoring of thyroid reporter fish allowed us to recover severe dysgenesis phenotypes as early as 55 hpf while dyshormonogenesis phenotypes evolved only after the onset of endogenous TH synthesis (around 80 hpf) and became manifest until 6 dpf. Thus, repeated phenotypic analyses over the course of the first six days of development provided first mechanistic information as to the possible functional roles of the genes under study. Accordingly, this knowledge, once fostered with data for additional genes will then allow to customize the observation periods depending on the primary purpose of the screening (i.e. early development *versus* functional maturation).

Results from our experiments highlight specifically the power of CRISPR/Cas-mediated mutagenesis to detect phenotypes arising from defects during late thyroid organogenesis. Zebrafish models based on antisense morpholino injections are generally limited in their utility to study late acting genes because of the transient nature of the gene knock-down accomplished by these reagents. With the CRISPR/Cas system, in contrast, we efficiently recovered thyroid phenotypes related to disrupted TSH signalling or inhibition of TH synthesis although alterations in thyroid morphology and reporter expression required 4 to 6 days to become detectable. We included the T4 immunostaining assay of 6 dpf larvae as an integrated endpoint measurement to sensitively detect deficiencies in TH synthesis^36^. Interestingly, we observed that monitoring of thyroid morphology and reporter expression provided a similar sensitivity for the detection of hypofunctional conditions compared to direct T4 immunostaining.

In the case of genetic defects resulting in impaired TH synthesis (*duox*, *duoxa*), we noted that thyroid tissue of zebrafish larvae rapidly responds to hypothyroid conditions by developing large hyperplastic goiters (due a sustained TSH overstimulation). From these data, we conclude that zebrafish provides an excellent model system to recapitulate the development of dyshormonogenic goiters. On the other hand, the atrophic hypofunctional thyroids observed in *tshr* crispants corroborate the capacity of our phenotyping strategy to detect conditions of diminished TSH signalling. Interestingly, the late onset of a hypoplastic thyroid phenotype in zebrafish *tshr* crispants closely replicates the sequence of thyroid anomalies reported for murine *Tshr* knock-out models^43^. In summary, the spectrum of very specific thyroid phenotypes recovered in somatic crispant models underscores the efficiency of the selected mutagenesis-phenotyping approach to comprehensively cover different aspects of thyroid morphogenesis and functional maturation. Results from our studies also corroborate the conservation of molecular mechanisms regulating thyroid organogenesis in zebrafish and mammals. Thus, this reverse genetics screening strategy holds great promise as an efficient tool for the identification of new genes involved in thyroid development and thyroid function.

Given the streamlined design of sgRNA synthesis, microinjection of active Cas9/sgRNA complexes, live monitoring of transgenic reporter fish and final T4 immunostaining of fixed specimens, our protocol is amenable for higher throughput analyses of larger panels of candidate genes. One appealing approach to screen a larger number of genes would be by multiplexing sgRNAs targeting different genes into a single injection as recently described^26,27^.

In the current study, between 40 and 85% of crispant fish displayed readily detectable thyroid phenotypes in the individual injection experiments. Thus, despite the observation of mosaicism of cellular responses within individual thyroids and the detection of complex genetic mosaicism in all injected fish analysed, the overall robust endpoint responses observed in this study lend further support to the concept that a phenotypic screening of somatic mosaic mutants represents a valuable approach to identify novel genes involved in tissue and organ development^27^.

The analysis of phenotypic traits directly in somatic mutants is a rapidly evolving approach gaining increased popularity but the interpretation of phenotypes is complicated by the fact that somatic mosaicism prevents the use of classical genetic linkage to confirm phenotype/genotype correlations. Therefore, additional quality measures are necessary to validate phenotypic findings in F0 mutagenesis assays although there is to date no established consensus on the minimally required controls validating individual injection experiments. To avoid false negative results due to inactive editing reagents (e.g., Cas9 protein with impaired activity), we recommend to use a positive injection control that would generate a predictable percentage of a specific thyroid phenotype. Based on our results, any of the sgRNAs used in this study would qualify for such a positive control.

False positive findings, on the other hand, might occur due to various reasons including possible genome editing activity at off-target sites, unspecific toxicities of the editing reagents or artefacts occurring during experimental manipulation (e.g., microinjection procedure). So far, results from an increasing number of *in vivo* mutagenesis studies revealed little if any evidence for genome editing at off-target sites as a relevant confounding factor in experiments applying CRISPR/Cas-technology^22,25^. However, as we did not attempt to specifically analyse loci bearing some level of sequence similarity to our target sequences, we cannot rule out the existence of off-target activities for any of the Cas9/sgRNA ribonucleoprotein complexes that were injected in the course of the current studies. Give the intended use of our reverse genetics strategy as a first screening tool to identify candidate genes, we rather favour another option to rule out off-target effects as the cause for the phenotypic alterations detectable in F0 fish, that is by performing classical segregation analysis of phenotypic traits in the offspring of stable mutants as we have shown for the dyshormonogenic goiter phenotype in the *duoxa* Δ11 germline mutant line. Particularly when targeting genes with hitherto unknown function in thyroid development/function, this will be critically important before definitive conclusions are drawn on the role of any given gene.

Nevertheless, it is desirable to implement some quality measures in protocols for each individual injection experiment such as negative injection controls. To date, there is no consensus how such a “mock” control might look like. While injecting Cas9 protein together with a non-targeting sgRNA might be one option to establish a negative injection control, we favoured the use of a sgRNA that would cause editing of a real sequence without causing a thyroid phenotype. With *adamtsl2*, we identified a gene that is expressed in zebrafish thyroid cells but that causes no detectable alterations in thyroid development or functional capacity if targeted by various sgRNAs. Implementing the proposed positive and negative control groups in routine injection protocols will strengthen the validity of the powerful approach of F0 mutagenesis.

One of the most critical elements of any mutagenesis strategy relying on phenotypic analyses of somatic mutants is to carefully control the mutagenesis efficiency of the injected sgRNAs. This is particularly important to rule out false negative results as a result of insufficient bi-allelic genome editing. Several methods are available for the detection, quantification, and characterization of indels in the target region including restriction enzyme assays^26^, T7 endonuclease assays^26^, hetero-duplex mobility assays^44^, high-resolution melt analysis^45^, PCR based methods^46^, classical cloning followed by Sanger-Sequencing^22,25^ as well as deep-sequencing techniques^22,25,27^. For our studies, we identified Illumina HiSeq sequencing of whole body genomic DNA extracted from individual crispants as the method of choice. In combination with CrispRVariants software, we found that this approach provided critically relevant information to confirm that mutagenesis efficiencies of individual sgRNAs are sufficiently high to achieve bi-allelic invalidation of the target gene and to obtain additional information about the frequency and distribution of in-frame and frameshift indels.

Thyroid cells represent a very small cell population in zebrafish larvae and unless costly and laborious techniques based on cell sorting and single cell sequencing are used, it will remain impossible to reconstruct the allelic composition of thyroid cells in F0 crispants. We therefore stress that data generated by our sequencing of genomic DNA extracted from whole larvae do by no means reflect the allelic composition of thyroid cells. When confronted with the difficulties inherent to the interpretation of possible phenotype-genotype relationships in genetically mosaic samples, we found it worthy to perform our sequencing analyses for individual larvae rather than for pools of larvae. Given the limited accessibility to target cells populations, such as thyroid cells, for direct genotyping analyses, we consider it critical to derive genetic information at the next higher level of organisation that is accessible for analysis (i.e, individual fish) in order to generate more knowledge and advance our understanding how patterns of allelic composition relate to phenotypic differences. At this early phase of F0 mutagenesis assay development, we also think that pooling of several crispants prior to sequencing might bear the danger of diluting or masking information that could be relevant for the interpretation of the assay outcome.

In our study, the information-rich data of Illumina HiSeq sequencing allowed for some interesting observations. First, across the various genes targeted in our study, we found that fish presenting the most severe thyroid phenotypes have very high frequencies of mutant alleles (between 80 and 100% of all sequence reads) suggesting that bi-allelic invalidation of the target genes was likely achieved in thyroid cells. Fish presenting weaker phenotypes or no detectable phenotype had a much more variable frequency of edited alleles. Although we so far analysed only a limited number of larvae, it seemed that the distribution and frequency of edited alleles detected in whole larvae alone is not necessarily a strong predictor of phenotypic severity of the actual thyroid phenotypes. Whether this reflects the small number of samples analysed or indicate that the predictive value will depend on the gene in question remains elusive so far.

Of particular interest to us was the identification of some outlier fish, that is individual crispant fish lacking a detectable phenotype despite relatively high frequency of frameshift mutations at the target site and *vice versa*. We currently explain these examples by genetic mosaicism within the thyroid cell population. Although genome editing occurs very early during zebrafish development, cells within a given lineage can have distinct allelic compositions^47^ and our confocal imaging confirmed that mosaicism can even be observed within the small zebrafish thyroids. Assuming that the thyroid is composed of progeny of several progenitor cells with diverse patterns of genetic mosaicism, then the presentation of a phenotype might depend on various factors such as relative size of the mosaic patches, potential growth rate advantages of normal, hypomorphic or knock-out allele carriers, and whether the deleterious mutations occurs in a gene acting in a cell-autonomous or non-cell-autonomous fashion. More studies targeting a wider range of genes will be necessary to address these critical questions. The interpretation of phenotypic patterns in mosaic F0 zebrafish mutants is a new and challenging research topic and might benefit a great deal from incorporating knowledge generated in other well studied models of cellular mosaicism. Of particular interest might be a comparison with models of X-linked diseases and the various factors that determine how female mosaicism of X inactivation in mammals can affect phenotypic presentation^48^.

In summary, we describe a new approach to identify genes involved in thyroid development and function based on CRISPR/Cas mutagenesis in zebrafish embryos. Our phenotyping strategy based on live monitoring of organ development in mutagenized reporter fish proved a robust and sensitive means to recapitulate classical thyroid phenotypes. Our results strengthen the concept that somatic mutants provide a valuable model system for phenotypic screens. Pending the availability of suitable reporter models, the general screening strategy can be adapted for studies on other endoderm-derived organs.

## Materials and Methods

### Animal husbandry

Zebrafish embryos were collected by natural spawning and embryos and larvae were raised at 28.5 °C under standard conditions^49^ and staged according to hours (hpf) or days post fertilization (dpf) as described^50^. For screening and live imaging, embryos and larvae were anesthetized in 0.02% tricaine. The following zebrafish lines were used in this study: wild-type AB strain (WT), pigmentless *Casper* strain^51^, *Tg*(*tg:mCherry)*^*ulb1*^^30^ and *Tg*(*tg:nlsEGFP)*^*ulb4*^ (this study). All zebrafish husbandry and all experiments were performed under standard conditions in accordance with institutional (Université Libre de Bruxelles-ULB) and national ethical and animal welfare guidelines and regulation. All experimental procedures were approved by the ethical committee for animal welfare (CEBEA) from the Université Libre de Bruxelles (protocol 578 N).

### Generation of *Tg(*Tol2*tg*:nlsEGFP*)* thyroid reporter fish

The Gateway cloning technology-based Tol2kit^52^ was used to generate a reporter construct in which expression of a nuclear-localized EGFP variant is driven by a fragment of the zebrafish *tg* gene promotor previously shown to permit thyroid-specific transgene expression^30^. The *tg* promotor sequence^30^ was sub-cloned into the multi-cloning site (*Xho*I and *BamH*I) of p5E-MCS (Tol2kit vector 228) to generate a p5E-*tg* vector. Next, a Tol2*tg*:nlsEGFP vector was assembled by recombining p5E-*tg*, pME-nlsEGFP (Tol2kit vector 385), and the destination vector pTol2DestR4R2pA in a LR reaction^53^. The destination vector contained flanking Tol2 ends so that the final Tol2*tg*:nlsEGFP vector could serve as the transposon donor vector in subsequent Tol2 transgenesis. Capped mRNA encoding for *transposase* was generated by *in vitro* transcription of *Not*I-linearized pCS-zT2TP plasmid^54^ using mMessage mMachine SP6 kit (Ambion).

Transgenic embryos were produced by co-injection of Tol2*tg:*nlsEGFP vector (25 ng/µL) and Tol2 transposase mRNA (35 ng/µL) in one-cell stage WT embryos. Injected embryos were screened at 55 hpf for EGFP expression in thyroid cells and F0 fish with thyroid-specific EGFP expression were raised to adulthood. F0 founders with germline transmission of the transgene were crossed with WT fish to generate stable transgenic *Tg(*Tol2*tg*:nlsEGFP*)* lines. We initially maintained six founder lines over two generations and selected one line with robust reporter expression in all thyroid cells for the experiments reported in this study. This *Tg*(*tg:nlsEGFP)*^*ulb4*^ line is maintained in both WT and *Casper* backgrounds.

### Phenylthiourea and thyroxine treatment of developing zebrafish

Zebrafish *Tg*(*tg:nlsEGFP*) embryos and larvae were treated with phenylthiourea (PTU; Sigma) or thyroxine (T4; Sigma) from 52 hpf onwards in order to induce hypo- and hyperthyroid conditions, respectively. Stock solutions of 100 mg/L PTU and 5 mg/L T4 were diluted in embryo medium to make experimental treatment solutions of 30 mg/L PTU and 5.0 µg/L T4. A control group was maintained in embryo medium. Embryos and larvae were treated in 90 mm petri dishes containing 25 mL of treatment solution and solutions were renewed every other day.

### sgRNA design and synthesis

The Sequence Scan for CRISPR software (available at http://crispr.dfci.harvard.edu/SSC/) was used to identify single guide RNA (sgRNA) sequences with high on-target activity^55^. The CRISPR design web tool available at http://crispr.mit.edu was used to predict potential off-target sites^56^. sgRNAs with highest on-target activity and lowest predicted off-target score were selected for the experiments.

DNA templates for sgRNA synthesis were produced using the PCR-based short-oligo method as described^45^. Briefly, DNA templates were generated by annealing a scaffold oligonucleotide (sequence: AAAGCACCGACTCGGTGCCACTTTTTCAAGTTGATAACGGACTAGCCT TATTTTAACTTGCTATTTCTAGCTCTAAAAC) with gene-specific oligonucleotides containing the SP6 promoter sequence (GCGATTTAGGTGACACTATA) followed by a 20 base target sequence without the PAM (see Supplementary Table 1) and a sequence complementary to the scaffold oligo (GTTTTAGAGCTAGAAATAG). PCR amplification was performed with Taq PCR Core Kit (QIAGEN) using 20 nM scaffold oligo, 20 nM gene-specific oligo, and 260 nM of each universal flanking primer (forward: GCGATTTAGGTGACACTATA, reverse: AAAGCACCGACTCGGTGCCAC). PCR products were purified by MinElute Reaction Cleanup kit (QIAGEN) followed by phenol-chloroform-isoamylalcohol extraction.

sgRNAs were synthesized *in vitro* from purified PCR products by using SP6 RNA-polymerase (NEB). 20 µL reactions contained 1 µg DNA template, 1x reaction buffer, 2.5 mM of each rNTP (Roche), 40 U RNase Inhibitor (Thermo Fisher Scientific), and 6 U Large Fragment of DNA Polymerase I (Thermo Fisher Scientific). After pre-incubation for 15 min at room temperature, 20 U SP6 RNA-polymerase were added and reactions were run over night at 40 °C. After treatment with 3 U DNase I (AmpGrade; Thermo Fisher Scientific), sgRNAs were purified using High Pure PCR Cleanup Micro Kit (Roche). Concentration of sgRNAs was measured by Nanodrop (Thermo Fisher Scientific) and integrity was checked by gel electrophoresis. Aliquots of sgRNA solutions were stored at − 80 °C until use.

### Cas9/sgRNA injections

Cas9 protein (PNA Bio; 100 ng/µl final concentration) and sgRNA (80 ng/µL final concentration) were mixed in 200 mM KCl solution containing 0.15% phenol red (Sigma) and incubated for 5 min at room temperature. Approximately 3 nL of active sgRNA-Cas9 ribonucleoprotein complex were injected per one-cell stage *Tg*(*tg:nlsEGFP*) embryo. For each sgRNA, at least three independent injection experiments were performed with spawns from different founder fish.

### Phenotypic analysis of crispant fish

Injected embryos (crispants) and non-injected siblings (controls) were raised in 90 mm petri dishes (approximately 50 embryos per dish) containing 25 mL of embryo medium. Between 24–26 hpf, all crispant and control embryos were dechorionated by a gentle 20 min treatment with pronase (0.6 mg/mL). Thyroid development was monitored in live zebrafish at 55 hpf, 80 hpf and 6 dpf by visual inspection using a M165 FC fluorescence stereomicroscope (Leica). For the duration of the phenotypic analysis, embryos and larvae were anesthetized in 0.02% tricaine. All control and crispant fish were inspected for gross developmental defects, deviations in size, shape or location of the thyroid, and for the overall intensity of the fluorescent reporter signal.

For documentation of thyroid phenotypes, embryos and larvae were immobilized in 1% low-melting point agarose (Lonza) containing 0.016% tricaine and imaged on Fluoro-Dish glass bottom dishes (WorldPrecisition Instruments). Images of the ventral pharyngeal region were acquired using a DMI600B epifluorescence microscope equipped with a DFC365FX camera and LAS AF Lite software (Leica). If indicated, confocal live imaging was performed with a LSM 510 confocal microscope (Zeiss) using Zen 2010 D software (Zeiss).

### Quantitative Analysis of Fluorescence Signal Intensities

For quantification of PTU and T4 treatment effects on fluorescence signal intensities^36^, images of the thyroid region were acquired for live *Tg*(*tg:nlsEGFP*) fish at 6 dpf using a DMI600B epifluorescence microscope. Using LAS AF imaging software, pixel sum values were obtained for fluorescent thyroid tissue (region of interest 1, ROI-1) and adjacent non-fluorescent regions (ROI-2, background). For each fish analysed, the intensity of ROI-1 was corrected by subtracting the intensity of ROI-2. Values of background-corrected fluorescence signal intensities from individual fish were used in comparisons between treatment groups and controls.

### Whole-mount *in situ* hybridization (WISH)

Embryos and larvae were fixed in phosphate-buffered saline (PBS) containing 4% paraformaldehyde (PFA, pH 7.3) over night at 4 °C, washed in PBS containing 0.1% Tween 20, gradually transferred to 100% methanol, and stored at −20 °C until use. DNA templates for synthesis of *adamtsl2*, *nkx2.4b*, *slc5a5*, *tg* and *tshb* riboprobes were generated by PCR (see Supplementary Table 1 for primer sequences) as described^33^. WISH experiments were performed essentially as described^33^ using riboprobes labelled with digoxigenin (DIG), anti-DIG antibody conjugated to alkaline phosphatase (1:6000, Roche) and BM Purple (Roche) as substrate for alkaline phosphatase stainings. Stained embryos were postfixed in 4% PFA (Sigma) and embedded in 90% glycerol for whole-mount imaging using an Axiocam digital camera mounted on an Axioplan 2 microscope (Zeiss).

### Whole-mount immunofluorescence (WIF)

Embryos and larvae were fixed in 4% PFA (pH 7.3) over night at 4 °C and stored in PBS containing 0.1% Tween 20 at 4 °C until use. WIF experiments were performed essentially as described^33^ using the following antibodies: rabbit anti-T4 polyclonal antibody (1:2000; MP Biochemicals), chicken anti-GFP polyclonal antibody (1:1000; Abcam), rabbit anti-pax2a polyclonal antibody (1:250; GeneTex), cy3-conjugated donkey anti-rabbit IgG antibody (1:250; Jackson ImmunoResearch), and Alexa Fluor 488-conjugated goat anti-chicken IgG antibody (1:250; Invitrogen). Stained specimens were postfixed in 4% PFA, gradually transferred to 100% glycerol and phenotypically analysed using a M165 FC fluorescence stereomicroscope. Whole mount imaging of stained specimens was performed with a DMI600B epifluorescence microscope equipped with a DFC365FX camera.

### Illumina HiSeq-sequencing

Four to six individual larvae per phenotypic category were selected for genotypic analysis by Illumina HiSeq sequencing. Individual larvae were lysed in 50 mM NaOH at 95 °C for 10 min and the lysate was neutralized with 0.5 M Tris (pH 8.0). Two rounds of PCR were performed to produce barcoded libraries for Illumina sequencing. First, gene specific primers (Supplementary Table 1) containing 5′ extensions for the second PCR step were used to amplify a region spanning the target site (amplicon size between 100 and 250 bp). In the second PCR, amplicons were barcoded using Nextera sequencing primers I5 and I7. For a given sgRNA target, each individual zebrafish received one unique barcode. Practically, the PCR approach was performed by arranging the samples in a matrix with lines corresponding to gene-specific PCR samples (first PCR) of one sgRNA target, and columns receiving the different barcodes (second PCR). Barcoded amplicons were checked on agarose gels, quantified using PicoGreen reagent (Thermo Fisher Scientific), and pooled at equal molar ratios. Paired-end sequencing (125 bp) was performed on pools of amplicons using a HiSeq Sequencer (Illumina). After sequencing, reads were de-multiplexed based on the barcodes and stored as FASTQ files.

Paired reads were merged using PEAR 0.9.10^57^ and subsequently aligned with BWA-MEM^58^ to the zebrafish genome version GRCz10. We used CrispRVariants^59^ for variant counting and data visualization. By applying a 5% frequency cut-off, counting of variants was performed within a region spanning from 5 bp upstream of the sgRNA to 27 bp downstream of the PAM site. A transcript database based on GRCz10 was used (‘make Tx Db from GFF from Genomic Features R package’)^60^ to classify variants as either no-variant, single-nucleotide variant (SNV), frameshift indel, in-frame indel, splice-site variant, or chimeric reads. Total numbers of HiSeq-derived sequences as well as absolute and relative allele frequencies detected in individual larvae are provided as accompanying Supplementary Information (Supplementary Information 2: NGS data).

### Genotyping of germline mutants

To assess germline inheritance of genome modification induced in somatic mutant fish, F0 founders from *duoxa* sgRNA injection experiment were outcrossed to *WT* fish and genomic DNA was extracted from F1 larvae or tailfin clips of adult F1 fish using the protocol described above. DNA from individual fish was used as a template for subsequent PCRs using primers spanning the target site of *duoxa* sgRNA (for primer sequences see Supplementary Table 1). PCR amplicons encompassing the target region were analyzed by Sanger sequencing to detect sequence variants. The same primers were used for PCR amplification of genomic DNA in order to discriminate *WT*, heterozygous and homozygous carriers of the *duoxa* Δ11 allele among the offspring of subsequent inbreeding experiments.

### Heteroduplex mobility assay of somatic mutants

To assess the efficiency of sgRNAs targeting exon 1 and exon 13 of *adamtsl2* to induce site-specific genome modifications, genomic DNA was extracted from individual 6 dpf larvae (F0 crispants and non-injected controls) using the protocol described above. DNA from individual fish was used as a template for subsequent PCRs using primers spanning the target sites of *adamtsl2* sgRNAs (for primer sequences see Supplementary Table 1). PCR amplicons were electrophoresed on 12% polyacrylamide (Bio-rad) gels and analysed for the distribution of homoduplex and heteroduplex bands.

### Restriction fragment length polymorphism analysis

The sgRNA target site in exon 1 of the *adamtsl2* gene contains a Bcl1 restriction enzyme recognition site. To assess the mutagenic activity of sgRNA-ex1, PCR amplicons (expected size of 206 bp) encompassing the target region in exon 1 were digested with Bcl1 (NEB #RO160S) for one hour at 50 °C. Reaction products (expected size of restriction fragments: 111 bp and 95 bp) were loaded on 1.0% agarose and on 12% polyacrylamide gels and analysed for the presence of undigested bands (loss of restriction site).

### Statistical analysis

Cell number and relative fluorescence data were assessed for normal distribution (KS-normality test) and analysed either by unpaired student’s t-test or by one-way ANOVA followed by post hoc Tukey–Kramer’s multiple comparison test. Differences were considered significant at p ≤ 0.05. Statistical analyses were performed using the software package Graph-Pad Prism 5.01.

### Data Availability

All data generated or analysed during this study are included in this manuscript and its Supplementary Information files.

## Electronic supplementary material

Supplementary Information
