# Supplementary material for: A Rapid CRISPR/Cas-based Mutagenesis Assay in Zebrafish for Identification of Genes Involved in Thyroid Morphogenesis and Function
Source: Sci Rep. 2018 Apr 4;8:5647. doi: 10.1038/s41598-018-24036-4 (PMC5884836; doi:10.1038/s41598-018-24036-4)
Supplement: Supplementary file 1 — Supplementary Information [file 41598_2018_24036_MOESM1_ESM.pdf]

Supplementary Information 1: Sup Tables 1-2 and Sup Fig 1-10

A Rapid CRISPR/Cas-based Mutagenesis Assay in Zebrafish for Identification of Genes Involved in Thyroid Morphogenesis and Function

Trubiroha, A.[1,a], Gillotay, P.[1], Giusti, N.[1], Gacquer, D.[1], Libert, F.[1], Lefort, A.[1], Haerlingen, ,B.[1], De Deken, X.[1], Opitz, R.[1,2] and Costagliola, S.[1]

[1] Institute of Interdisciplinary Research in Molecular Human Biology (IRIBHM), Université Libre de Bruxelles, Route de Lennik 808, 1070 Brussels, Belgium

[2] Institute of Experimental Pediatric Endocrinology, Charité Universitätsmedizin Berlin, Augustenburger Platz 1, 13353 Berlin, Germany

A. Trubiroha, P. Gillotay and N. Giusti contributed equally to this work

[a] present address: German Federal Institute for Risk Assessment (BfR), Department Chemicals and Product Safety, Max-Dohrn-Strasse 8-10, 10589 Berlin, Germany

Corresponding authors:

Sabine Costagliola (scostag@ulb.ac.be)

Robert Opitz (robert.opitz@charite.de)

This file contains 2 Tables, 10 Figures and Supplementary NGS data

Supplementary Table 1. gRNA target sequences and primers used for genotyping

Supplementary Table 2. Primers used for PCR-based approach of RNA probe preparation

Supplementary Figure 1. Quantification of fluorescence signal intensity in live transgenic *Tg(tg:nlsEGFP)* zebrafish larvae at 6 dpf.

Supplementary Figure 2. Phenotypic spectrum of *pax2a* and *nkx2.4b* crispants

Supplementary Figure 3. Thyroid anlage specification is not perturbed in *pax2a* crispants

Supplementary Figure 4. Phenotypic spectrum of *duox* and *duoxa* crispants

Supplementary Figure 5. Phenotypic spectrum of *tshr* crispants

Supplementary Figure 6. Illumina HiSeq analyses of *nkx2.4b*, *duoxa* and *tshr* crispants

Supplementary Figure 7: Full length gel for the cropped gel shown in Figure 7.

Supplementary Figure 8. Characterization of *adamtsl2* crispants

Supplementary Figure 9. Full length gels for the cropped gels shown in Suppl. Figure 8

Supplementary Figure 10. Brightfield micrographs of crispants and germline mutant fish

Supplementary NGS data. Allele frequencies of individual F0 crispants as determined from HiSeq sequencing data by CrispRVariants Software

Supplementary Tables 1 – 2

Supplementary Table 1: gRNA target sequences and primers used for genotyping

target	gRNA target sequence (5′-3′)	strand	forward primer (5′-3′)	reverse primer (5′-3′)	product size
adamtsl2 exon 1	GGCTGGAAGTGATCACATAC	+	GCTTCAGACAGTGTCTCTCCTG	GCTTCAGACAGTGTCTCTCCTG	206 bp
adamtsl2 exon 13	AGAGGTCAGATGCTCAGATG	+	CAGCTAAATGTGGTCGCAAG	GGTCCCCACTCGGATGCT	151 bp
duox exon 23	GTCATCTACGCCATCTCCGC	+	ACCGGAGTCAATTTTCCCTTG	ACTTACAGATGCATCTTTCC	210 bp
duoxa exon 2	GTATTCCCGGTAGAATGAAG	+	GCTTTAGTTCTCGCCTGTGG	TCACCGATCTGCCACGTATT	178 bp
nkx2.4b exon 1	GTTCGAGCCGTACCAAGCCG	-	GAGCCATAACGCGTACCACA	GTATCTCGGCTCCGGGTTC	169 bp
nkx2.4b exon 2	AGCACGCGCCGTTTCCTGCG	-	AAAGTCTCCAGGTTTATGGG	CGCTCCAGCTCGTATACCT	152 bp
pax2a exon 2	GGCGGTGTGAACCAGCTAGG	+	TGTGTCTGTGTTGTTGTATCCC	TGGCTGACCCGTAGTTGTCT	167 bp
tshr exon 4	GCTGACATTCATCCACCCAG	+	GGAACACACGAAAGCTGACAT	AACAGTGATTTTGCTCACAGATAC	85 bp
tshr exon 10	GATCATAGGTGTCTCTGAAG	-	GAACCTGGCATCGACTTGAG	GTTTCACCGAAACCCATGTT	128 bp

Supplementary Table 2: Primers used for PCR-based approach of RNA probe preparation

target	GenBank Accession	forward primer (5′-3′)	reverse primer (5′-3′)	probe size
adamtsl2	XM_021476679	AAGAAAACAACAACCGGTAAAGAC	CTGTAAAACACTCACAACCTCCAC	857 bp
nkx2.4b	NM_131589	TTTCTCAGTGAGCGATATTTTGAG	CGTATACAATTCACGTCAAAGAGG	1058 bp
slc5a5	NM_001089391	TTTGTATGGCTTCAAGTTCCTGTA	TGTTTTCTCTGTGAAATGGTGATT	906 bp
tg	NM_001329865	TAATTGTAGTGACGGCCAGTTTTA	TTTTCTCCTTGTAGCTGAAGAGGT	796 bp
tshb	NM_181494	TCCAGACAGACATCCTCATACACT	TGAACCCTATTAAAACCACACCTT	857 bp

Note: A sequence containing the T3 polymerase promoter was added to the 5’ end of each reverse primer sequence in order to obtain a PCR product that could be used to generate an antisense probe during *in vitro* transcription. Therefore, the final design of the reverse primers used in this study was as follows: 5’ GGATCCAATTAACCCTCACTAAAGGGAA(N)24 3’ with N representing the 24 nucleotides of the gene-specific reverse primer. The bases of the T3 polymerase promoter become double-stranded promoter sequence during the PCR reaction.

Supplementary Figures 1 – 10

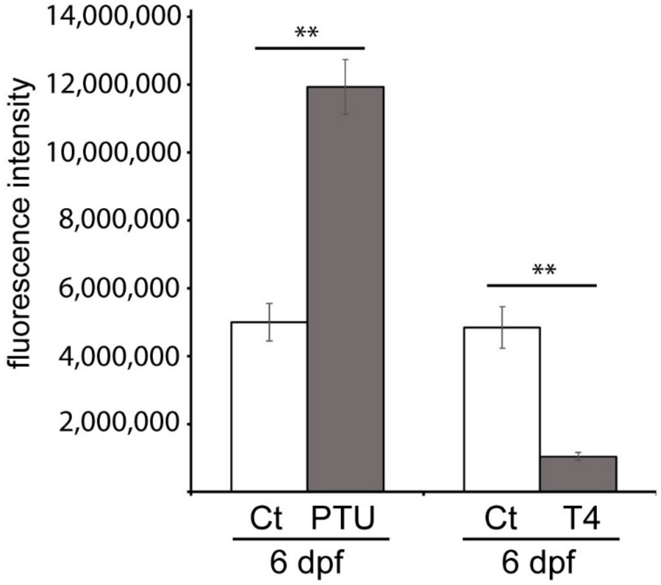

Supplementary Figure 1: Quantification of fluorescence signal intensity in live transgenic *Tg(tg:nlsEGFP)* zebrafish larvae at 6 dpf. Transgenic zebrafish were treated from 52 hpf onwards with 30 mg/L phenylthiourea (PTU) or 5.0 µg/L thyroxine (T4). Fluorescent reporter signal intensities were analysed in untreated controls (Ct) and treated fish by epifluorescence microscopy. Results are shown as means±S.E.M. (N=7-9). Asterisks denote significant differences between treatment means (** $P < 0.01$, Student t-test).

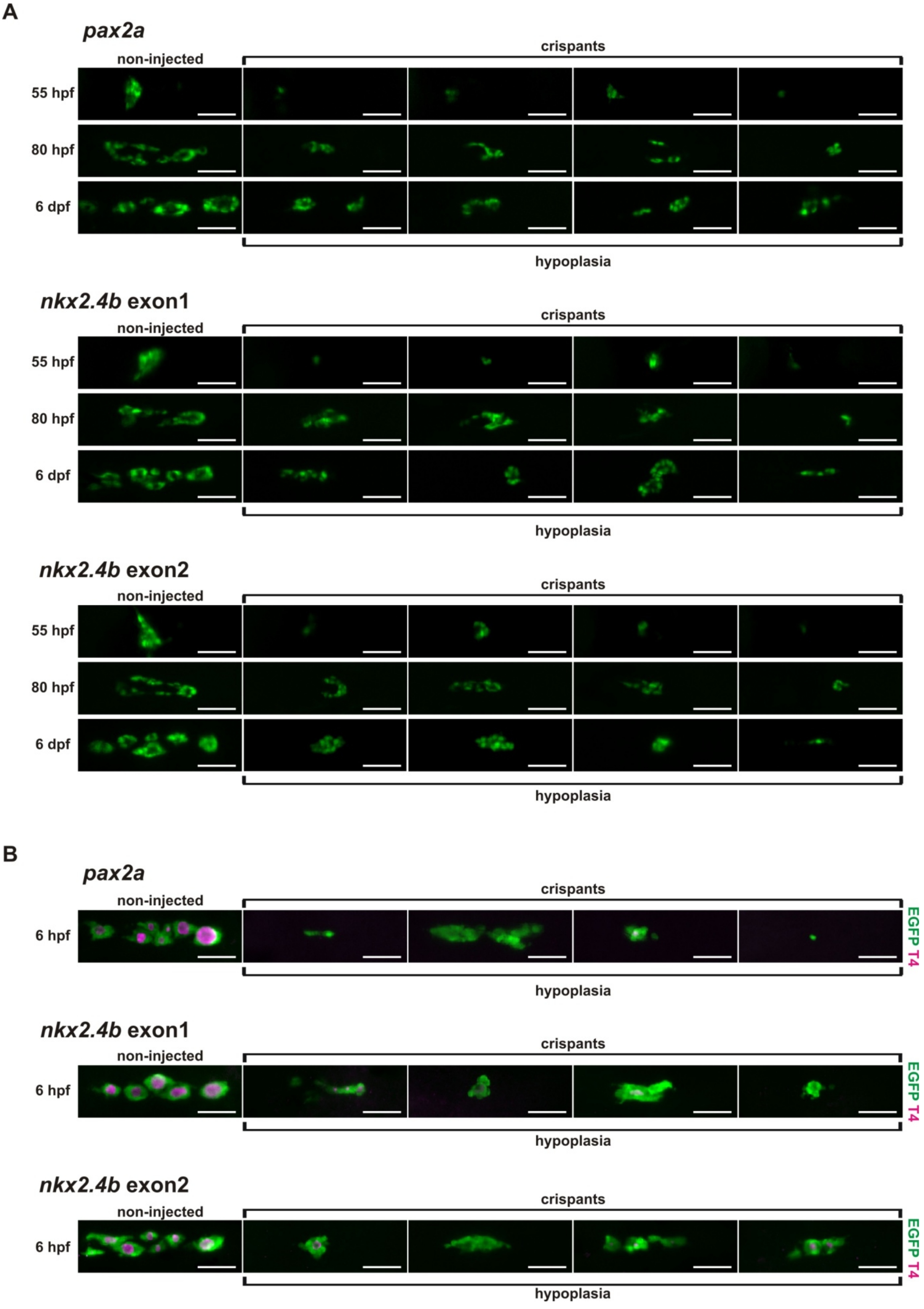

Supplementary Figure 2: Phenotypic spectrum of *pax2a* and *nkx2.4b* crispants. (A) Epifluorescence live imaging of *Tg*(*tg:nlsEGFP*) zebrafish larvae injected with Cas9 protein

and sgRNAs targeting *pax2a* or *nkx2.4b* (exon 1 and exon 2), respectively. Ventral views of the thyroid region (anterior to the right) are shown for non-injected control fish and four different crispants displaying hypoplasia of variable severity. Images were aquired at 55 hpf, 80 hpf, and 6 dpf. (B) Whole-mount immunofluorescence staining of *Tg*(*tg:nlsEGFP*) zebrafish for EGFP and colloidal T4 at 6 dpf. Ventral views of the thyroid region (anterior to the right) are shown for non-injected control fish and four different crispants displaying hypoplasia of variable severity. Scale bars: 50 μM.

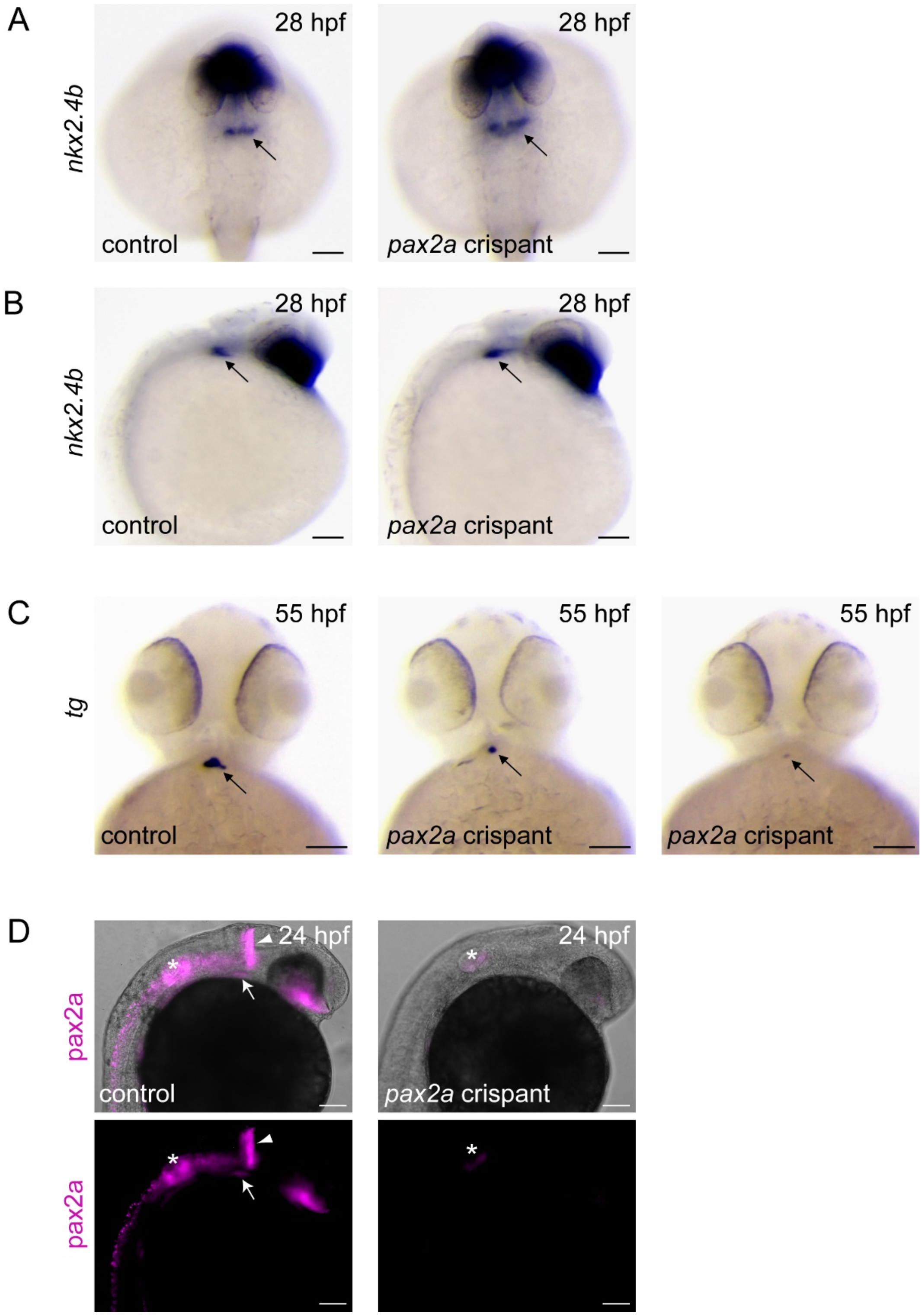

Supplementary Figure 3: Thyroid anlage specification is not perturbed in *pax2a* crispants. (A,B) Whole-mount *in situ* hybridization for the early thyroid marker *nkx2.4b* in non-injected control embryos and *pax2a* crispants. Thyroidal *nkx2.4b* expression (arrow) was not different between experimental groups at 28 hpf. Panel A shows dorsal views of stained specimen (anterior is to the top). Panel B shows lateral views (anterior is to the right). (C) Loss of thyroid marker expression in later stage *pax2a* crispants. Whole-mount *in situ*

hybridization for the thyroid differentiation marker *tg* in non-injected control embryos and *pax2a* crispants at 55 hpf. In contrast to the strong *tg* staining in the compact and slightly ovoid thyroid primordium of control embryos, half of the *pax2a* crispants showed either a complete absence of detectable *tg* staining (data not shown) or presented thyroid primordia of greatly reduced size. Ventral views are shown (anterior is to the top). (D) *pax2a* crispants display strongly reduced immunoreactivity to a pax2a antibody. Whole-mount immunofluorescence staining of pax2a (magenta) was performed on non-injected control embryos and *pax2a* crispants at 24 hpf using a pax2a antibody directed against an epitope located C-terminal to the sgRNA target site. Approximately half of the *pax2a* crispants (53.7%; *N*=79/147) displayed a strongly reduced pax2a immunofluorescence signal. Lateral views are shown (anterior is to the right). Arrowheads: mid-hindbrain boundary; arrows: thyroid anlage; asterisks: otic vesicle. Scale bars: 100 µm.

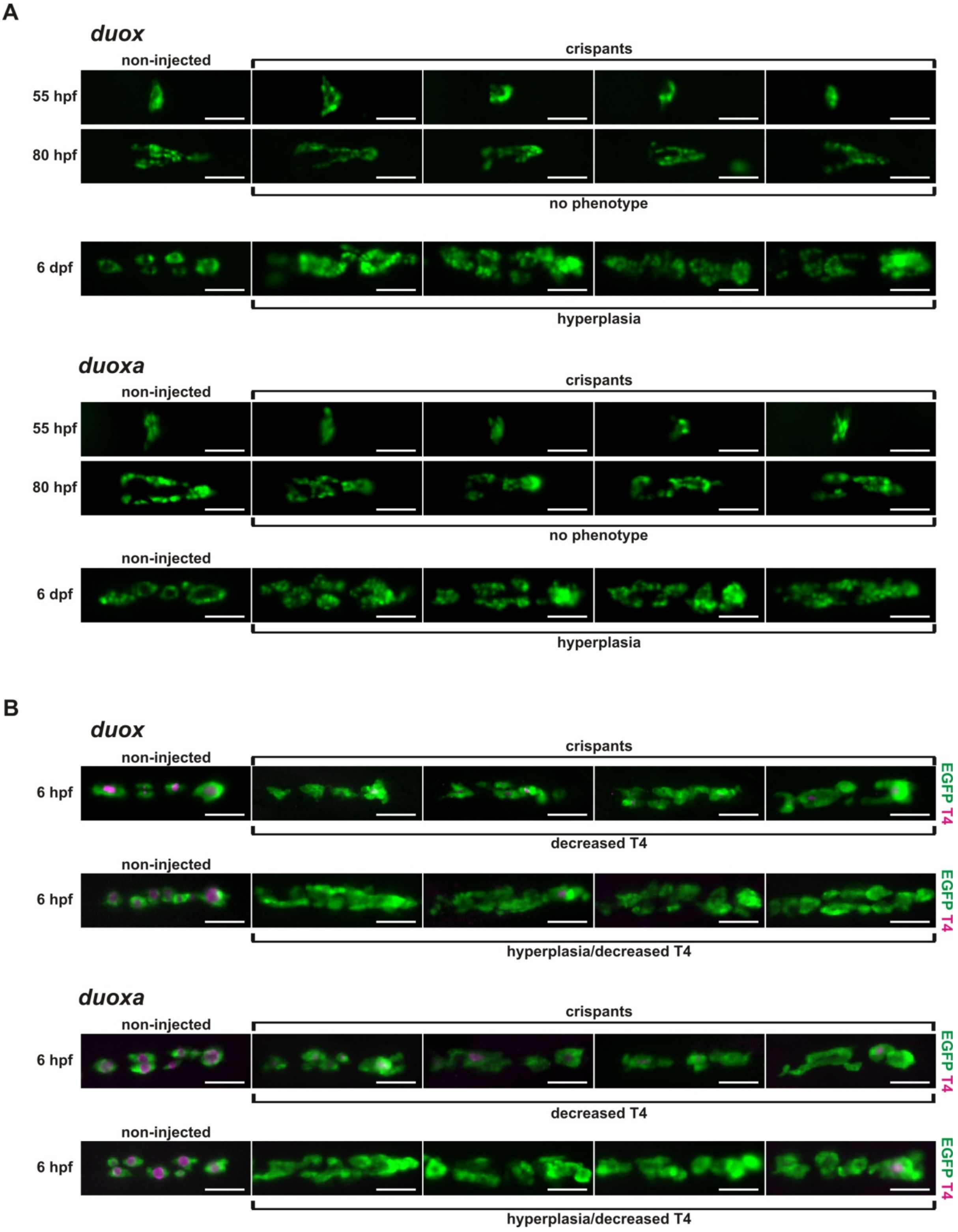

Supplementary Figure 4: Phenotypic spectrum of *duox* and *duoxa* crispants. (A) Epifluorescence live imaging of *Tg*(*tg:nlsEGFP*) zebrafish larvae injected with Cas9 protein and sgRNAs targeting *duox* or *duoxa*, respectively. Ventral views of the thyroid region (anterior to the right) are shown for non-injected control fish and four crispants. Images were aquired at 55 hpf, 80 hpf, and 6 dpf. No deviation from control thyroid morphology was

evident in *duox* and *duoxa* crispants at 55 and 80 hpf. By 6 dpf, hyperplastic thyroid enlargement (goiter) was detectable in many *duox* and *duoxa* crispants. (B) Whole-mount immunofluorescence staining of *Tg*(*tg:nlsEGFP*) zebrafish for EGFP and colloidal T4 at 6 dpf. Ventral views of the thyroid region (anterior to the right) are shown for non-injected control fish and different crispants displaying hypothyroidism (decreased T4 staining) that was often but not always associated with a hyperplastic thyroid enlargement. Scale bars: 50 μm.

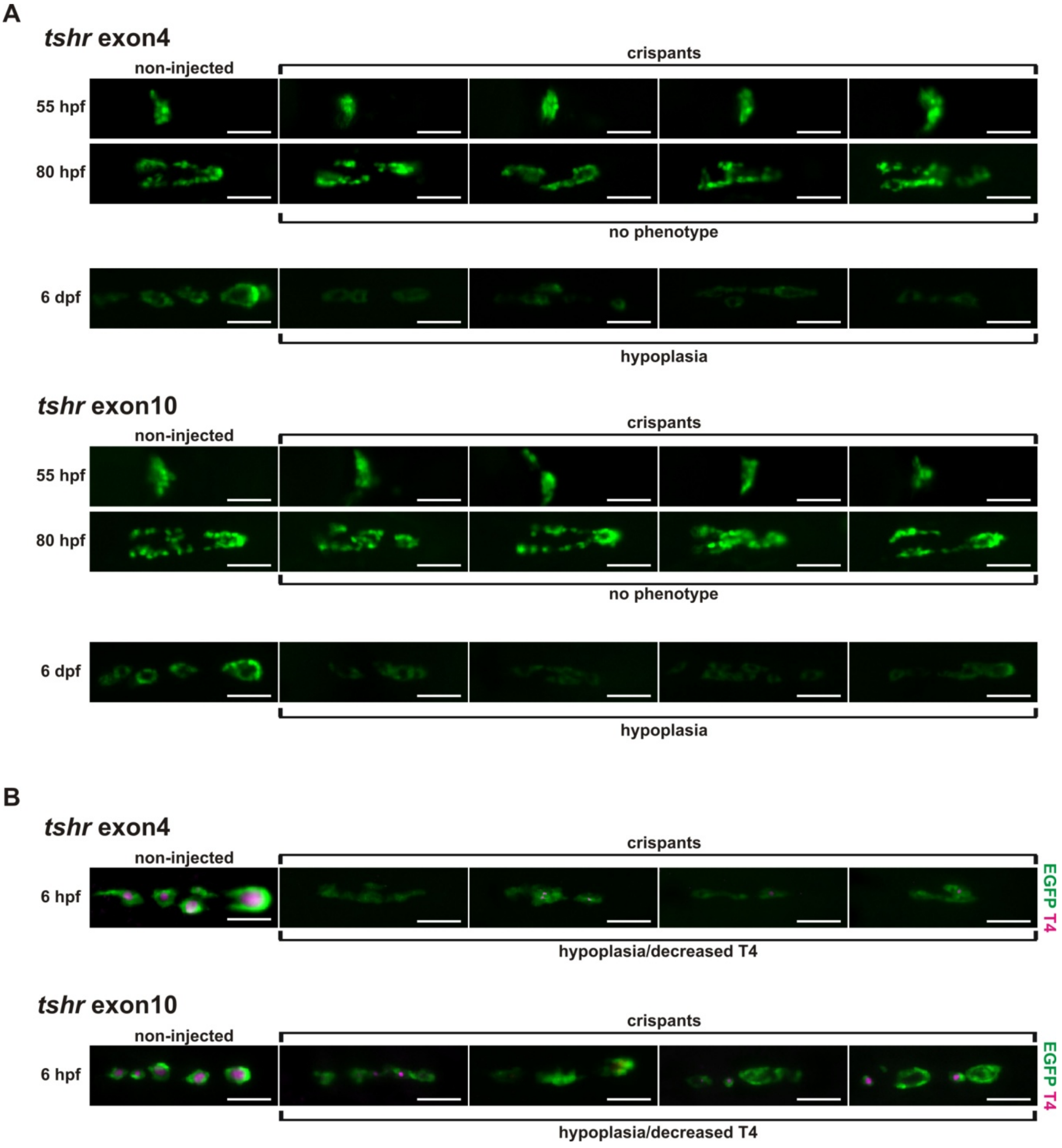

Supplementary Figure 5: Phenotypic spectrum of *tshr* crispants. (A) Epifluorescence live imaging of *Tg*(*tg:nlsEGFP*) zebrafish larvae injected with Cas9 protein and sgRNAs targeting exon 4 or exon 10 of *tshr*, respectively. Ventral views of the thyroid region (anterior to the right) are shown for non-injected control fish and four crispants. Images were aquired at 55 hpf, 80 hpf, and 6 dpf. No deviation from control thyroid morphology was evident in *tshr* crispants at 55 and 80 hpf. By 6 dpf, thyroids of *tshr* crispants had an atrophic/hypoplastic appearance and displayed a greatly reduced GFP reporter signal. (B) Whole-mount immunofluorescence staining of *Tg*(*tg:nlsEGFP*) zebrafish for EGFP and colloidal T4 at 6 dpf. Ventral views of the thyroid region (anterior to the right) are shown for non-injected

control fish and different *tshr* crispants displaying hypothyroidism (decreased T4 staining), variable thyroid hypoplasia and reduced GFP expression. Scale bars: 50 µm.

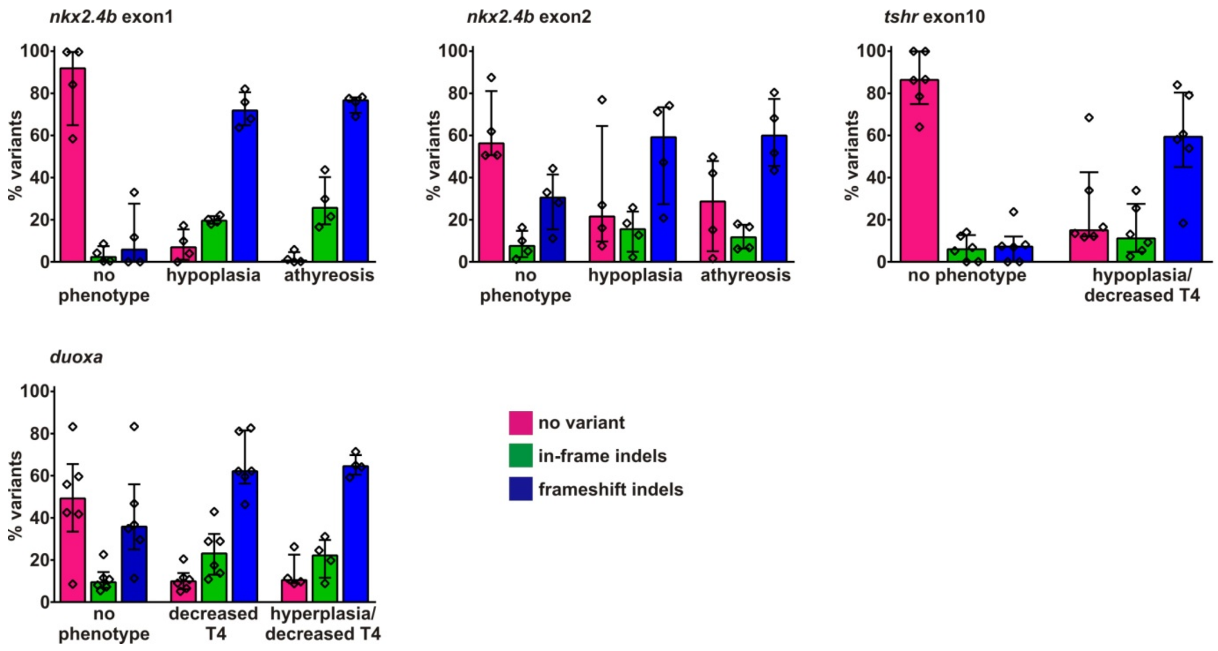

Supplementary Figure 6: Illumina HiSeq analyses of *nkx2.4b*, *duoxa* and *tshr* crispants. Distribution of allelic variants as determined by Illumina HiSeq analysis of individual crispants revealed high mutagenesis efficiency in larvae presenting thyroid dysgenesis (target: exon 1 of *nkx2.4b*, exon 2 of *nkx2.4b*), thyroid hypoplasia and decreased T4 content (target: exon 10 of *tshr*) and hypothyroidism/hyperplastic thyroid enlargement (target: *duoxa*). The percentage of WT alleles (no variant call), in-frame indels, or frameshift indels is shown for *N* = 4-6 larvae per phenotypic category (median values with interquartile range).

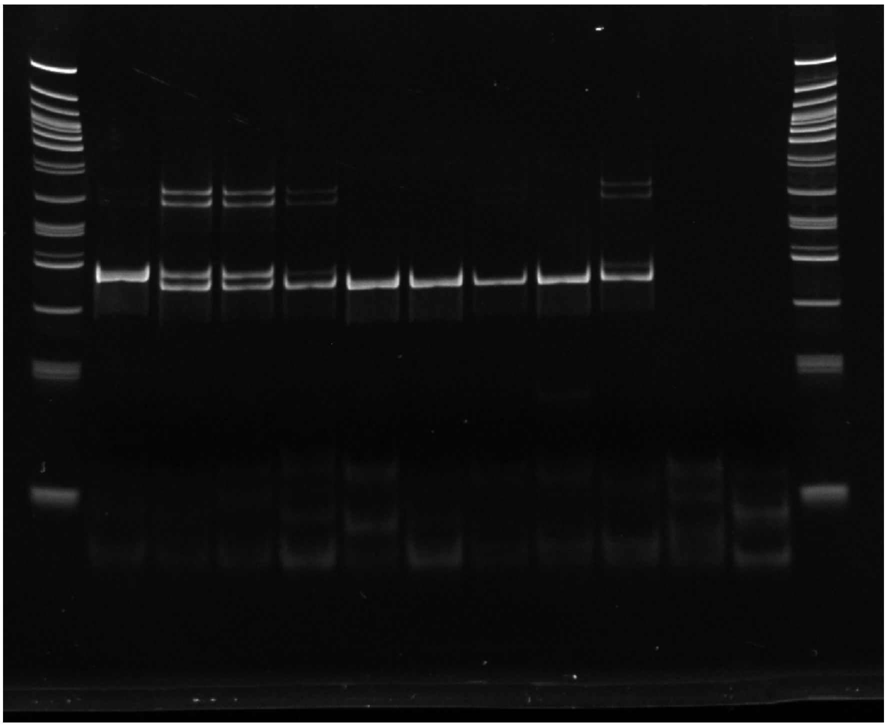

Supplementary Figure 7: Full length gel for the cropped gel image shown in Figure 7B. Polyacrylamide gel for PCR amplicons of wild-type fish (single PCR product of 178 bp), heterozygous carriers of the *duoxa* Δ11 allele (main PCR products of 178 and 167 bp) and homozygous carriers of the *duoxa* Δ11 allele (PCR product of 167 bp). DNA molecular size markers are shown in lane 1 and 13, wild-type sample in lane 2, heterozygous *duoxa* mutants in lane 3, 4, 5 and 10, homozygous *duoxa* mutants in lanes 6-9 and negative controls (H_2O) in lane 11 and 12. Additional slow-migrating heteroduplex bands can be seen in PCR samples of heterozygous *duoxa* mutant fish (lanes 3,4,5,10).

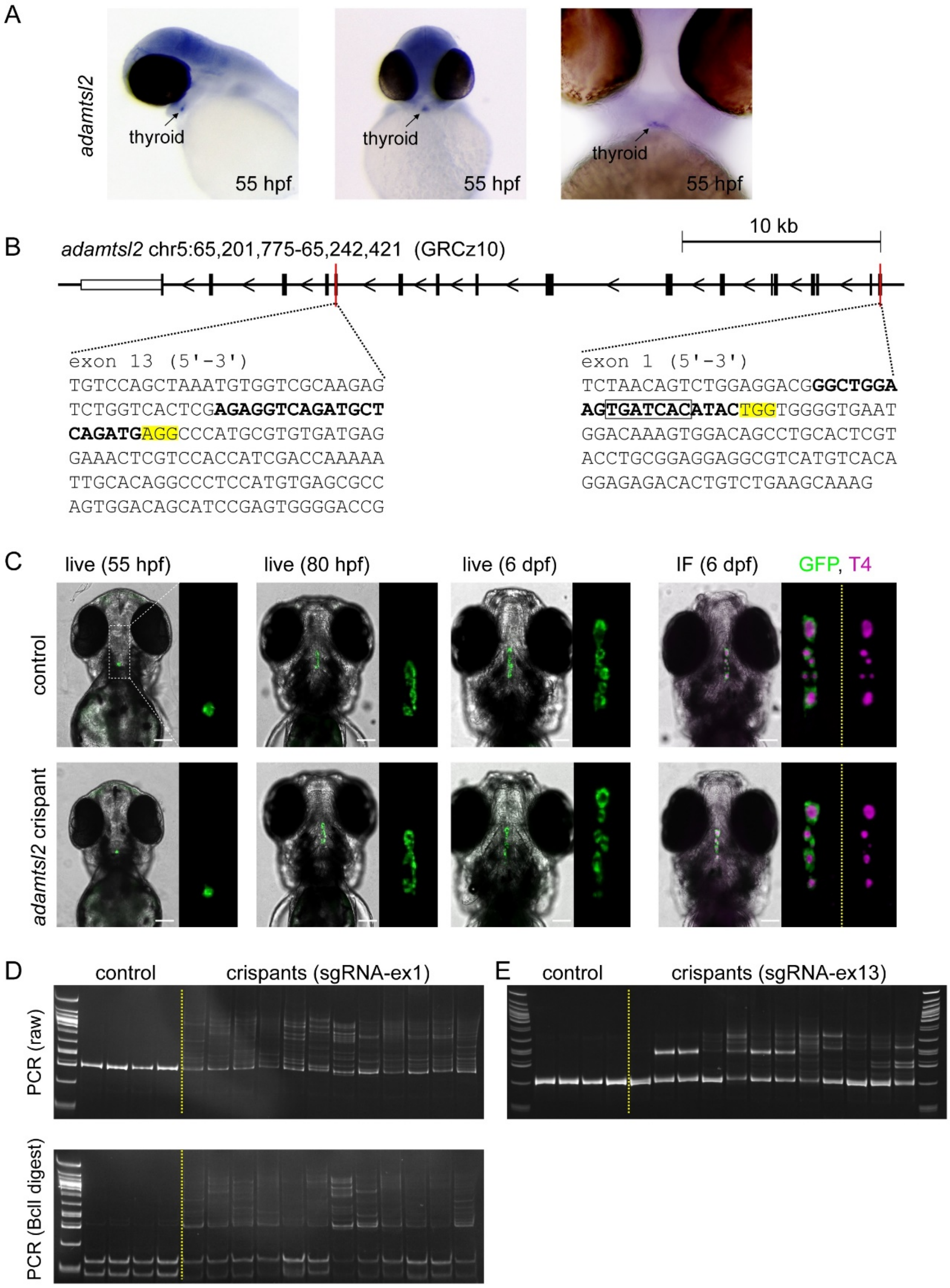

Supplementary Figure 8: Characterization of *adamtsl2* crispants. (A) *adamtsl2* mRNA is expressed at high levels in the zebrafish thyroid primordium (arrow) as demonstrated by whole-mount *in situ* hybridization. Lateral and frontal views of 55 hpf embryos stained with an *adamtsl2*-specific riboprobe are shown. (B) Zebrafish *adamtsl2* genomic locus on chromosome 5 with sequences for exon 1 and exon 13. Underlined sequences are target sites

for sgRNA-ex1 and sgRNA-ex13, respectively. PAM sequences are highlighted in yellow and the boxed sequence in exon 1 is the BclI restriction enzyme recognition site. (C) Thyroid phenotyping of *adamtsl2* crispants did not reveal alteration in thyroid morphogenesis or thyroid function. Results shown are from an injection experiment with sgRNA-ex13 and similar results were obtained with sgRNA-ex1. Epifluorescence live imaging of *Tg*(*tg:nlsEGFP*) zebrafish at 55 hpf, 80 hpf, and 6 dpf. Ventral views of the head region and 3-fold magnified views of the thyroid region (GFP channel only) are shown for non-injected controls and *adamtsl2* crispants. Whole-mount immunofluorescence (IF) staining of 6 dpf *Tg*(*tg:nlsEGFP*) zebrafish for EGFP (thyroid cells) and thyroxine (colloidal T4). Ventral views of the head region (anterior is to the top) and 3-fold magnified views of the thyroid region (GFP, T4) are shown. Scale bars: 100 µm. (D) Polyacrylamide gel electrophoresis (PAGE) of PCR amplicons generated with primers spanning the target site of sgRNA-ex1. Upper panel shows a gel for PCR amplicons of *WT* larvae (single PCR product of 206 bp) and *adamtsl2* crispants (multiple slow-migrating heteroduplex bands). Lower panel shows a gel for PCR amplicons digested with BclI. Note complete digestion of PCR amplicons from *WT* larvae resulting in two restriction fragments of 111bp and 95 bp, respectively. Images of full-length gels are shown in Supplementary Figure 9A,B. (E) PAGE analysis of PCR amplicons generated with primers spanning the target site of sgRNA-ex13. Gel showing PCR amplicons of non-injected *WT* larvae (single PCR product of 151 bp) and *adamtsl2* crispants (multiple slow-migrating heteroduplex bands in most samples). Full-length gel is shown in Supplementary Figure 9C.

A

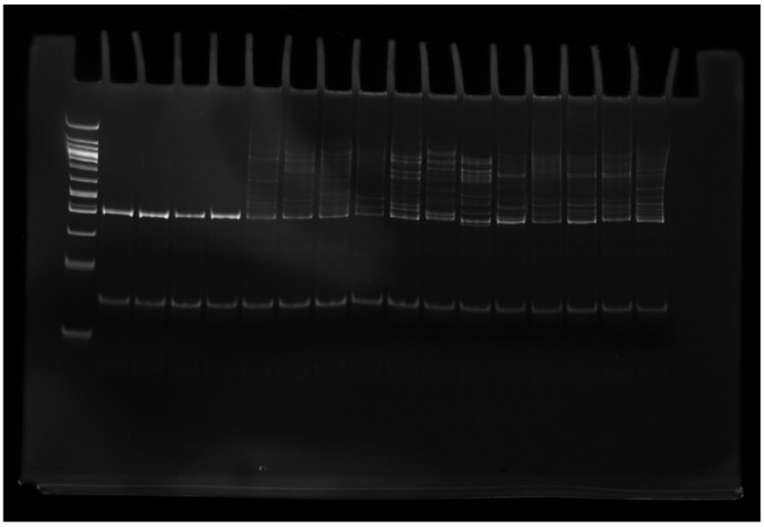

B

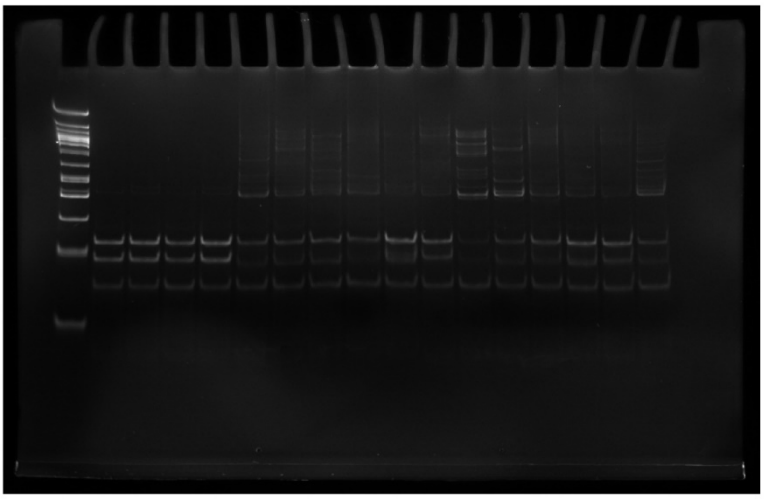

C

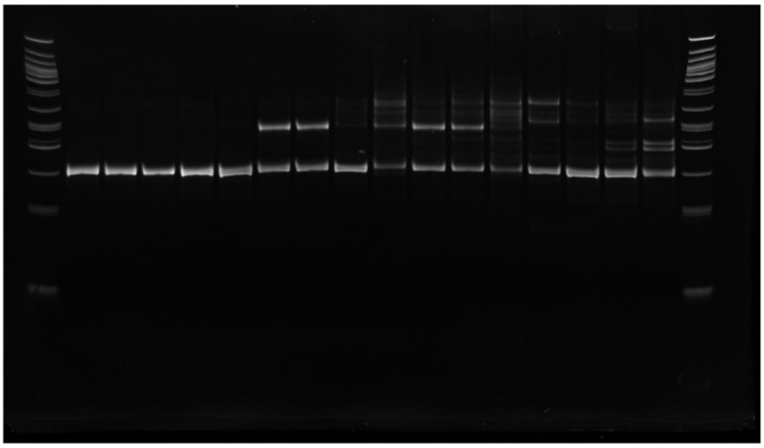

Supplementary Figure 9: Full length gels for the cropped gels shown in Supplementary Figure 8. (A) Full length gel for the cropped gel shown in the upper panel of Supplementary Figure 8D. (B) Full length gel for the cropped gel shown in the lower panel of Supplementary Figure 8D. (C) Full length gel for the cropped gel shown in Supplementary Figure 8E.

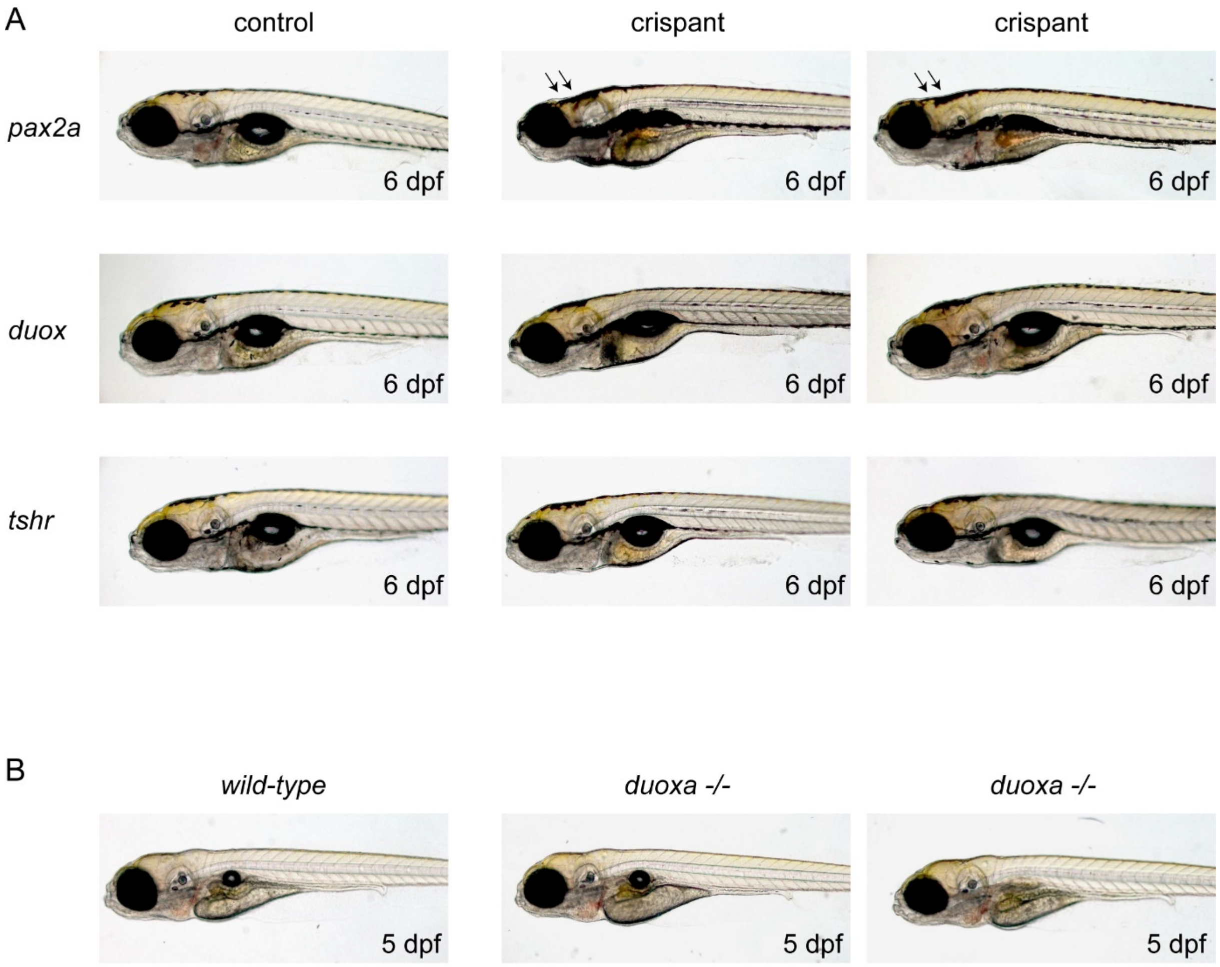

Supplementary Figure 10: Brightfield micrographs of crispants and germline mutant fish (A) Brightfield microscopy of control larvae and crispants displaying a thyroid phenotype after injection of gRNAs targetting *pax2a*, *duox* and *tshr*. Lateral views (anterior to the left) of 6 dpf old fish are shown. Arrows highlight defective brain development in *pax2a* crispants. (B) Brightfield microscopy of *WT* larvae and homozygous carriers of the *duoxa* Δ11 allele. Lateral views (anterior to the left) of 5 dpf old larvae (pigment-less *casper* fish) are shown.

Supplementary Information 2: NGS data

A Rapid CRISPR/Cas-based Mutagenesis Assay in Zebrafish for Identification of Genes Involved in Thyroid Morphogenesis and Function

Trubiroha, A.[1,a], Gillotay, P.[1], Giusti, N.[1], Gacquer, D.[1], Libert, F.[1], Lefort, A.[1], Haerlingen, ,B.[1], De Deken, X.[1], Opitz, R.[1,2] and Costagliola, S.[1]

[1] Institute of Interdisciplinary Research in Molecular Human Biology (IRIBHM), Université Libre de Bruxelles, Route de Lennik 808, 1070 Brussels, Belgium

[2] Institute of Experimental Pediatric Endocrinology, Charité Universitätsmedizin Berlin, Augustenburger Platz 1, 13353 Berlin, Germany

A. Trubiroha, P. Gillotay and N. Giusti contributed equally to this work

[a] present address: German Federal Institute for Risk Assessment (BfR), Department Chemicals and Product Safety, Max-Dohrn-Strasse 8-10, 10589 Berlin, Germany

Corresponding authors:

Sabine Costagliola (scostag@ulb.ac.be)

Robert Opitz (robert.opitz@charite.de)

Allele frequencies of individual F0 crispants as analysed by CrispRVariants.

Data of HiSeq sequencing of zebrafish mutagenesis experiments were analysed by CrispRVariants[26,45]. For each target locus, variant allele plots and barplots are presented for individual zebrafish categorized by phenotype. Only variants with a frequency of ≥ 5% in at least one zebrafish were considered.

Variant allele plots show on the left the alignment of each variant to the reference genome (top line) with the sgRNA sequence and the 3' PAM indicated by boxes. The cut site is indicated by a vertical line. The gene scheme on the top shows the binding site of the sgRNA (in red) in relation to known Ensemble transcripts. Within the alignment, deletions and insertions are indicated by ´-` and by symbols, respectively, with the corresponding insert sequence explained below the plot. Deletions, insertions, single nucleotide variants and chimeric reads are indicated by D, I, SNV, or 'other' respectively. Each variant is further characterized by its size and by the position of the most upstream base in relation to the cut site. For example, a 4 bp deletion starting at -4 bases upstream of the cut site would be -4:4D. The right panel of the variant allele plots shows the frequency (in read counts) of each variant for individual zebrafish with the top line indicating the total number of derived sequences. Color codes on the right represent the relative frequencies in percentages of each variant.

Barplots show the relative frequencies (x-axis) of no variant alleles, frameshift indels, inframe indels, single nucleotide variants, and chimeric reads for individual zebrafish (Y-axis) as indicated by color codes. The number of different alleles (variants) and the total number of sequences for each individual zebrafish are shown to the right of the barplots.

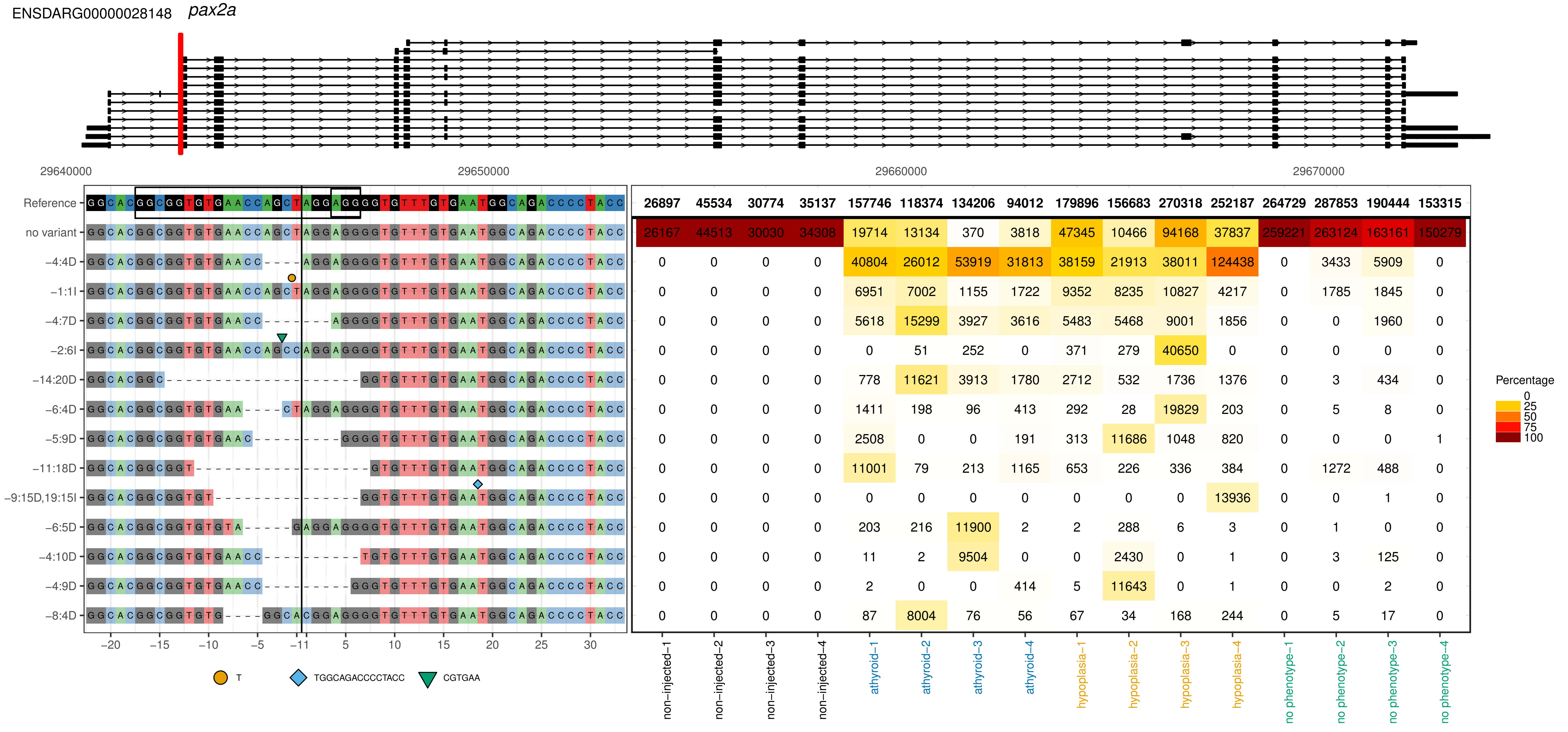
ENSDARG00000028148 pax2a
29640000
29650000
29660000
29670000
Reference
no variant
−4:4D
−1:1I
−4:7D
−2:6I
−14:20D
−6:4D
−5:9D
−11:18D
−9:15D,19:15I
−6:5D
−4:10D
−4:9D
−8:4D
−20 −15 −10 −5 −1 1 5 10 15 20 25 30
T
TGGCAGACCCCTACC
CGTGAA
26897 45534 30774 35137 157746 118374 134206 94012 179896 156683 270318 252187 264729 287853 190444 153315
26167 44513 30030 34308 19714 13134 370 3818 47345 10466 94168 37837 259221 263124 163161 150279
0 0 0 0 40804 26012 53919 31813 38159 21913 38011 124438 0 3433 5909 0
0 0 0 0 6951 7002 1155 1722 9352 8235 10827 4217 0 1785 1845 0
0 0 0 0 5618 15299 3927 3616 5483 5468 9001 1856 0 0 1960 0
0 0 0 0 0 51 252 0 371 279 40650 0 0 0 0 0
0 0 0 0 778 11621 3913 1780 2712 532 1736 1376 0 3 434 0
0 0 0 0 1411 198 96 413 292 28 19829 203 0 5 8 0
0 0 0 0 2508 0 0 191 313 11686 1048 820 0 0 0 1
0 0 0 0 11001 79 213 1165 653 226 336 384 0 1272 488 0
0 0 0 0 0 0 0 0 0 0 0 13936 0 0 1 0
0 0 0 0 203 216 11900 2 2 288 6 3 0 1 0 0
0 0 0 0 11 2 9504 0 0 2430 0 1 0 3 125 0
0 0 0 0 2 0 0 414 5 11643 0 1 0 0 2 0
0 0 0 0 87 8004 76 56 67 34 168 244 0 5 17 0
non−injected−1
non−injected−2
non−injected−3
non−injected−4
athyroid−1
athyroid−2
athyroid−3
athyroid−4
hypoplasia−1
hypoplasia−2
hypoplasia−3
hypoplasia−4
no phenotype−1
no phenotype−2
no phenotype−3
no phenotype−4
Percentage
0
25
50
75
100

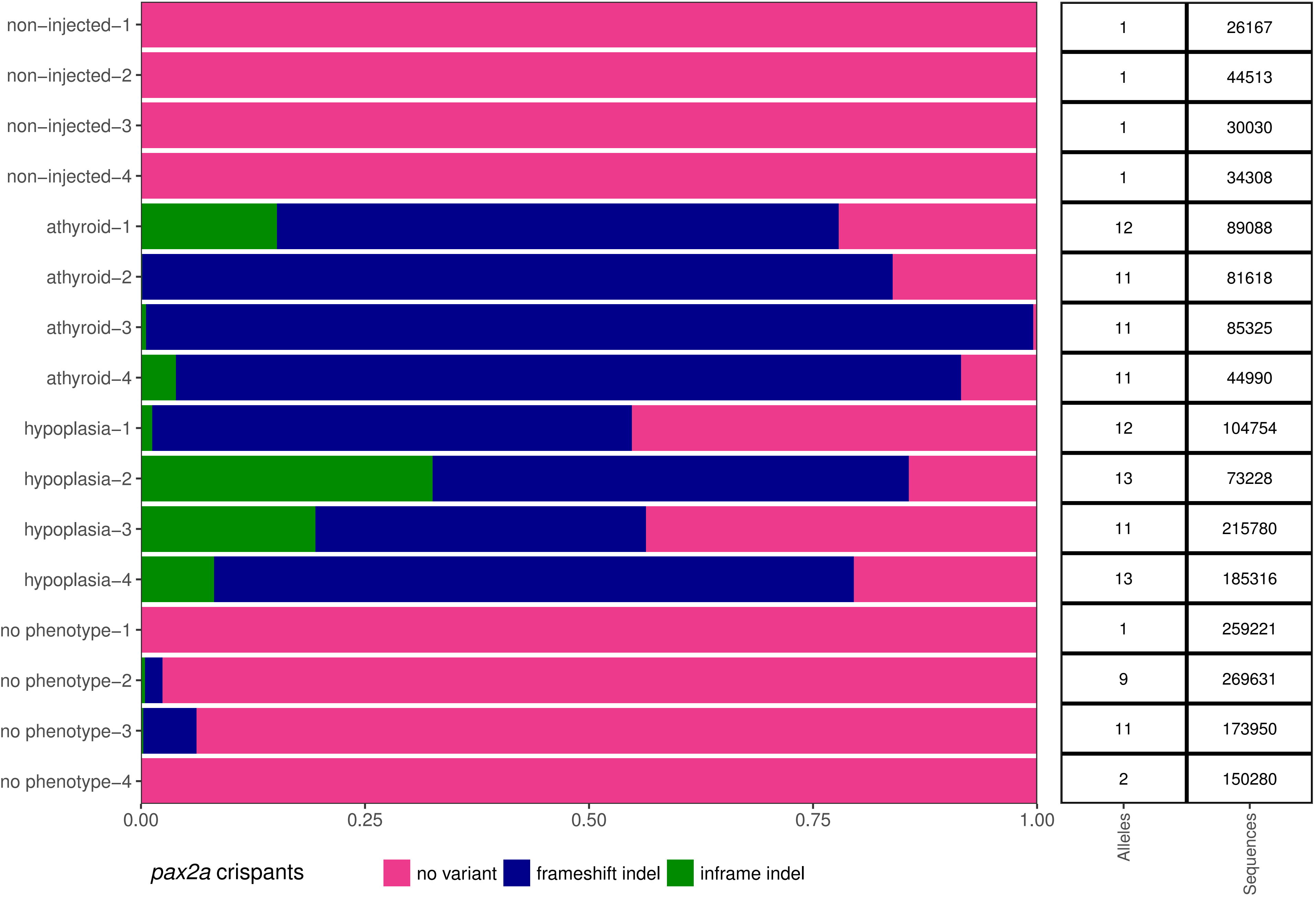

non−injected−1
non−injected−2
non−injected−3
non−injected−4
athyroid−1
athyroid−2
athyroid−3
athyroid−4
hypoplasia−1
hypoplasia−2
hypoplasia−3
hypoplasia−4
no phenotype−1
no phenotype−2
no phenotype−3
no phenotype−4
0.00
0.25
0.50
0.75
1.00
Alleles
Sequences
1 26167
1 44513
1 30030
1 34308
12 89088
11 81618
11 85325
11 44990
12 104754
13 73228
11 215780
13 185316
1 259221
9 269631
11 173950
2 150280
pax2a crispants
no variant
frameshift indel
inframe indel

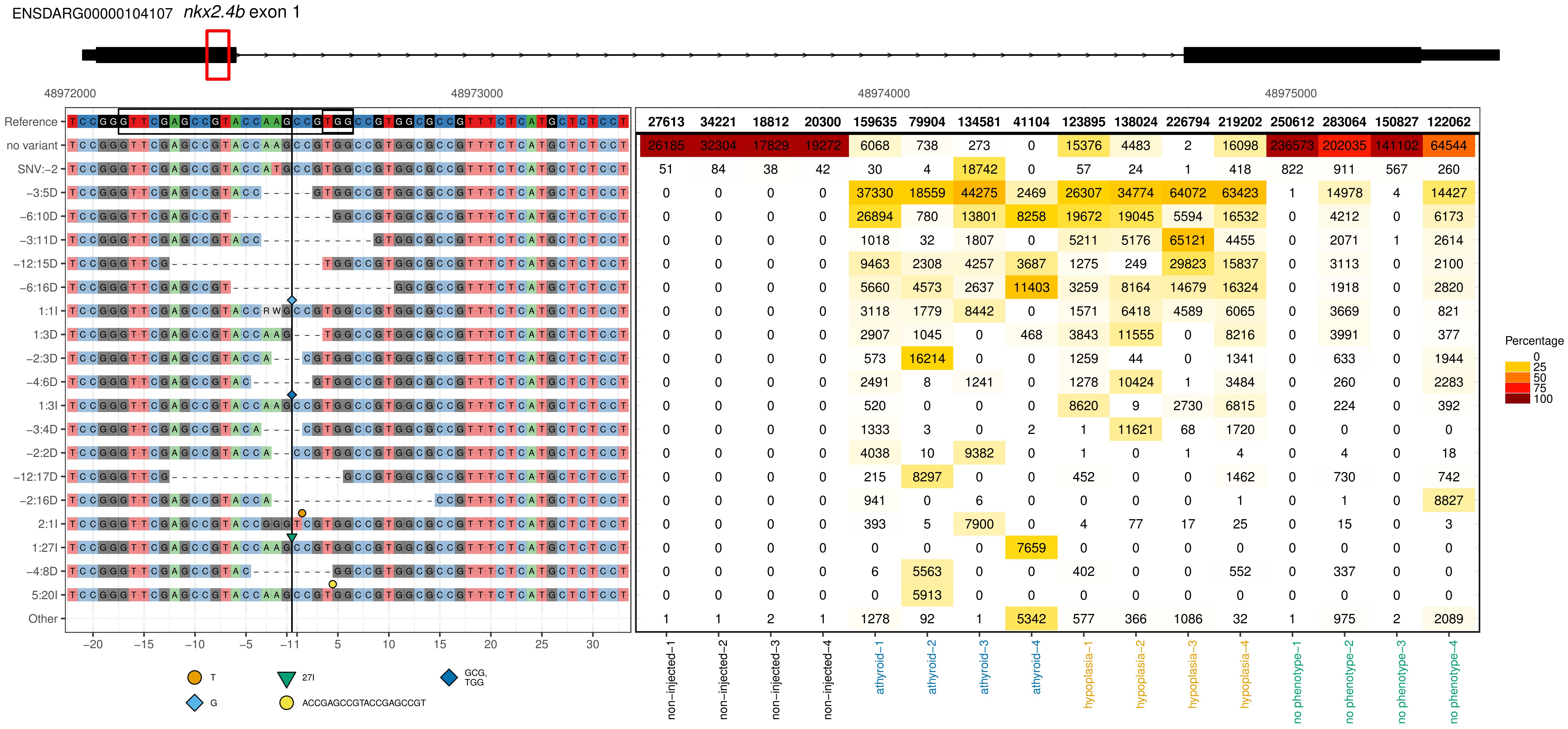

	non-injected-1	non-injected-2	non-injected-3	non-injected-4	athyroid-1	athyroid-2	athyroid-3	athyroid-4	hypoplasia-1	hypoplasia-2	hypoplasia-3	hypoplasia-4	no phenotype-1	no phenotype-2	no phenotype-3	no phenotype-4
Reference	27613	34221	18812	20300	159635	79904	134581	41104	123895	138024	226794	219202	250612	283064	150827	122062
no variant	26185	32304	17829	19272	6068	738	273	0	15376	4483	2	16098	236573	202035	141102	64544
SNV:−2	51	84	38	42	30	4	18742	0	57	24	1	418	822	911	567	260
−3:5D	0	0	0	0	37330	18559	44275	2469	26307	34774	64072	63423	1	14978	4	14427
−6:10D	0	0	0	0	26894	780	13801	8258	19672	19045	5594	16532	0	4212	0	6173
−3:11D	0	0	0	0	1018	32	1807	0	5211	5176	65121	4455	0	2071	1	2614
−12:15D	0	0	0	0	9463	2308	4257	3687	1275	249	29823	15837	0	3113	0	2100
−6:16D	0	0	0	0	5660	4573	2637	11403	3259	8164	14679	16324	0	1918	0	2820
1:1I	0	0	0	0	3118	1779	8442	0	1571	6418	4589	6065	0	3669	0	821
1:3D	0	0	0	0	2907	1045	0	468	3843	11555	0	8216	0	3991	0	377
−2:3D	0	0	0	0	573	16214	0	0	1259	44	0	1341	0	633	0	1944
−4:6D	0	0	0	0	2491	8	1241	0	1278	10424	1	3484	0	260	0	2283
1:3I	0	0	0	0	520	0	0	0	8620	9	2730	6815	0	224	0	392
−3:4D	0	0	0	0	1333	3	0	2	1	11621	68	1720	0	0	0	0
−2:2D	0	0	0	0	4038	10	9382	0	1	0	1	4	0	4	0	18
−12:17D	0	0	0	0	215	8297	0	0	452	0	0	1462	0	730	0	742
−2:16D	0	0	0	0	941	0	6	0	0	0	0	1	0	1	0	8827
2:1I	0	0	0	0	393	5	7900	0	4	77	17	25	0	15	0	3
1:27I	0	0	0	0	0	0	0	7659	0	0	0	0	0	0	0	0
−4:8D	0	0	0	0	6	5563	0	0	402	0	0	552	0	337	0	0
5:20I	0	0	0	0	0	5913	0	0	0	0	0	0	0	0	0	0
Other	1	1	2	1	1278	92	1	5342	577	366	1086	32	1	975	2	2089

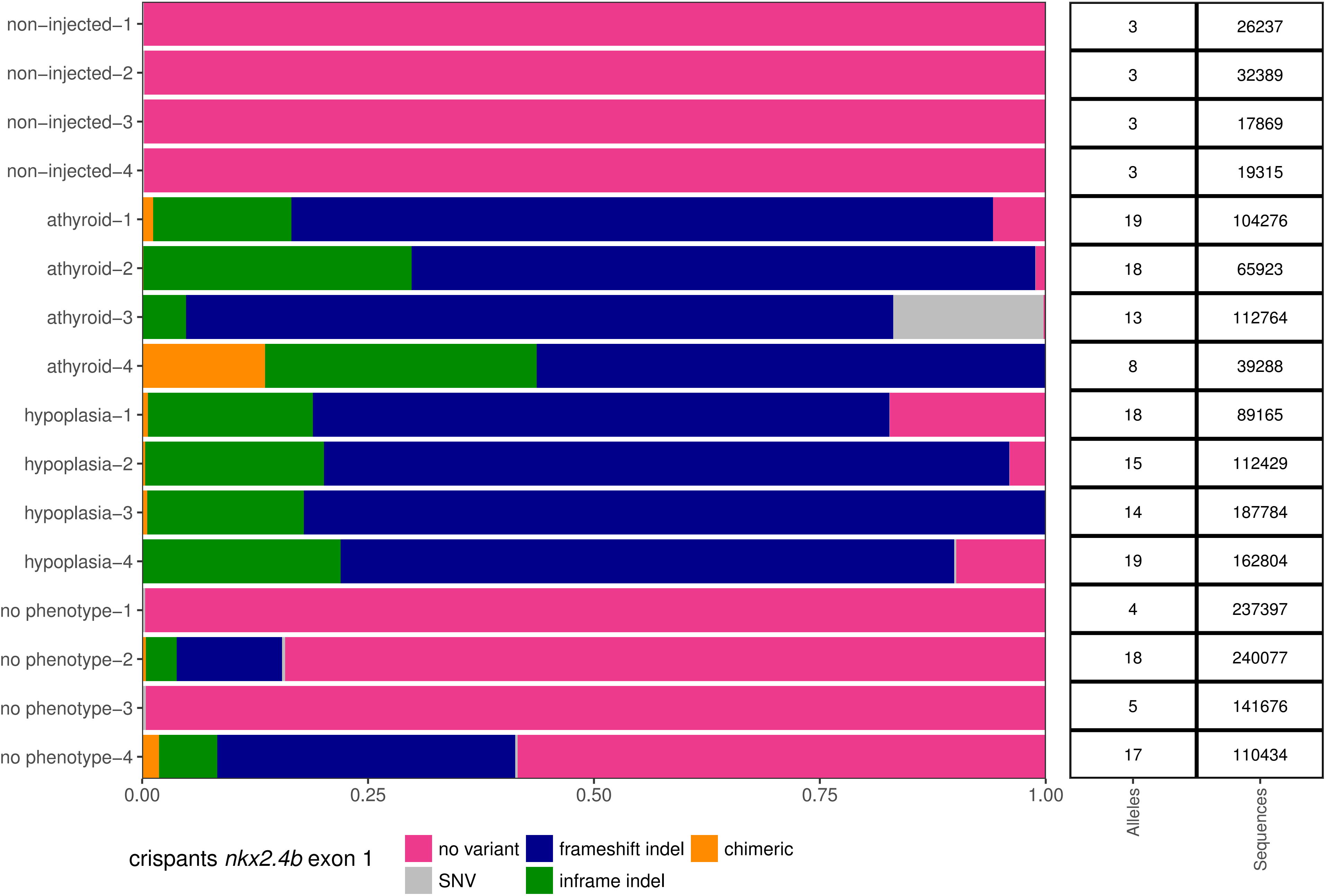
non−injected−1
non−injected−2
non−injected−3
non−injected−4
athyroid−1
athyroid−2
athyroid−3
athyroid−4
hypoplasia−1
hypoplasia−2
hypoplasia−3
hypoplasia−4
no phenotype−1
no phenotype−2
no phenotype−3
no phenotype−4
0.00
0.25
0.50
0.75
1.00
Alleles
Sequences
3 26237
3 32389
3 17869
3 19315
19 104276
18 65923
13 112764
8 39288
18 89165
15 112429
14 187784
19 162804
4 237397
18 240077
5 141676
17 110434
crispants *nkx2.4b* exon 1
no variant
frameshift indel
chimeric
SNV
inframe indel

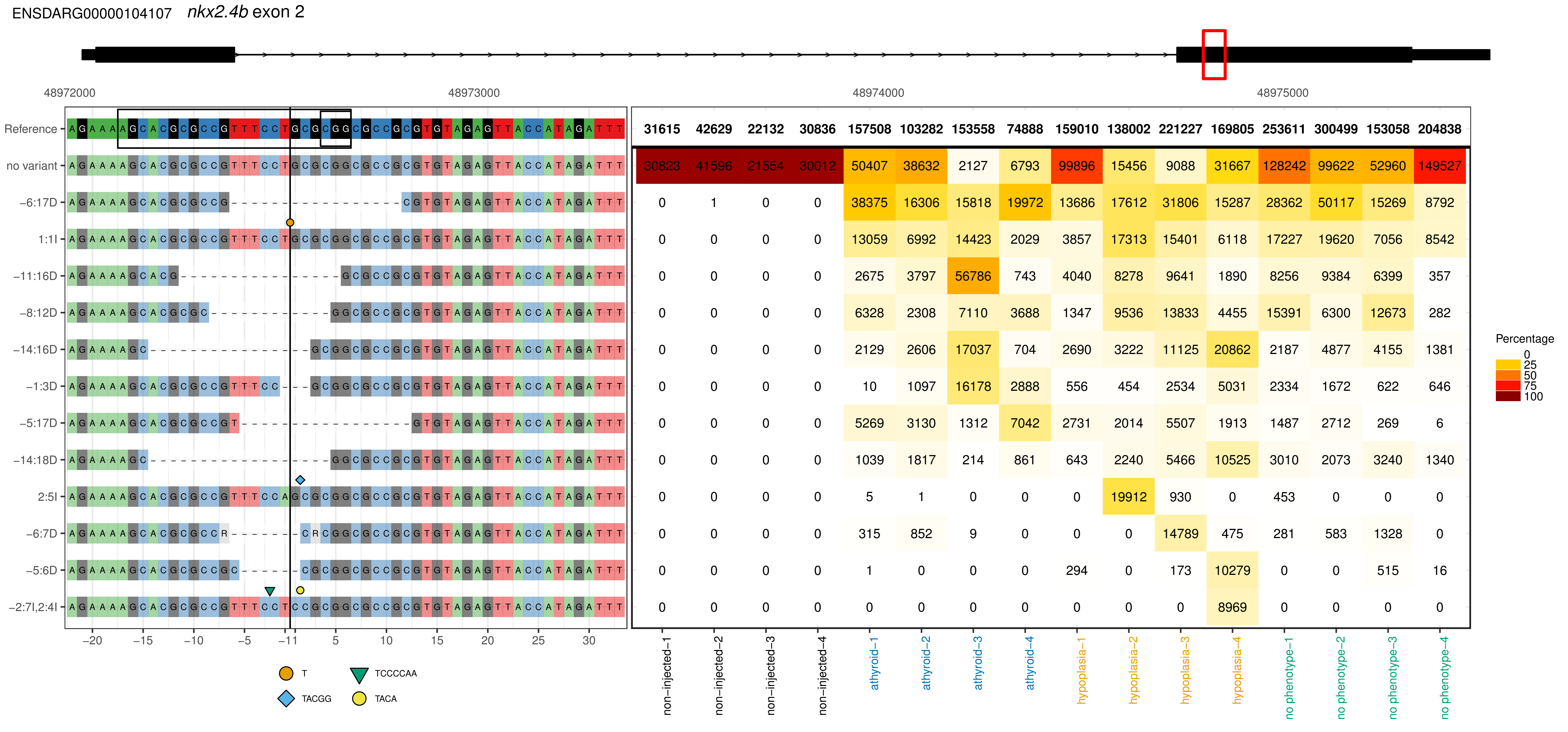

ENSDARG00000104107 nkx2.4b exon 2
48972000
48973000
48974000
48975000
Reference
no variant
−6:17D
1:1I
−11:16D
−8:12D
−14:16D
−1:3D
−5:17D
−14:18D
2:5I
−6:7D
−5:6D
−2:7I,2:4I
−20 −15 −10 −5 −1 1 5 10 15 20 25 30
T
TCCCCAA
TACGG
TACA
31615 42629 22132 30836 157508 103282 153558 74888 159010 138002 221227 169805 253611 300499 153058 204838
30823 41596 21554 30012 50407 38632 2127 6793 99896 15456 9088 31667 128242 99622 52960 149527
0 1 0 0 38375 16306 15818 19972 13686 17612 31806 15287 28362 50117 15269 8792
0 0 0 0 13059 6992 14423 2029 3857 17313 15401 6118 17227 19620 7056 8542
0 0 0 0 2675 3797 56786 743 4040 8278 9641 1890 8256 9384 6399 357
0 0 0 0 6328 2308 7110 3688 1347 9536 13833 4455 15391 6300 12673 282
0 0 0 0 2129 2606 17037 704 2690 3222 11125 20862 2187 4877 4155 1381
0 0 0 0 10 1097 16178 2888 556 454 2534 5031 2334 1672 622 646
0 0 0 0 5269 3130 1312 7042 2731 2014 5507 1913 1487 2712 269 6
0 0 0 0 1039 1817 214 861 643 2240 5466 10525 3010 2073 3240 1340
0 0 0 0 5 1 0 0 0 19912 930 0 453 0 0 0
0 0 0 0 315 852 9 0 0 0 14789 475 281 583 1328 0
0 0 0 0 1 0 0 0 294 0 173 10279 0 0 515 16
0 0 0 0 0 0 0 0 0 0 0 8969 0 0 0 0
Percentage
0
25
50
75
100
non−injected−1
non−injected−2
non−injected−3
non−injected−4
athyroid−1
athyroid−2
athyroid−3
athyroid−4
hypoplasia−1
hypoplasia−2
hypoplasia−3
hypoplasia−4
no phenotype−1
no phenotype−2
no phenotype−3
no phenotype−4

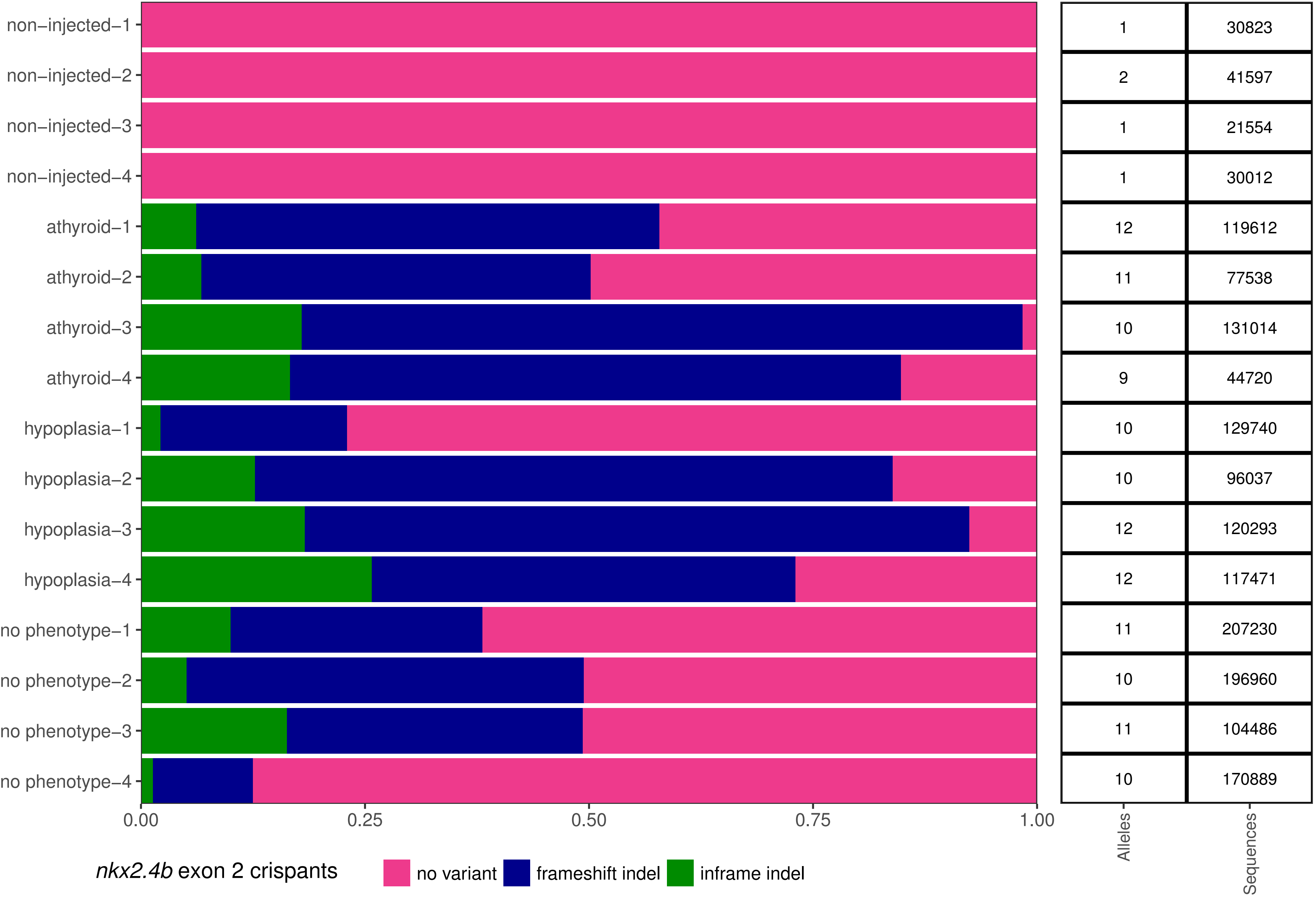

non–injected–1
non–injected–2
non–injected–3
non–injected–4
athyroid–1
athyroid–2
athyroid–3
athyroid–4
hypoplasia–1
hypoplasia–2
hypoplasia–3
hypoplasia–4
no phenotype–1
no phenotype–2
no phenotype–3
no phenotype–4
0.00
0.25
0.50
0.75
1.00
1 30823
2 41597
1 21554
1 30012
12 119612
11 77538
10 131014
9 44720
10 129740
10 96037
12 120293
12 117471
11 207230
10 196960
11 104486
10 170889
Alleles
Sequences
nkx2.4b exon 2 crispants
no variant
frameshift indel
inframe indel

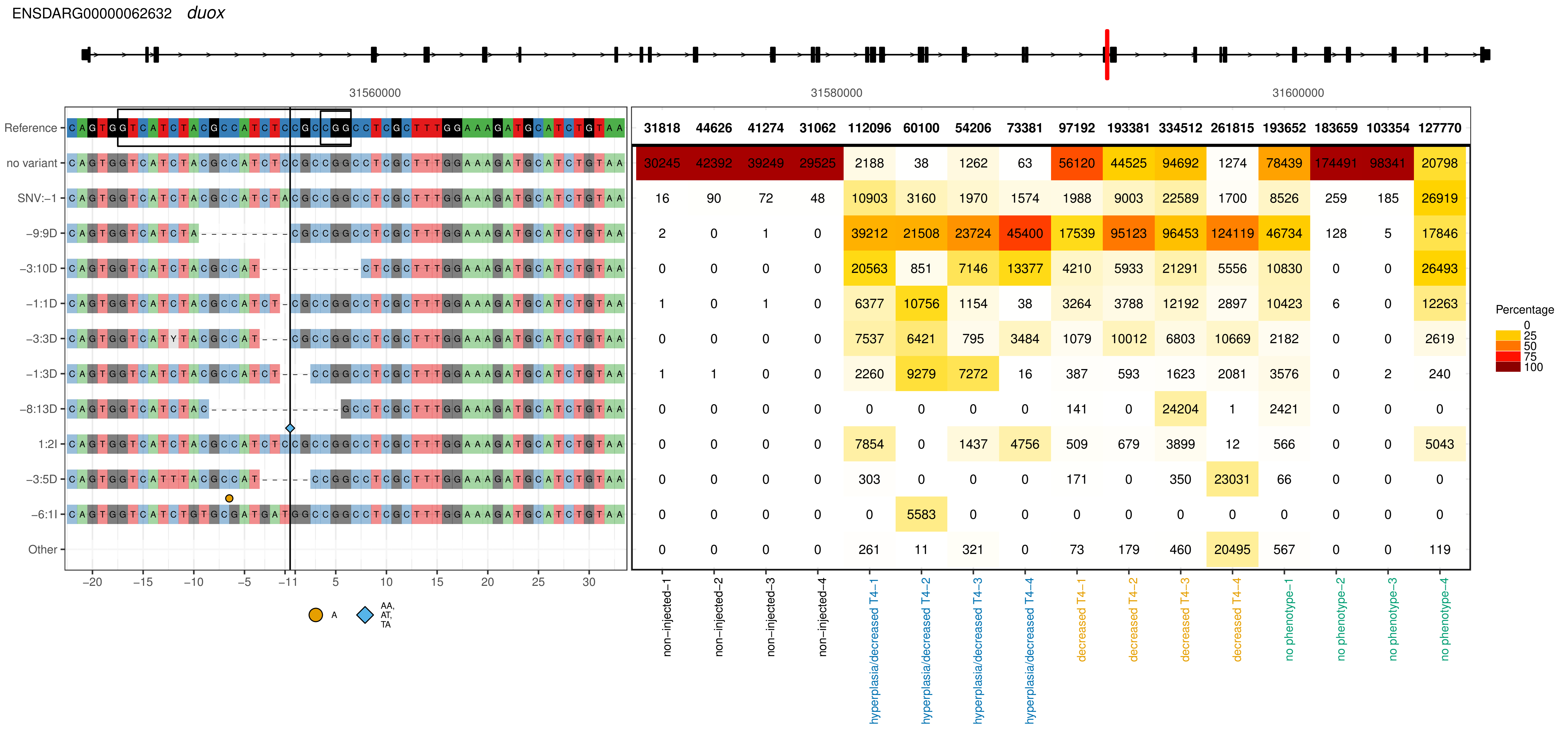

ENSDARG00000062632 duox
31560000
31580000
31600000
Reference
no variant
SNV:−1
−9:9D
−3:10D
−1:1D
−3:3D
−1:3D
−8:13D
1:2I
−3:5D
−6:1I
Other
−20 −15 −10 −5 −1 1 5 10 15 20 25 30
A
AA, AT, TA
31818 44626 41274 31062 112096 60100 54206 73381 97192 193381 334512 261815 193652 183659 103354 127770
30245 42392 39249 29525 2188 38 1262 63 56120 44525 94692 1274 78439 174491 98341 20798
16 90 72 48 10903 3160 1970 1574 1988 9003 22589 1700 8526 259 185 26919
2 0 1 0 39212 21508 23724 45400 17539 95123 96453 124119 46734 128 5 17846
0 0 0 0 20563 851 7146 13377 4210 5933 21291 5556 10830 0 0 26493
1 0 1 0 6377 10756 1154 38 3264 3788 12192 2897 10423 6 0 12263
0 0 0 0 7537 6421 795 3484 1079 10012 6803 10669 2182 0 0 2619
1 1 0 0 2260 9279 7272 16 387 593 1623 2081 3576 0 2 240
0 0 0 0 0 0 0 0 141 0 24204 1 2421 0 0 0
0 0 0 0 7854 0 1437 4756 509 679 3899 12 566 0 0 5043
0 0 0 0 303 0 0 0 171 0 350 23031 66 0 0 0
0 0 0 0 0 5583 0 0 0 0 0 0 0 0 0 0
0 0 0 0 261 11 321 0 73 179 460 20495 567 0 0 119
Percentage
0
25
50
75
100
non−injected−1
non−injected−2
non−injected−3
non−injected−4
hyperplasia/decreased T4−1
hyperplasia/decreased T4−2
hyperplasia/decreased T4−3
hyperplasia/decreased T4−4
decreased T4−1
decreased T4−2
decreased T4−3
decreased T4−4
no phenotype−1
no phenotype−2
no phenotype−3
no phenotype−4

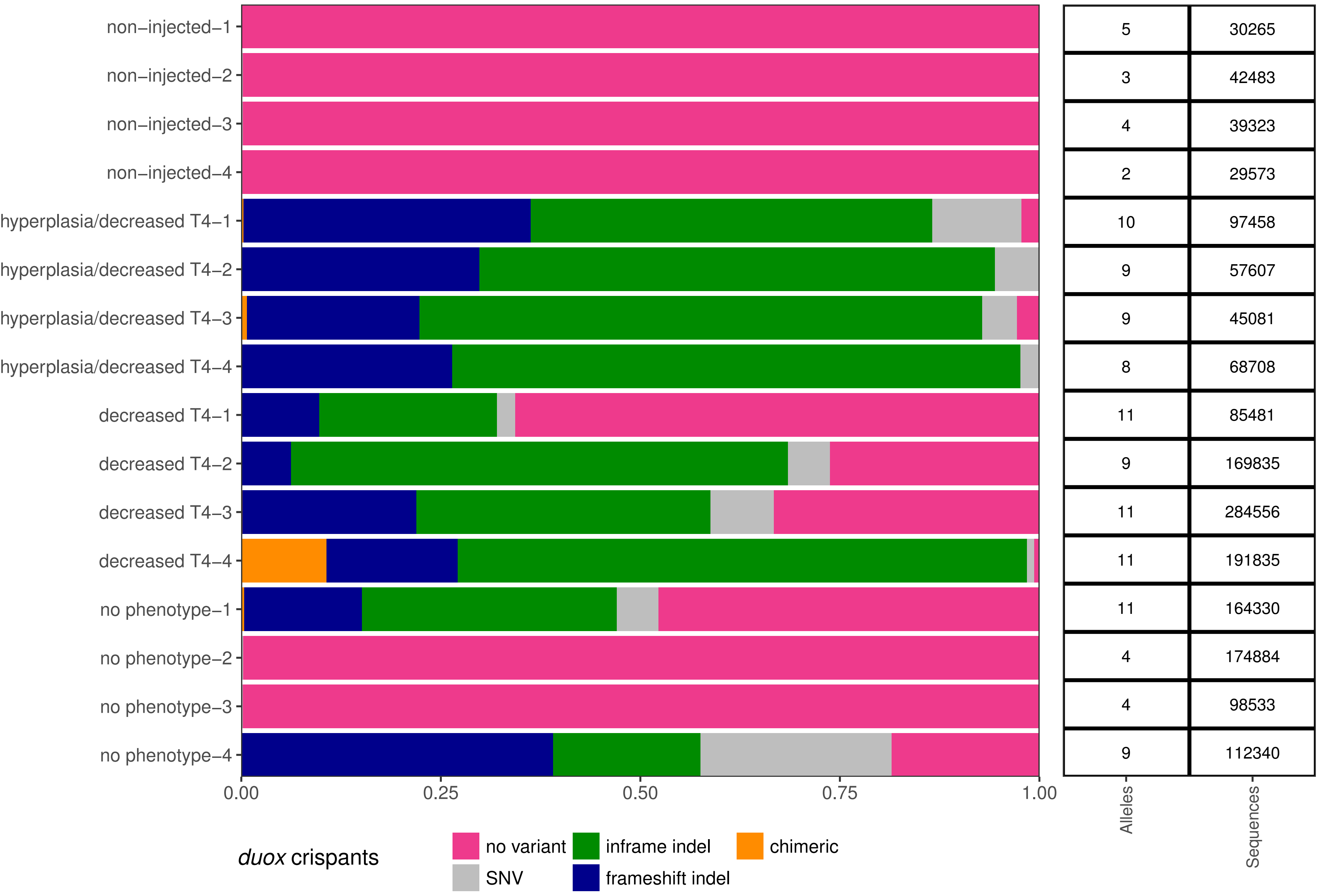

non−injected−1
non−injected−2
non−injected−3
non−injected−4
hyperplasia/decreased T4−1
hyperplasia/decreased T4−2
hyperplasia/decreased T4−3
hyperplasia/decreased T4−4
decreased T4−1
decreased T4−2
decreased T4−3
decreased T4−4
no phenotype−1
no phenotype−2
no phenotype−3
no phenotype−4
0.00
0.25
0.50
0.75
1.00
Alleles
Sequences
5 30265
3 42483
4 39323
2 29573
10 97458
9 57607
9 45081
8 68708
11 85481
9 169835
11 284556
11 191835
11 164330
4 174884
4 98533
9 112340
duox crispants
no variant
inframe indel
chimeric
SNV
frameshift indel

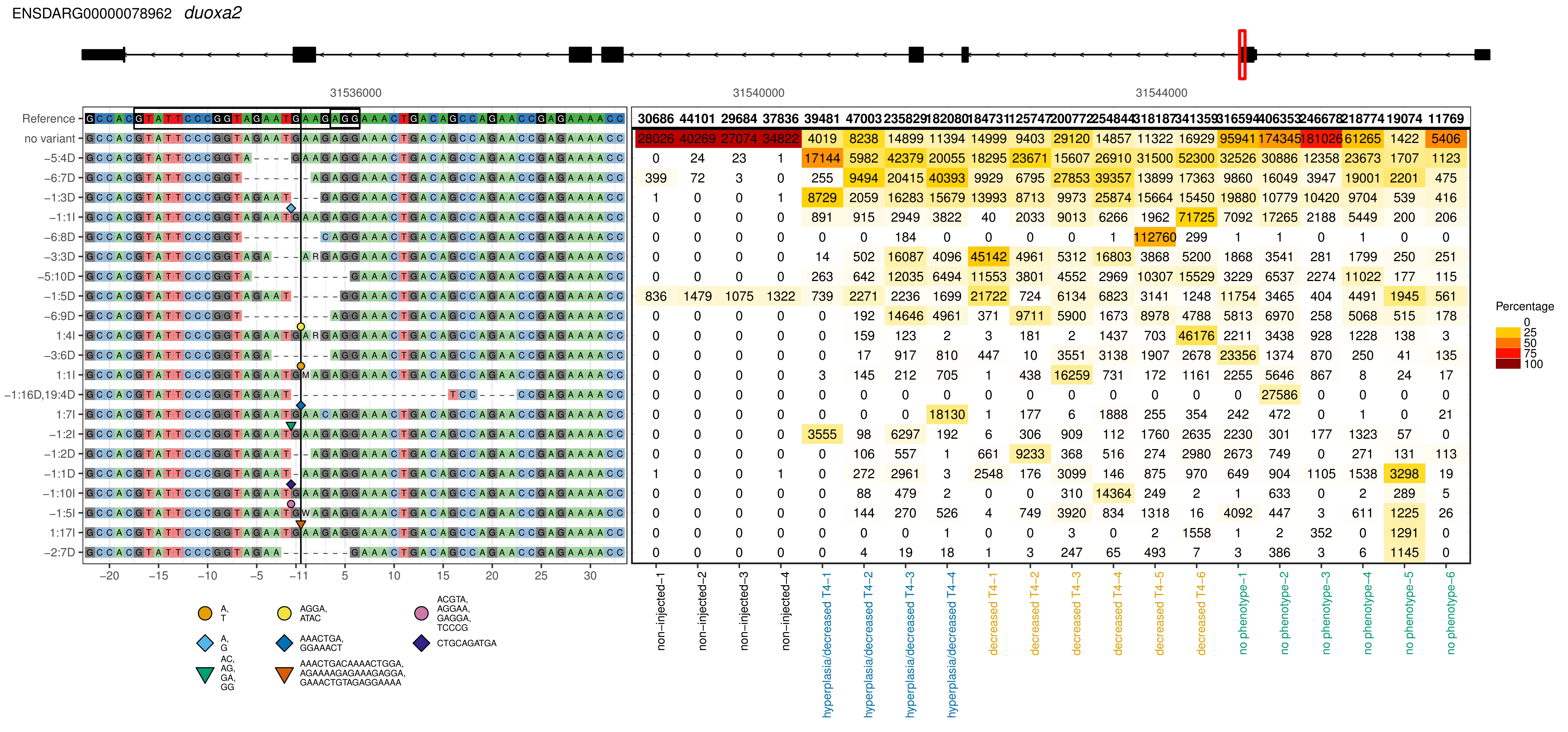

ENSDARG00000078962 duoxa2
31536000
31540000
31544000
Reference
no variant
−5:4D
−6:7D
−1:3D
−1:1I
−6:8D
−3:3D
−5:10D
−1:5D
−6:9D
1:4I
−3:6D
1:1I
−1:16D,19:4D
1:7I
−1:2I
−1:2D
−1:1D
−1:10I
−1:5I
1:17I
−2:7D
−20 −15 −10 −5 −1 1 5 10 15 20 25 30
30686 44101 29684 37836 39481 47003 235829 182080 184731 125747 200772 254844 318187 341359 316594 406353 246678 218774 19074 11769
28026 40269 27074 34822 4019 8238 14899 11394 14999 9403 29120 14857 11322 16929 95941 174345 181026 61265 1422 5406
0 24 23 1 17144 5982 42379 20055 18295 23671 15607 26910 31500 52300 32526 30886 12358 23673 1707 1123
399 72 3 0 255 9494 20415 40393 9929 6795 27853 39357 13899 17363 9860 16049 3947 19001 2201 475
1 0 0 1 8729 2059 16283 15679 13993 8713 9973 25874 15664 15450 19880 10779 10420 9704 539 416
0 0 0 0 891 915 2949 3822 40 2033 9013 6266 1962 71725 7092 17265 2188 5449 200 206
0 0 0 0 0 0 184 0 0 0 0 1 112760 299 1 1 0 1 0 0
0 0 0 0 14 502 16087 4096 45142 4961 5312 16803 3868 5200 1868 3541 281 1799 250 251
0 0 0 0 263 642 12035 6494 11553 3801 4552 2969 10307 15529 3229 6537 2274 11022 177 115
836 1479 1075 1322 739 2271 2236 1699 21722 724 6134 6823 3141 1248 11754 3465 404 4491 1945 561
0 0 0 0 0 192 14646 4961 371 9711 5900 1673 8978 4788 5813 6970 258 5068 515 178
0 0 0 0 0 159 123 2 3 181 2 1437 703 46176 2211 3438 928 1228 138 3
0 0 0 0 0 17 917 810 447 10 3551 3138 1907 2678 23356 1374 870 250 41 135
0 0 0 0 3 145 212 705 1 438 16259 731 172 1161 2255 5646 867 8 24 17
0 0 0 0 0 0 0 0 0 0 0 0 0 0 0 27586 0 0 0 0
0 0 0 0 0 0 0 18130 1 177 6 1888 255 354 242 472 0 1 0 21
0 0 0 0 3555 98 6297 192 6 306 909 112 1760 2635 2230 301 177 1323 57 0
0 0 0 0 0 106 557 1 661 9233 368 516 274 2980 2673 749 0 271 131 113
1 0 0 1 0 272 2961 3 2548 176 3099 146 875 970 649 904 1105 1538 3298 19
0 0 0 0 0 88 479 2 0 0 310 14364 249 2 1 633 0 2 289 5
0 0 0 0 0 144 270 526 4 749 3920 834 1318 16 4092 447 3 611 1225 26
0 0 0 0 0 0 0 1 0 0 3 0 2 1558 1 2 352 0 1291 0
0 0 0 0 0 4 19 18 1 3 247 65 493 7 3 386 3 6 1145 0
non−injected−1
non−injected−2
non−injected−3
non−injected−4
hyperplasia/decreased T4−1
hyperplasia/decreased T4−2
hyperplasia/decreased T4−3
hyperplasia/decreased T4−4
decreased T4−1
decreased T4−2
decreased T4−3
decreased T4−4
decreased T4−5
decreased T4−6
no phenotype−1
no phenotype−2
no phenotype−3
no phenotype−4
no phenotype−5
no phenotype−6
Percentage
0
25
50
75
100
A, T
AGGA, ATAC
ACGTA, AGGAA, GAGGA, TCCCG
A, G
AAACTGA, GGAAACT
CTGCAGATGA
AC, AG, GA, GG
AAACTGACAAAACTGGA, AGAAAAGAGAAAGAGGA, GAAACTGTAGAGGAAAA

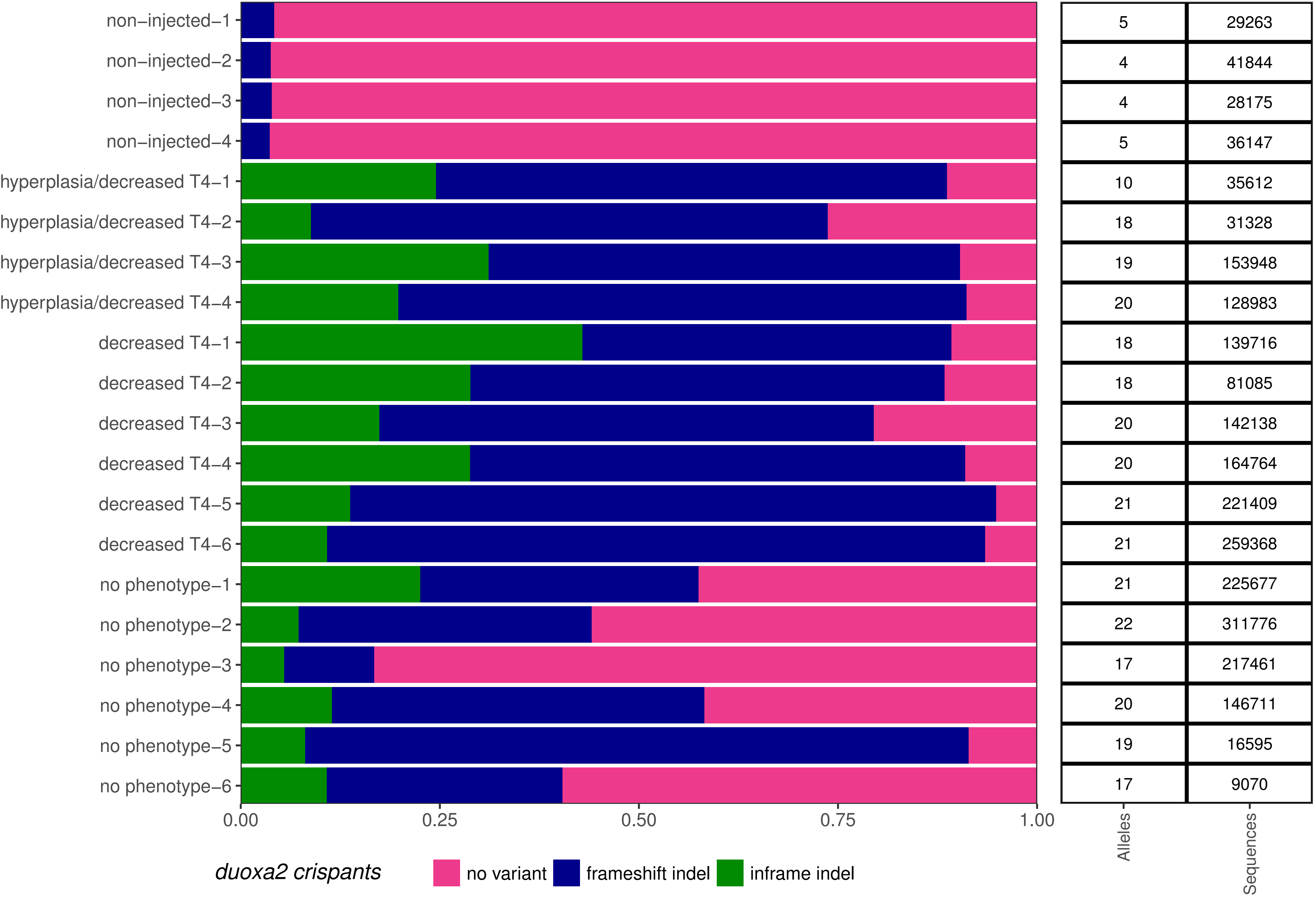
non−injected−1
non−injected−2
non−injected−3
non−injected−4
hyperplasia/decreased T4−1
hyperplasia/decreased T4−2
hyperplasia/decreased T4−3
hyperplasia/decreased T4−4
decreased T4−1
decreased T4−2
decreased T4−3
decreased T4−4
decreased T4−5
decreased T4−6
no phenotype−1
no phenotype−2
no phenotype−3
no phenotype−4
no phenotype−5
no phenotype−6
0.00
0.25
0.50
0.75
1.00
5 29263
4 41844
4 28175
5 36147
10 35612
18 31328
19 153948
20 128983
18 139716
18 81085
20 142138
20 164764
21 221409
21 259368
21 225677
22 311776
17 217461
20 146711
19 16595
17 9070
Alleles
Sequences
duoxa2 crispants
no variant
frameshift indel
inframe indel

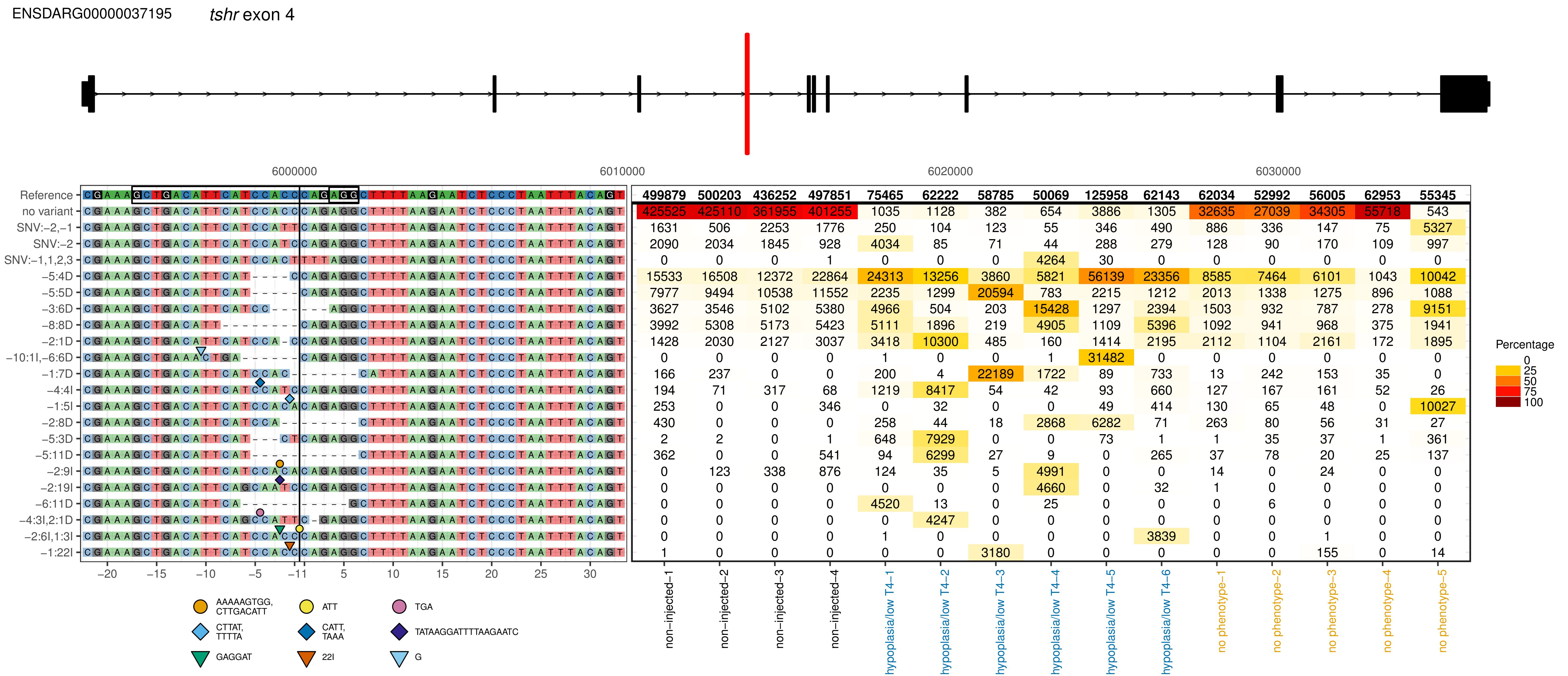

Variant	non-injected-1	non-injected-2	non-injected-3	non-injected-4	hypoplasia/low T4-1	hypoplasia/low T4-2	hypoplasia/low T4-3	hypoplasia/low T4-4	hypoplasia/low T4-5	hypoplasia/low T4-6	no phenotype-1	no phenotype-2	no phenotype-3	no phenotype-4	no phenotype-5
Reference	499879	500203	436252	497851	75465	62222	58785	50069	125958	62143	62034	52992	56005	62953	55345
no variant	425525	425110	361955	401255	1035	1128	382	654	3886	1305	32635	27039	34305	55718	543
SNV:−2,−1	1631	506	2253	1776	250	104	123	55	346	490	886	336	147	75	5327
SNV:−2	2090	2034	1845	928	4034	85	71	44	288	279	128	90	170	109	997
SNV:−1,1,2,3	0	0	0	1	0	0	0	4264	30	0	0	0	0	0	0
−5:4D	15533	16508	12372	22864	24313	13256	3860	5821	56139	23356	8585	7464	6101	1043	10042
−5:5D	7977	9494	10538	11552	2235	1299	20594	783	2215	1212	2013	1338	1275	896	1088
−3:6D	3627	3546	5102	5380	4966	504	203	15428	1297	2394	1503	932	787	278	9151
−8:8D	3992	5308	5173	5423	5111	1896	219	4905	1109	5396	1092	941	968	375	1941
−2:1D	1428	2030	2127	3037	3418	10300	485	160	1414	2195	2112	1104	2161	172	1895
−10:1I,−6:6D	0	0	0	0	1	0	0	1	31482	0	0	0	0	0	0
−1:7D	166	237	0	0	200	4	22189	1722	89	733	13	242	153	35	0
−4:4I	194	71	317	68	1219	8417	54	42	93	660	127	167	161	52	26
−1:5I	253	0	0	346	0	32	0	0	49	414	130	65	48	0	10027
−2:8D	430	0	0	0	258	44	18	2868	6282	71	263	80	56	31	27
−5:3D	2	2	0	1	648	7929	0	0	73	1	1	35	37	1	361
−5:11D	362	0	0	541	94	6299	27	9	0	265	37	78	20	25	137
−2:9I	0	123	338	876	124	35	5	4991	0	0	14	0	24	0	0
−2:19I	0	0	0	0	0	0	0	4660	0	32	1	0	0	0	0
−6:11D	0	0	0	0	4520	13	0	25	0	0	0	6	0	0	0
−4:3I,2:1D	0	0	0	0	0	4247	0	0	0	0	0	0	0	0	0
−2:6I,1:3I	0	0	0	0	1	0	0	0	0	3839	0	0	1	0	0
−1:22I	1	0	0	0	0	0	3180	0	0	0	0	0	155	0	14

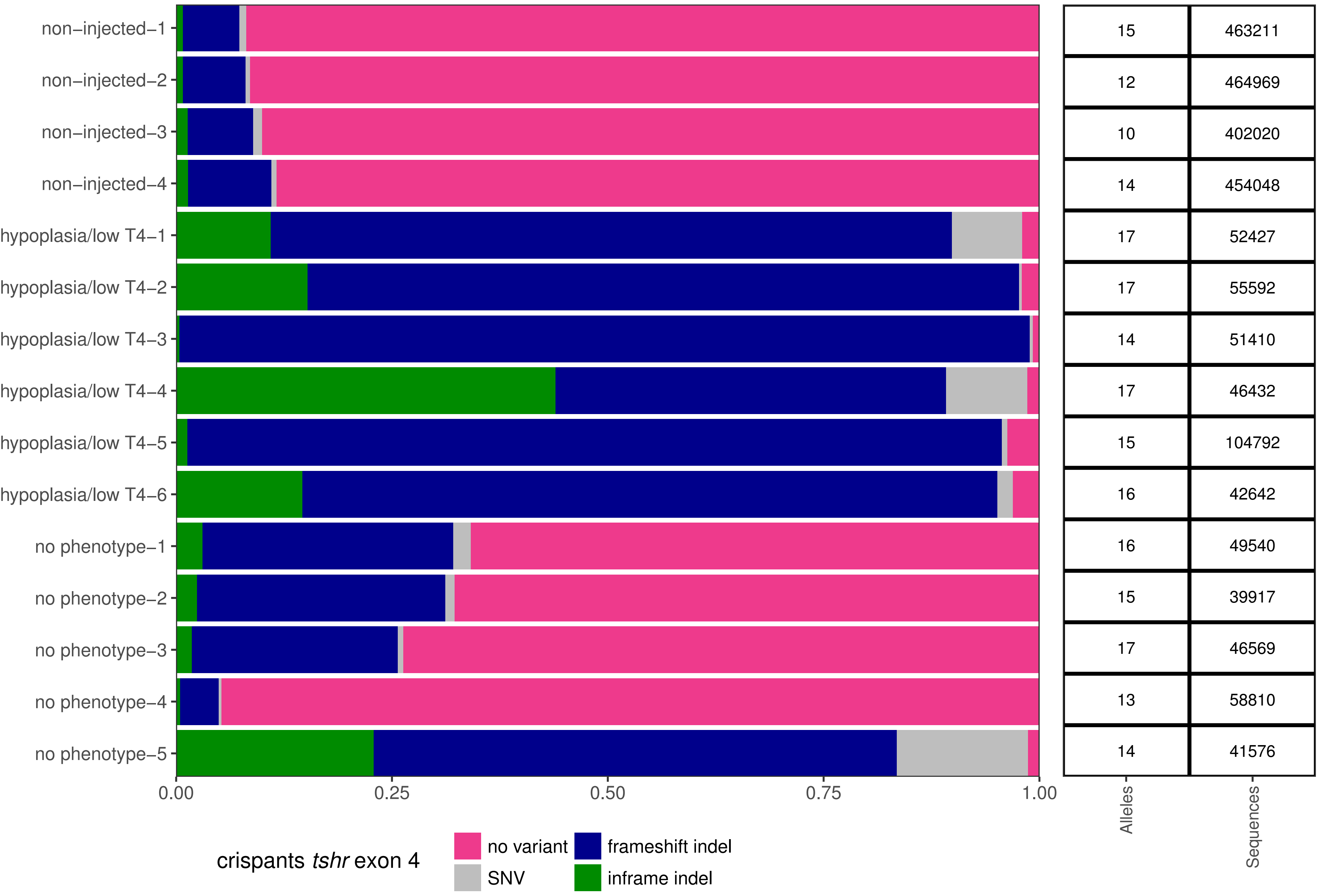

non–injected–1
non–injected–2
non–injected–3
non–injected–4
hypoplasia/low T4–1
hypoplasia/low T4–2
hypoplasia/low T4–3
hypoplasia/low T4–4
hypoplasia/low T4–5
hypoplasia/low T4–6
no phenotype–1
no phenotype–2
no phenotype–3
no phenotype–4
no phenotype–5
0.00
0.25
0.50
0.75
1.00
Alleles
Sequences
15 463211
12 464969
10 402020
14 454048
17 52427
17 55592
14 51410
17 46432
15 104792
16 42642
16 49540
15 39917
17 46569
13 58810
14 41576
crispants tshr exon 4
no variant
SNV
frameshift indel
inframe indel

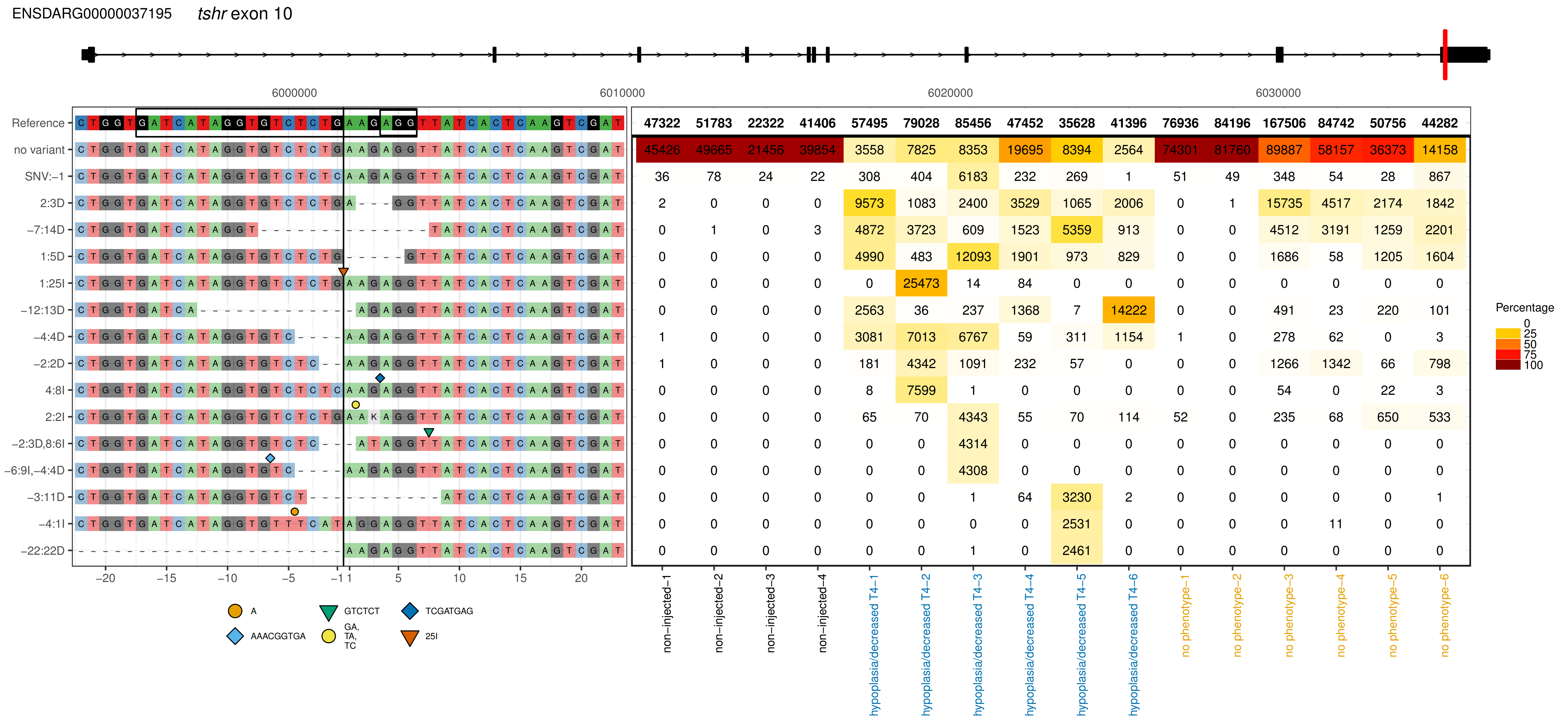
ENSDARG00000037195
tshr exon 10
6000000
6010000
6020000
6030000
Reference
no variant
SNV:−1
2:3D
−7:14D
1:5D
1:25I
−12:13D
−4:4D
−2:2D
4:8I
2:2I
−2:3D,8:6I
−6:9I,−4:4D
−3:11D
−4:1I
−22:22D
−20
−15
−10
−5
−1
1
5
10
15
20
A
GTCTCT
TCGATGAG
AAACGGTGA
GA, TA, TC
25I
47322 51783 22322 41406 57495 79028 85456 47452 35628 41396 76936 84196 167506 84742 50756 44282
45426 49665 21456 39854 3558 7825 8353 19695 8394 2564 74301 81760 89887 58157 36373 14158
36 78 24 22 308 404 6183 232 269 1 51 49 348 54 28 867
2 0 0 0 9573 1083 2400 3529 1065 2006 0 1 15735 4517 2174 1842
0 1 0 3 4872 3723 609 1523 5359 913 0 0 4512 3191 1259 2201
0 0 0 0 4990 483 12093 1901 973 829 0 0 1686 58 1205 1604
0 0 0 0 0 25473 14 84 0 0 0 0 0 0 0 0
0 0 0 0 2563 36 237 1368 7 14222 0 0 491 23 220 101
1 0 0 0 3081 7013 6767 59 311 1154 1 0 278 62 0 3
1 0 0 0 181 4342 1091 232 57 0 0 0 1266 1342 66 798
0 0 0 0 8 7599 1 0 0 0 0 0 54 0 22 3
0 0 0 0 65 70 4343 55 70 114 52 0 235 68 650 533
0 0 0 0 0 0 4314 0 0 0 0 0 0 0 0 0
0 0 0 0 0 0 4308 0 0 0 0 0 0 0 0 0
0 0 0 0 0 0 1 64 3230 2 0 0 0 0 0 1
0 0 0 0 0 0 0 0 2531 0 0 0 0 11 0 0
0 0 0 0 0 0 1 0 2461 0 0 0 0 0 0 0
Percentage
0
25
50
75
100
non−injected−1
non−injected−2
non−injected−3
non−injected−4
hypoplasia/decreased T4−1
hypoplasia/decreased T4−2
hypoplasia/decreased T4−3
hypoplasia/decreased T4−4
hypoplasia/decreased T4−5
hypoplasia/decreased T4−6
no phenotype−1
no phenotype−2
no phenotype−3
no phenotype−4
no phenotype−5
no phenotype−6

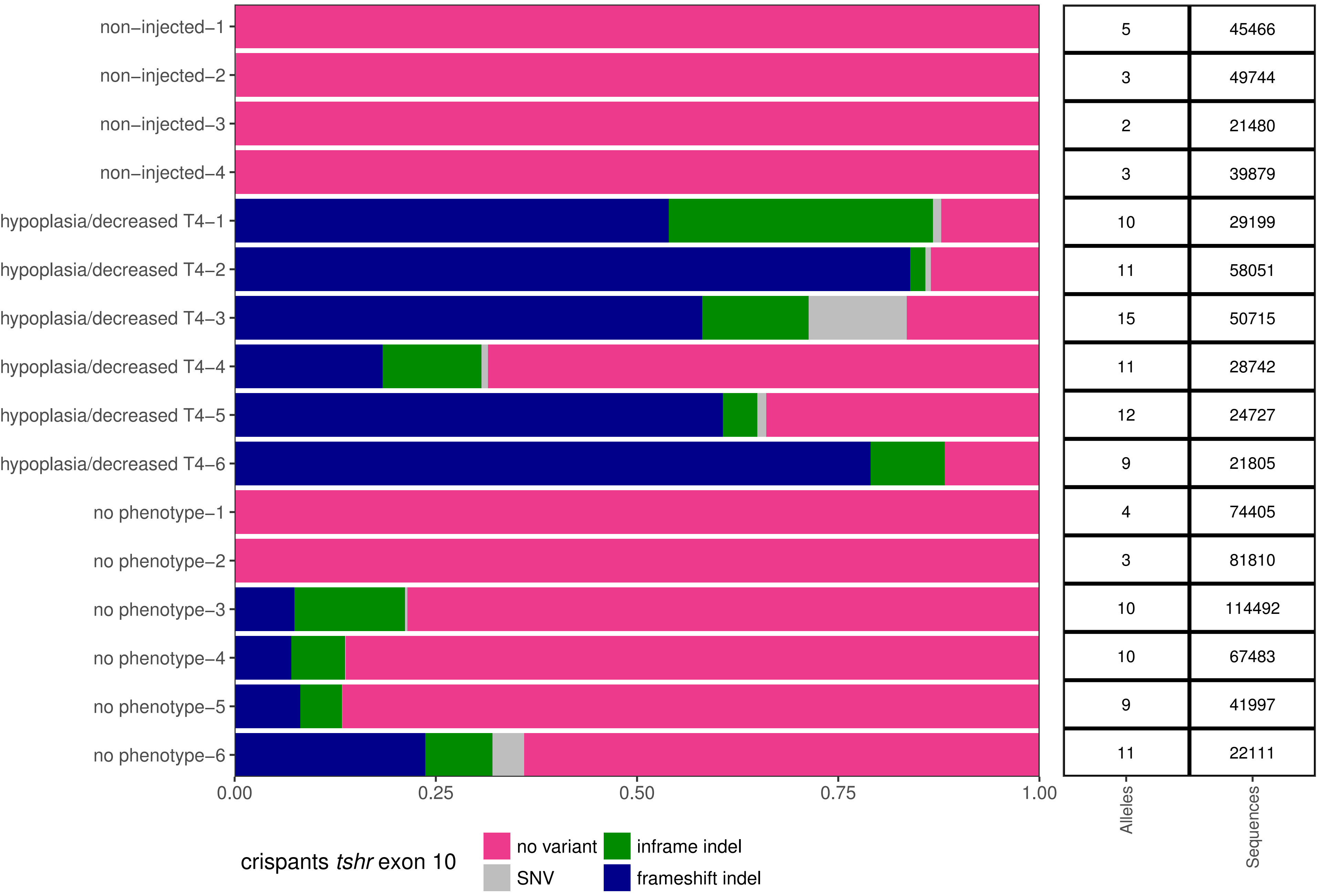

non−injected−1
non−injected−2
non−injected−3
non−injected−4
hypoplasia/decreased T4−1
hypoplasia/decreased T4−2
hypoplasia/decreased T4−3
hypoplasia/decreased T4−4
hypoplasia/decreased T4−5
hypoplasia/decreased T4−6
no phenotype−1
no phenotype−2
no phenotype−3
no phenotype−4
no phenotype−5
no phenotype−6
0.00
0.25
0.50
0.75
1.00
Alleles
Sequences
5 45466
3 49744
2 21480
3 39879
10 29199
11 58051
15 50715
11 28742
12 24727
9 21805
4 74405
3 81810
10 114492
10 67483
9 41997
11 22111
crispants tshr exon 10
no variant
inframe indel
SNV
frameshift indel

<u>Hiseq sequencing data tables and reads statistic.</u>

A)

<table>
<tr><td></td><td></td><td>e)</td><td></td></tr>
<tr><td rowspan="4">i)</td><td rowspan="4">h)</td><td rowspan="2">f)</td><td>a)</td></tr>
<tr><td>b)</td></tr>
<tr><td rowspan="2">g)</td><td>c)</td></tr>
<tr><td>d)</td></tr>
</table>

B)

<table>
<tr><td></td><td></td><td>nkx2.4b ex1</td><td></td></tr>
<tr><td rowspan="4">pool 1</td><td rowspan="4">barcode 1</td><td rowspan="2">27634</td><td>311923</td></tr>
<tr><td>290449</td></tr>
<tr><td rowspan="2">10%</td><td>284435</td></tr>
<tr><td>6014</td></tr>
</table>

A) Scheme representing the organisation of the data tables: a) Raw number of total reads pairs generated during Hiseq sequencing experiment. b) Number of sequences extracted from the raw data and used for analysis. c) Number of sequences successfully mapped on the genomic regions of interest. d) Number sequences unsuccessfully mapped on the genomic regions of interest. e) Genomic region of interest. f) Number of mapped reads corresponding to the genomic region of interest in (e). g) Proportion of a specific genomic region mapped reads on the total of Hiseq sequencing run reads. h) Barcode library reference for a specific set of samples. i) Hiseq sequencing run reference.

B) This example coming from the 1st barcode library generated and used in the first run of NGS experiment illustrates how one can read the data tables presented here. This NGS run generated 311923 reads, among those, 290449 could be used for analysis. Analysis showed that 284435 reads were mapped on the different regions of interest while 6014 could not be mapped. Out of those 284435 mapped reads 27634 could be mapped on the nkx2.4b exon1 targeted region. This number represents around 10% of the mapped reads from this experiment.

<u>Important note on the analysis:</u>

1) Note that the number of mapped reads reported in the following tables might be slightly higher than the total number of reads reported in CrispRVariant plots (first row). This is due to CrispRVariant analyses that excludes reads that do not fully span the target window used to summarize mutations for a given amplicon.
2) Note that the sum of the mapped reads might be slightly lower that the total number of mapped reads. This is due to off-target reads that could be mapped on the genome of the zebrafish but not on any of our genomic regions of interest.

		nkx2.4b ex1	*nkx2.4b* ex2	*pax2a*	*Duox* ex23	*duoxa* ex2	*tshr* ex10	*tshr* ex4	Unrellated genes	
Hiseq sequencing pool number 1	barcode1	27634	31620	26897	31979	30837	47331	N/A	87680	311923<br>290449
		10%	11%	9%	11%	11%	17%	N/A	31%	284435<br>6014
	barcode2	34248	42630	45534	44827	44120	51791	N/A	103777	383709<br>369209
		9%	12%	12%	12%	12%	14%	N/A	28%	367165<br>2044
	barcode3	18827	22136	30775	41469	29698	22327	N/A	79966	251393<br>246266
		8%	9%	13%	17%	12%	9%	N/A	33%	245383<br>883
	barcode4	20309	30839	35138	31209	37855	41416	N/A	94699	305698<br>294573
		7%	11%	12%	11%	13%	14%	N/A	32%	291627<br>2946
	barcode5	N/A	N/A	N/A	N/A	39548	N/A	N/A	237629	300855<br>279833
		N/A	N/A	N/A	N/A	14%	N/A	N/A	86%	277513<br>2320
	barcode6	N/A	N/A	N/A	N/A	47499	N/A	N/A	372064	517319<br>464391
		N/A	N/A	N/A	N/A	11%	N/A	N/A	87%	428781<br>35610
	barcode7	N/A	N/A	N/A	N/A	19555	N/A	N/A	146163	178411<br>168333
		N/A	N/A	N/A	N/A	12%	N/A	N/A	88%	166187<br>2146
	barcode8	N/A	N/A	N/A	N/A	11929	N/A	N/A	82630	104141<br>97050
		N/A	N/A	N/A	N/A	13%	N/A	N/A	87%	94703<br>2347

		nkx2.4b ex1	*nkx2.4b* ex2	*pax2a*	*Duox* ex23	*duoxa* ex2	*tshr* ex10	*tshr* ex4	Unrellated genes	
Hiseq sequencing pool number 2	barcode1	167333	157614	158825	113132	241650	66333	N/A	574859	1515679<br>1483691
		11%	11%	11%	8%	16%	4%	N/A	39%	1480170<br>3521
	barcode2	118677	103410	119374	60543	185064	80453	N/A	560086	1251645<br>1230863
		10%	8%	10%	5%	15%	7%	N/A	46%	1227690<br>3173
	barcode3	141879	153593	134503	54679	185980	94678	N/A	570641	1359848<br>1339316
		11%	11%	10%	4%	14%	7%	N/A	43%	1336161<br>3155
	barcode4	61284	75101	94298	73743	143437	48962	N/A	362124	887505<br>861800
		7%	9%	11%	9%	17%	6%	N/A	42%	859002<br>2798
	barcode5	132492	160579	180481	97663	203510	37577	N/A	525620	1358958<br>1340590
		10%	12%	13%	7%	15%	3%	N/A	39%	1338015<br>2575
	barcode6	162274	138317	160662	194754	256055	43682	N/A	547955	1530808<br>1508150
		11%	9%	11%	13%	17%	3%	N/A	36%	1503778<br>4372
	barcode7	233132	222020	94298	336642	329571	76950	N/A	861336	2382353<br>2337036
		10%	10%	4%	14%	14%	3%	N/A	37%	2330502<br>6534
	barcode8	230917	170524	132492	264755	359011	84203	N/A	919718	2331461<br>2288821
		10%	7%	6%	12%	16%	4%	N/A	40%	2282616<br>6205
	barcode9	250798	254568	97663	194652	318695	170679	N/A	944916	2436898<br>2405437
		10%	11%	4%	8%	13%	7%	N/A	39%	2399410<br>6027
	barcode10	285564	300872	202930	184546	412454	84971	N/A	1058048	2664509<br>2620212
		11%	12%	8%	7%	16%	3%	N/A	40%	2614461<br>5751
	barcode11	150956	153261	44130	103825	247152	50893	N/A	571775	1496017<br>1472009
		10%	10%	3%	7%	17%	3%	N/A	39%	1468389<br>3620
	barcode12	128164	205185	160662	128456	220169	46332	N/A	501095	1409077<br>1386484
		9%	15%	12%	9%	16%	3%	N/A	36%	1382764<br>3720

		nkx2.4b ex1	*nkx2.4b* ex2	*pax2a*	*Duox* ex23	*duoxa* ex2	*tshr* ex10	*tshr* ex4	Unrellated genes	
Hiseq sequencing pool number 3	barcode1	N/A	N/A	N/A	N/A	N/A	N/A	501161	N/A	540387<br>503599
		N/A	N/A	N/A	N/A	N/A	N/A	100%	N/A	501264<br>2335
	barcode2	N/A	N/A	N/A	N/A	N/A	N/A	500742	N/A	556699<br>504044
		N/A	N/A	N/A	N/A	N/A	N/A	100%	N/A	500843<br>3201
	barcode3	N/A	N/A	N/A	N/A	N/A	N/A	436873	N/A	474455<br>438540
		N/A	N/A	N/A	N/A	N/A	N/A	100%	N/A	436924<br>1616
	barcode4	N/A	N/A	N/A	N/A	N/A	N/A	498319	N/A	552900<br>501444
		N/A	N/A	N/A	N/A	N/A	N/A	100%	N/A	498645<br>2799
	barcode5	N/A	N/A	N/A	N/A	N/A	N/A	75723	258786	352047<br>339806
		N/A	N/A	N/A	N/A	N/A	N/A	23%	77%	334673<br>5133
	barcode6	N/A	N/A	N/A	N/A	N/A	N/A	62429	283350	400162<br>374983
		N/A	N/A	N/A	N/A	N/A	N/A	18%	82%	346415<br>28568
	barcode7	N/A	N/A	N/A	N/A	N/A	N/A	58862	311639	378762<br>370965
		N/A	N/A	N/A	N/A	N/A	N/A	16%	84%	370550<br>415
	barcode8	N/A	N/A	N/A	N/A	N/A	N/A	50357	250810	318162<br>302204
		N/A	N/A	N/A	N/A	N/A	N/A	17%	83%	301209<br>995
	barcode9	N/A	N/A	N/A	N/A	N/A	N/A	128219	262062	412041<br>393245
		N/A	N/A	N/A	N/A	N/A	N/A	33%	67%	390403<br>2842
	barcode10	N/A	N/A	N/A	N/A	N/A	N/A	63494	263287	337536<br>327778
		N/A	N/A	N/A	N/A	N/A	N/A	19%	81%	326823<br>955
	barcode11	N/A	N/A	N/A	N/A	N/A	N/A	62188	298405	371673<br>361404
		N/A	N/A	N/A	N/A	N/A	N/A	17%	83%	360630<br>774
	barcode12	N/A	N/A	N/A	N/A	N/A	N/A	53214	338873	412222<br>392883
		N/A	N/A	N/A	N/A	N/A	N/A	14%	86%	392125<br>758
	barcode13	N/A	N/A	N/A	N/A	N/A	N/A	56137	268006	333838<br>324973
		N/A	N/A	N/A	N/A	N/A	N/A	17%	83%	324234<br>739
	barcode14	N/A	N/A	N/A	N/A	N/A	N/A	63064	256022	330028<br>320154
		N/A	N/A	N/A	N/A	N/A	N/A	20%	80%	319160<br>994
	barcode15	N/A	N/A	N/A	N/A	N/A	N/A	55541	262144	324996<br>318001
		N/A	N/A	N/A	N/A	N/A	N/A	17%	83%	317731<br>270

NGS ORF alignments

pax2a exon 2 HiSeq sequencing most frequent alleles alignment:

```
a) ref rna: ATG|GAT|ATT|CAC|TGC|AAA|GCA|GAC|CCC|TTC|TCG|GCG|ATG|CAC|CGG|CAC|GGC|GGT|GTG|AAC|CAG|CTA|GGA|GGG|GTG|TTT|GTG|AAT|GGC|AGA|CCC|CTA|CCT|GAC|GTG|GTC|AGG|CAA|
   ref aa:   M   D   I   H   C   K   A   D   P   F   S   A   M   H   R   H   G   G   V   N   Q   L   G   G   V   F   V   N   G   R   P   L   P   D   V   V   R   Q
1) mut rna: ATG|GAT|ATT|CAC|TGC|AAA|GCA|GAC|CCC|TTC|TCG|GCG|ATG|CAC|CGG|CAC|GGC|GGT|GTG|AAC|CAG|GAG|GGG|TGT|TTG|TGA
   mut aa:   M   D   I   H   C   K   A   D   P   F   S   A   M   H   R   H   G   G   V   N   Q   E   G   C   L   *
2) mut rna: ATG|GAT|ATT|CAC|TGC|AAA|GCA|GAC|CCC|TTC|TCG|GCG|ATG|CAC|CGG|CAC|GGC|GGT|GTG|AAC|CAG|GGG|TGT|TTG|TGA
   mut aa :  M   D   I   H   C   K   A   D   P   F   S   A   M   H   R   H   G   G   V   N   Q   G   C   L   *
3) mut rna: ATG|GAT|ATT|CAC|TGC|AAA|GCA|GAC|CCC|TTC|TCG|GCG|ATG|CAC|CGG|CAC|GGC|GGT|GTG|AAC|CAG|CTT|AGG|AGG|GGT|GTT|TGT|GAA|TGG|CAG|ACC|CCT|ACC|TGA
   mut aa :  M   D   I   H   C   K   A   D   P   F   S   A   M   H   R   H   G   G   V   N   Q   L   R   R   G   V   C   E   W   Q   T   P   T   *
```

a) Reference transcript (ENSDARG00000028148).
1) Deletion of 4nt (AGCT). Occurrence: 14-50%.
2) Deletion of 7nt (CAGCTAGG). Occurrence: 1-13%.
3) Insertion of 1nt (T). Occurrence: 1-6%.

tshr exon 4 HiSeq sequencing most frequent alleles alignment:

```
a) ref rna:...AAG|CTG|ACA|TTC|ATC|CAC|CCA|GAG|GCT|TTT|AAG|AAT|CTC|CCT|AAT|TTA|CAG|TAT|CTA|GGA|TTA|TTC|AAC| ...
   ref aa:  ...K   L   T   F   I   H   P   E   A   F   K   N   L   P   N   L   Q   Y   L   G   L   F   N    ...
1) mut rna:...AAG|CTG|ACA|TTC|ATC|CAG|AGG|CTT|TTA|AGA|ATC|TCC|CTA|ATT|TAC|AGT|ATC|TAG
   mut aa:  ...K   L   T   F   I   Q   R   L   L   R   I   S   L   I   Y   S   I   *
2) mut rna:...AAG|CTG|ACA|TTC|ATC|AGA|GGC|TTT|TAA
   mut aa: ... K   L   T   F   I   R   G   F   *
3) mut rna:...AAG|CTG|ACA|TTC|ATC|CAC|CAT|TTA|AGA|ATC|TCC|CTA|ATT|TAC|AGT|ATC|TAG
   mut aa: ... K   L   T   F   I   H   H   L   R   I   S   L   I   Y   S   I   *
```

a) Reference transcript (ENSDARG00000037195).
1) Deletion of 4nt (CCAC). Occurrence: 6.6-44.6%.
2) Deletion of 5nt (CCACC). Occurrence: 1.6-35%.
3) Deletion of 7nt (CCAGAGG). Occurrence: 0-37.8%.

tshr exon 10 HiSeq sequencing most frequent alleles alignment:

```
a) ref rna:... TTG|AGT|GAT|AAC|CTC|TTC|AGA|GAC|ACC|TAT|GAT|CAC|CAG|GAG|TTT|TTC|GGC|AAC|GGA|CAT|TAT|CCC|ACT|TAT|TTT|GAC|AGA|CAT|GCA|GAT|GAG|AAC|ATG|GGT|TTC|GGT...
   ref aa: ...  L   S   D   N   L   F   R   D   T   Y   D   H   Q   E   F   F   G   N   G   H   Y   P   T   Y   F   D   R   H   A   D   E   N   M   G   F   G ...
1) mut rna:... TTG|AGT|GAT|AAC|CTC|   |AGA|GAC|ACC|TAT|GAT|CAC|CAG|GAG|TTT|TTC|GGC|AAC|GGA|CAT|TAT|CCC|ACT|TAT|TTT|GAC|AGA|CAT|GCA|GAT|GAG|AAC|ATG|GGT|TTC|GGT...
   mut aa: ...  L   S   D   N   L       R   D   T   Y   D   H   Q   E   F   F   G   N   G   H   Y   P   T   Y   F   D   R   H   A   D   E   N   M   G   F   G ...
2) mut rna:... TTG|AGT|GAT|AAC|CTA|TGA
   mut aa: ...  L   S   D   N   L   *
3) mut rna:... TTG|AGT|GAT|AAC|CAG|AGA|CAC|CTA|TGA
   mut aa: ...  L   S   D   N   Q   R   H   L   *
4) mut rna:... TTG|AGT|GAT|AAC|CTC|TTA|TCA|CTC|AGA|GAC|ACC|TAT|GAT|AAC|CAG|AGA|CAC|CTA|TGA
   mut aa: ...  L   S   D   N   L   L   S   L   R   D   T   Y   D   N   Q   R   H   L   *
5) mut rna:... TTG|AGT|GAT|AAC|CTC|TTG|ATC|ACC|AGG|AGT|TTT|TCG|GCA|ACG|GAC|ATT|ATC|CCA|CTT|ATT|TTG|ACA|GAC|ATG|CAG|ATG|AGA|ACA|TGG|GTT|TCG|GTG|AAA|CAC|TGA
   mut aa: ...  L   S   D   N   L   L   I   T   R   S   F   S   A   T   D   I   I   P   L   I   L   T   D   M   Q   M   R   T   W   V   S   V   K   H   *
```

a) Reference transcript (ENSDARG00000037195).
1) Deletion of 3nt (TCT), no frameshift induced. Occurrence: 1.4-16.7%.
2) Deletion of 14nt (ACCTCTTCAGAGAC). Occurrence: 0.8-15%.
3) Deletion of 5nt (CTCTT). Occurrence: 0.6-14.2%.
4) Insertion of 25nt (ATCACTCAGAGACACCTATGATAAC). Occurrence: 0-32.2%.
5) Deletion of 12nt (TCAGAGACACCTA). Occurrence: 0.1-34.4%

duox exon 23 HiSeq sequencing most frequent alleles alignment:

```
a) ref rna:...GTC|ATC|TAC|GCC|ATC|TCC|GCC|GGC|CTC|GCT|TTG|GAA|AGA|TGC|ATC|TAT|TAT|GGT|TTA|CAG|GCT|CAC|TCC|AGT|GGC|ATC|CCA|GAG|ACC|TCT|ATG|GTG|GGT|GTG|TTG|GTG|TCT|CGT
   ref aa: ... V   I   Y   A   I   S   A   G   L   A   L   E   R   C   I   Y   Y   G   L   Q   A   H   S   S   G   I   P   E   T   S   M   V   G   V   L   V   S   R
   ref rna:   GGC|TCA|GCG|GCC|GCC|ATT|TCG|TTC|CTG|TTC|CCC|TAC|ATG|CTG|CTG|ACT|GTG|TGC|CGA|AAC| ...
   ref aa:     G   S   A   A   A   I   S   F   L   F   P   Y   M   L   L   T   V   C   R   N   ...
1) mut rna:   GTC|ATC|TAC|GCC|GGC|           |CTC|GCT|TTG|GAA|AGA|TGC|ATC|TAT|TAT|GGT|TTA|CAG|GCT|CAC|TCC|AGT|GGC|ATC|CCA|GAG|ACC|TCT|ATG|GTG|GGT|GTG|TTG|GTG|TCT|CGT
   mut aa:     V   I   Y   A   G               L   A   L   E   R   C   I   Y   Y   G   L   Q   A   H   S   S   G   I   P   E   T   S   M   V   G   V   L   V   S   R
   mut rna:   GGC|TCA|GCG|GCC|GCC|ATT|TCG|TTC|CTG|TTC|CCC|TAC|ATG|CTG|CTG|ACT|GTG|TGC|CGA|AAC| ...
   mut aa:     G   S   A   A   A   I   S   F   L   F   P   Y   M   L   L   T   V   C   R   N   ...
2) mut rna:...GTC|ATC|TAC|GCC|ATC|TCG|CTT|TGG|AAA|GAT|GCA|TCT|ATT|ATG|GTT|TAC|AGG|CTC|ACT|CCA|GTG|GCA|TCC|CAG|AGA|CCT|CTA|TGG|TGG|GTG|TGT|TGG|TGT|CTC|GTG|GCT|CAG|CGG
   mut aa: ... V   I   Y   A   I   S   L   W   K   D   A   S   I   M   V   Y   R   L   T   P   V   A   S   Q   R   P   L   W   W   V   C   W   C   L   V   A   Q   R
   mut rna:   CCG|CCA|TTT|CGT|TCC|TGT|TCC|CCT|ACA|TGC|TGC|TGA
   mut aa:     P   P   F   R   S   C   S   P   T   C   C   *
3) mut rna:...GTC|ATC|TAC|GCC|ATC|TCG|CCG|GCC|TCG|CTT|TGG|AAA|GAT|GCA|TCT|ATT|ATG|GTT|TAC|AGG|CTC|ACT|CCA|GTG|GCA|TCC|CAG|AGA|CCT|CTA|TGG|TGG|GTG|TGT|TGG|TGT|CTC|GTG
   mut aa: ... V   I   Y   A   I   S   P   A   S   L   W   K   D   A   S   I   M   V   Y   R   L   T   P   V   A   S   Q   R   P   L   W   W   V   C   W   C   L   V
   mut rna:   GCT|CAG|CGG|CCG|CCA|TTT|CGT|TCC|TGT|TCC|CCT|ACA|TGC|TGC|TGA
   mut aa:     A   Q   R   P   P   F   R   S   C   S   P   T   C   C   *
```

a) Reference transcript (ENSDARG00000062632)
1) Deletion of 9nt (CGCCATCTC), no frameshift induced. Occurrence: 18-61.9%.
2) Deletion of 10nt (CTCCGCCGGC). Occurrence: 1.5-18.4%
3) Deletion of 1nt (C). Occurrence: 0-18%

Duoxa (duox2) exon 2 HiSeq sequencing most frequent alleles alignment:

```
a) ref rna:...GTC|AGT|TTC|CTC|TTC|ATT|CTA|CCG|GGA|ATA|CGT|GGC|AGA|TCG|AGA|TGG|TTT|TGG|ATG|TTC|CGG|ATA|TTC|ATT|AGC|TTA|TTT|ATT|GGA|GTT|GTT|TTA|GTT|GTG|CTT|AAT| ...
   ref aa: ... V   S   F   L   F   I   L   P   G   I   R   G   R   S   R   W   F   W   M   F   R   I   F   I   S   L   F   I   G   V   V   L   V   V   L   N   ...
1) mut rna:...GTC|AGT|TTC|CTC|TTC|TAC|CGG|GAA|TAC|GTG|GCA|GAT|CGA|GAT|GGT|TTT|GGA|TGT|TCC|GGA|TAT|TCA|TTA|GCT|TAT|TTA|TTG|GAG|TTG|TTT|TAG
   mut aa: ... V   S   F   L   F   Y   R   E   Y   V   A   D   R   D   G   F   G   C   S   G   Y   S   L   A   Y   L   L   E   L   F   *
2) mut rna:...GTC|AGT|TTC|CTC|TAC|CGG|GAA|TAC|GTG|GCA|GAT|CGA|GAT|GGT|TTT|GGA|TGT|TCC|GGA|TAT|TCA|TTA|GCT|TAT|TTA|TTG|GAG|TTG|TTT|TAG
   mut aa: ... V   S   F   L   Y   R   E   Y   V   A   D   R   D   G   F   G   C   S   G   Y   S   L   A   Y   L   L   E   L   F   *
3) mut rna:...GTC|AGT|TTC|CTC|   |ATT|CTA|CCG|GGA|ATA|CGT|GGC|AGA|TCG|AGA|TGG|TTT|TGG|ATG|TTC|CGG|ATA|TTC|ATT|AGC|TTA|TTT|ATT|GGA|GTT|GTT|TTA|GTT|GTG|CTT|AAT| ...
   mut aa: ... V   S   F   L       I   L   P   G   I   R   G   R   S   R   W   F   W   M   F   R   I   F   I   S   L   F   I   G   V   V   L   V   V   L   N   ...
4) mut rna:...GTC|AGT|TTC|CTC|TTC|TAT|TCT|ACC|GGG|AAT|ACG|TGG|CAG|ATC|GAG|ATG|GTT|TTG|GAT|GTT|CCG|GAT|ATT|CAT|TAG
   mut aa: ... V   S   F   L   F   Y   S   T   G   N   T   W   Q   I   E   M   V   L   D   V   P   D   I   H   *
5) mut rna:...GTC|AGT|TTC|CTC|CTT|   |CTA|CCG|GGA|ATA|CGT|GGC|AGA|TCG|AGA|TGG|TTT|TGG|ATG|TTC|CGG|ATA|TTC|ATT|AGC|TTA|TTT|ATT|GGA|GTT|GTT|TTA|GTT|GTG|CTT|AAT| ...
   mut aa: ... V   S   F   L   L       L   P   G   I   R   G   R   S   R   W   F   W   M   F   R   I   F   I   S   L   F   I   G   V   V   L   V   V   L   N   ...
```

a) Reference transcript (ENSDARG00000078962)
1) Deletion of 4nt (GAAT). Occurrence: 9.9-43.4%.
2) Deletion of 7nt (AGAATGA). Occurrence: 0.6-22.2%.
3) Deletion of 3nt (GAA), no frameshift induced. Occurrence: 4.4-22.1%
4) Insertion of 1nt (T). Occurrence: 0-21.1%.
5) Deletion of 3nt (ATG), no frameshift induced. Occurrence: 0-24.4%.

nkx2.4b exon 1 HiSeq sequencing most frequent alleles alignment:

```
a) Ref rna:... GGC|GCC|ACG|GCC|ACG|GCT|TGG|TAC|GGC|TCG|AAC|CCG|GAG|CCG|AGA|TAC|CCA|ACA|ATC|TCC|AGG|TTT|ATG|GGT|CCC|TCG|GCG|GGC|ATG|AAC|ATG|GGC|ACG|TTA|CCG|GGA|ATG|GAC|GCC|AGT...
   ref aa: ...  G   A   T   A   T   A   W   Y   G   S   N   P   E   P   R   Y   P   T   I   S   R   F   M   G   P   S   A   G   M   N   M   G   T   L   P   G   M   D   A   S ...
1) mut rna:... GGC|GCC|ACG|GCC|ACG|GTA|CGG|CTC|GAA|CCC|GGA|GCC|GAG|ATA|CCC|AAC|AAT|CTC|CAG|GTT|TAT|GGG|TCC|CTC|GGC|GGG|CAT|GAA|CAT|GGG|CAC|GTT|ACC|GGG|AAT|GGA|CGC|CAG|TAA
   mut aa: ...  G   A   T   A   T   V   R   L   E   P   G   A   E   I   P   N   N   L   Q   V   Y   G   S   L   G   G   H   E   H   G   H   V   T   G   N   G   R   Q   *
2) mut rna:... GCC|ACG|GCC|ACG|GCT|CGA|ACC|CGG|AGC|CGA|GAT|ACC|CAA|CAA|TCT|CCA|GGT|TTA|TGG|GTC|CCT|CGG|CGG|GCA|TGA
   mut aa: ...  A   T   A   T   A   R   T   R   S   R   D   T   Q   Q   S   P   G   L   W   V   P   R   R   A   *
3) mut rna:... AGA|AAC|GGC|GCC|ACG|GTA|CGG|CTC|GAA|CCC|GGA|GCC|GAG|ATA|CCC|AAC|AAT|CTC|CAG|GTT|TAT|GGG|TCC|CTC|GGC|GGG|CAT|GAA|CAT|GGG|CAC|GTT|ACC|GGG|AAT|GGA|CGC|CAG|TAA
   mut aa: ...  R   N   G   A   T   V   R   L   E   P   G   A   E   I   P   N   N   L   Q   V   Y   G   S   L   G   G   H   E   H   G   H   V   T   G   N   G   R   Q   *
4) mut rna:... AGA|AAC|GGC|GCC|ACG|GCT|CGA|ACC|CGG|AGC|CGA|GAT|ACC|CAA|CAA|TCT|CCA|GGT|TTA|TGG|GTC|CCT|CGG|CGG|GCA|TGA
   mut aa: ...  R   N   G   A   T   A   R   T   R   S   R   D   T   Q   Q   S   P   G   L   W   V   P   R   R   A   *
```

a) Reference transcript (ENSDARG00000104107).
1) Deletion of 5nt (GGCTT). Occurrence: 6-32.9%.
2) Deletion of 10nt (ACGGCTTGGT). Occurrence: 1-20%.
3) Deletion of 11nt (GGCCACGGCTT). Occurrence: 0-28.7%.
4) Deletion of 16nt (ACGGCCACGGCTTGGT). Occurrence: 2-27.7%.

nkx2.4b exon 2 HiSeq sequencing most frequent alleles alignment:

```
a) ref rna:... CAC|GCG|GCG|CCG|CGC|AGG|AAA|CGG|CGC|GTG|CTT|TTC|TCC|CAA|GCG|CAG|GTA|TAC|GAG|CTG|GAG|CGC|CGC|TTC|AAG|CAG|CAA|AAA|TAC|CTG|TCG|GCC|CCG|GAG|AGG|GAA|CAT|TTG|GCC|AGC|
   ref aa: ...  H   A   A   P   R   R   K   R   R   V   L   F   S   Q   A   Q   V   Y   E   L   E   R   R   F   K   Q   Q   K   Y   L   S   A   P   E   R   E   H   L   A   S
   ref rna:    ATG|ATC|CAT|CTA|ACC|CCG|ACG|CAG|GTC|AAG|ATC|TGG|TTC|CAG|AAC|CAC|AGG|TAC|AAA|ATG|AAG|CGG|GAG|GCG|AAG|GAT|AAA|GCG|TCC|CAG|CAG|CAG| ...
   ref aa:      M   I   H   L   T   P   T   Q   V   K   I   W   F   Q   N   H   R   Y   K   M   K   R   E   A   K   D   K   A   S   Q   Q   Q   ...
1) mut rna:... CAC|GCG|GCG|CGT|GCT|TTT|CTC|CCA|AGC|GCA|GGT|ATA|CGA|GCT|GGA|GCG|CCG|CTT|CAA|GCA|GCA|AAA|ATA|CCT|GTC|GGC|CCC|GGA|GAG|GGA|ACA|TTT|GGC|CAG|CAT|GAT|CCA|TCT|AAC|CCC|
   mut aa: ...  H   A   A   R   A   F   L   P   S   A   G   I   R   A   G   A   P   L   Q   A   A   K   I   P   V   G   P   G   E   G   T   F   G   Q   H   D   P   S   N   P
   mut rna:    GAC|GCA|GGT|CAA|GAT|CTG|GTT|CCA|GAA|CCA|CAG|GTA|CAA|AAT|GAA|GCG|GGA|GGC|GAA|GGA|TAA
   mut aa:      D   A   G   Q   D   L   V   P   E   P   Q   V   Q   N   E   A   G   G   E   G   *
2) mut rna:... CAC|GCG|GCG|CCG|CGC|AAG|GAA|ACG|GCG|CGT|GCT|TTT|CTC|CCA|AGC|GCA|GGT|ATA|CGA|GCT|GGA|GCG|CCG|CTT|CAA|GCA|GCA|AAA|ATA|CCT|GTC|GGC|CCC|GGA|GAG|GGA|ACA|TTT|GGC|CAG|
   mut aa: ...  H   A   A   P   R   K   E   T   A   R   A   F   L   P   S   A   G   I   R   A   G   A   P   L   Q   A   A   K   I   P   V   G   P   G   E   G   T   F   G   Q
   mut rna:    CAT|GAT|CCA|TCT|AAC|CCC|GAC|GCA|GGT|CAA|GAT|CTG|GTT|CCA|GAA|CCA|CAG|GTA|CAA|AAT|GAA|GCG|GGA|GGC|GAA|GGA|TAA
   mut aa:      H   D   P   S   N   P   D   A   G   Q   D   L   V   P   E   P   Q   V   Q   N   E   A   G   G   E   G   *
3) mut rna:... CAC|GCG|GCG|CCG|TGC|TTT|TCT|CCC|AAG|CGC|AGG|TAT|ACG|AGC|TGG|AGC|GCC|GCT|TCA|AGC|AGC|AAA|AAT|ACC|TGT|CGG|CCC|CGG|AGA|GGG|AAC|ATT|TGG|CCA|GCA|TGA
   mut aa: ...  H   A   A   P   C   F   S   P   K   R   R   Y   T   S   W   S   A   A   S   S   S   K   N   T   C   R   P   R   R   G   N   I   W   P   A   *
```

a) Reference transcript (ENSDARG00000104107).
1) Deletion of 17nt (CGGCGCCGCGCAGGAAA). Occurrence: 8.6-26.7%
2) Insertion of 1nt (T). Occurrence: 2.4-12.5%
3) Deletion of 16nt (CGCGCAGGAAACGGCG). Occurrence: 1-37%
